# Dipole–dipole interaction facilitates anion-rich solvation structures in polymer electrolytes for solid-state lithium metal batteries

**DOI:** 10.1126/sciadv.aej0175

**Published:** 2026-09-18

**Authors:** Zong-Yao Shuang, Chen-Zi Zhao, Xue-Yan Huang, Yu-Chen Gao, Pan Xu, Fang Fu, Young Oh, Wei-Jin Kong, Wen-Ze Huang, Yong-Feng Li, Zi-You Wang, Zi-Xuan Wang, Xiang Chen, Chen Ling, Jia-Qi Huang, Qiang Zhang

**Affiliations:** ^1^ Beijing Key Laboratory of Complex Solid-State Batteries, Department of Chemical Engineering, Tsinghua University, Beijing 100084, P. R. China.; ^2^ State Key Laboratory of Chemical Engineering and Low-Carbon Technology, Tsinghua University, Beijing 100084, P. R. China.; ^3^ AI Solid-State Battery Innovation Center, Yibin 644002, P. R. China.; ^4^ School of Chemistry, Engineering Research Center of Energy Storage Materials and Devices, Xi'an Jiaotong University, Xi'an 710049, P. R. China.; ^5^ School of Multidisciplinary Science, Beijing Institute of Technology, Beijing 100081, P. R. China.; ^6^ School of Materials Science and Engineering, Beijing Institute of Technology, Beijing 100081, P. R. China.; ^7^ Shanxi Research Institute for Clean Energy, Tsinghua University, Taiyuan 030032, P. R. China.; ^8^ Institute for Carbon Neutrality, Tsinghua University, Beijing 100084, P. R. China.

## Abstract

Polymer electrolytes are vital for safe and high-energy–density lithium metal batteries but face challenges from interfacial instability and Li dendrites. Here, we report a molecular design exploiting dipole–dipole interactions to tailor Li^+^ solvation structures and interfacial chemistry. Incorporating pentafluorophenyl acrylate into the polymer backbone enhances dipole interactions with 1,2-dimethoxyethane solvent, modulating the solvation structure and promoting anion entry into the primary coordination sphere. The anion-rich environment facilitates a durable, LiF-dominated solid electrolyte interphase, effectively suppressing dendrites and improving interfacial stability. The resulting electrolyte enables stable lithium plating/stripping over 2000 hours in symmetric cells and extends Li||LiNi_0.9_Co_0.05_Mn_0.05_O_2_ (NCM90) full cell cycling to 1000 cycles at 1.0 C. An anode-free Cu||NCM90 pouch cell delivers a high-energy–density of 579 Wh kg^−1^. This study underscores the critical role of dipole-mediated solvation structuring in polymer electrolytes and offers a generalizable approach toward high-performance, energy-dense solid-state batteries.

## INTRODUCTION

The rapid development of portable electronics, electric vehicles, and even electric vertical take-off and landing (eVTOL) aircraft has intensified the demand for energy storage systems that combine high-energy–density with uncompromised safety. Conventional graphite-based lithium-ion batteries are approaching a practical energy-density ceiling of ∼350 Wh kg^−1^ (*1*). In contrast, lithium metal batteries (LMBs), leveraging high theoretical specific capacity (3860 mAh g^−1^) and low redox potential of metallic lithium (Li), are promising candidates to surpass 500 Wh kg^−1^ (*2*, *3*). However, the flammability and leakage risks associated with commercial liquid electrolytes pose significant safety challenges (*4*–*6*).

Solid-state polymer electrolytes (SPEs) have emerged as promising candidates for LMBs due to their potential to eliminate electrolyte leakage and inhibit lithium dendrite growth, therefore delivering potential for enhanced safety and high-energy–density (*7*, *8*). In situ polymerization strategies further improve SPE–electrode interfacial contact and compatibility with existing battery manufacturing processes (*9*). Polyethylene oxide (PEO), featuring a linear ether-oxygen backbone structure, is one of the most widely studied ether-based polymer electrolytes but limited by low ionic conductivity (10^−8^ to 10^−7^ S cm^−1^) at room temperature (*10*), attributed to the restricted polymer chain segmental motion (*11*, *12*). Alternatively, the ring-opening polymerization of cyclic ethers, such as 1,3-dioxolane (DOL) (*13*) initiated by Lewis acid reagents, can form polyether-based electrolytes with a low glass transition temperature (*T*_g_) and high ionic conductivity (>10^−4^ S cm^−1^) (*14*). When combined with in situ polymerization process, they demonstrate conformal interfacial contact and low interfacial resistance (*15*). However, the Coulombic efficiency (CE) of cells using these polyether-based SPEs rarely exceeds 99%, primarily due to continuous electrolyte decomposition and lithium dendrite growth (*16*). Consequently, a stable and durable solid electrolyte interphase (SEI) is critical for mitigating side reactions and ensuring uniform lithium deposition (*17*–*19*).

The components of SEI highly depend on the solvation structure of Li^+^ (*20*). Key to engineering a favorable SEI is increasing the participation of anions in the first solvation shell of Li^+^, which suppresses solvent decomposition and promotes the formation of inorganic-rich interfacial layers (*21*, *22*). Adding additives such as FEC (*23*, *24*) and CuF_2_ (*25*) constitutes initial efforts in polymer electrolytes to construct a fluorine-rich SEI. Related insights from liquid-electrolyte studies, such as high-concentration electrolytes (HCEs) (*26*) and localized HCEs (LHCEs) (*27*), demonstrate that molecular-level control of Li^+^–solvent interactions can effectively tailor the SEI (*28*–*30*). In HCEs, an increased proportion of lithium bonds can stabilize the formation of large-sized aggregates (AGGs) (*31*). These AGGs, which incorporate more anions into the primary solvation shell of Li^+^, further modulate the composition of the SEI layer (*32*). In polymer systems, tris(2,2,2-trifluoroethyl)phosphate (*33*) has been used to construct an anion-derived solvation structure, while fluorinated polymer backbones preferentially decompose to form F-containing SEIs (*34*). Polymer–solvent interactions also influence Li^+^ solvation and transport (*35*). Nevertheless, how specific polymer backbones modulate Li^+^ coordination, solvation-sheath speciation, and ultimately SEI chemistry remains insufficiently understood. Compounding this gap, many demonstrations rely on coin-cell configurations with excess Li and generous electrolyte volumes, limiting relevance to high-energy practical devices. Therefore, establishing clear structure-solvation interphase relationships and validating performance under Li-lean conditions are both essential.

**Fig. 1.**
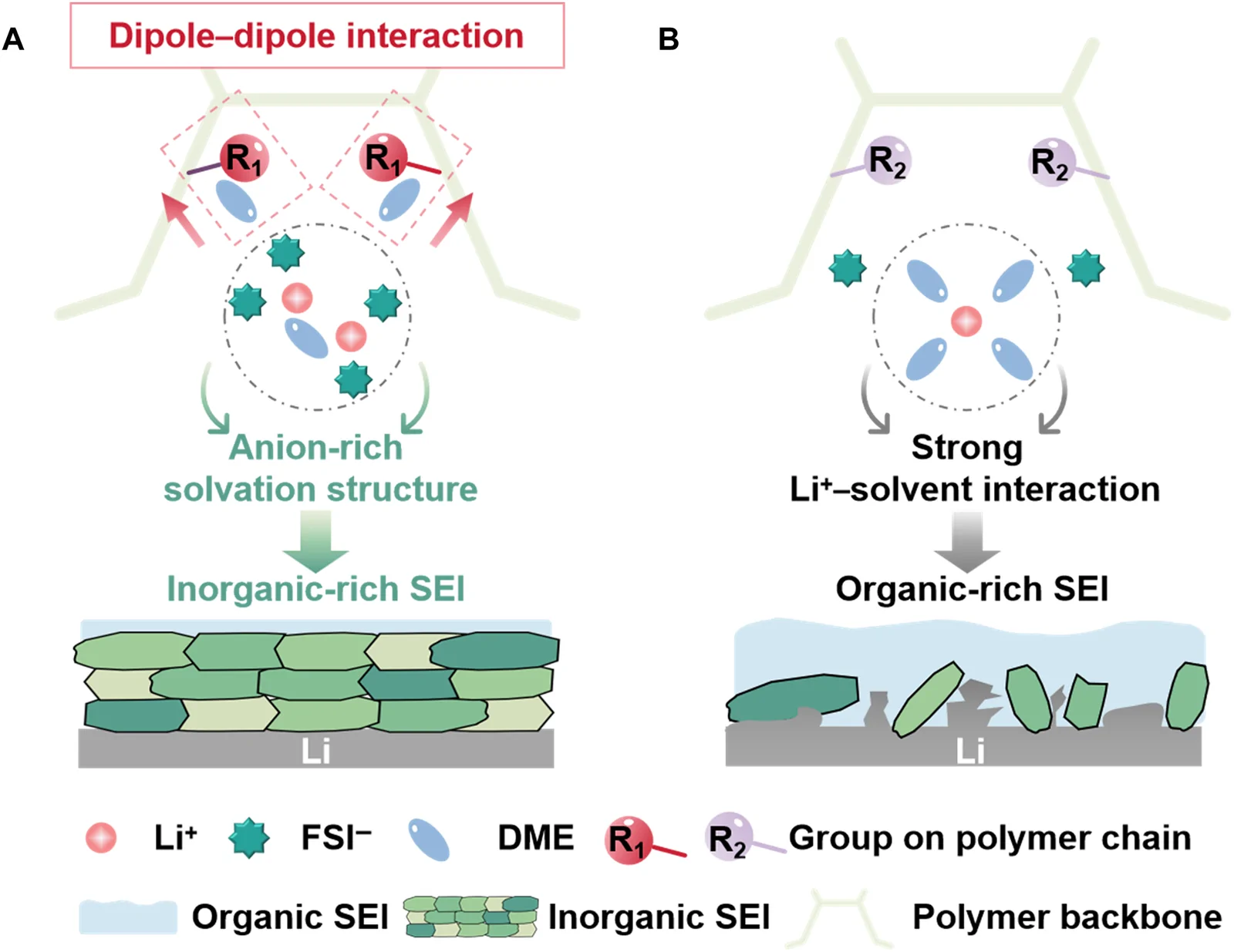
The design principle of SPEs with dipole–dipole interaction strategy. (**A**) Strong dipole–dipole interaction between polymer and solvent can effectively modulate the Li^+^ solvation structure by weakening Li^+^–solvent interaction and promoting anion coordination. Thereby, the improved anion-rich solvation structure can contribute to form a durable, inorganic-rich SEI. (**B**) With weak polymer-solvent dipole-dipole interaction, solvent-dominated Li^+^ solvation promotes unstable, organic-rich SEI.

In this study, by introducing pentafluorophenyl acrylate (PFA) into the polymer network, we report a molecular design strategy for in situ polymerized electrolyte (PFA-SPE) that leverages dipole–dipole interactions to tailor Li^+^ solvation structures and interfacial chemistry (fig. S1). The key effect in our system occurs before interfacial reactions. The PFA segments establish dipole–dipole interaction with 1,2-dimethoxyethane (DME), making a redistribution of the Li^+^ first solvation shell. This change increases anion participation and drives a solvation speciation enriched in AGGs over contact ion pairs (CIPs) (Fig. 1). The anion-rich environment promotes formation of a durable, LiF-dominated SEI that suppresses dendrite growth and stabilizes the interface, enabling highly reversible Li plating/stripping. Li||Li symmetric cells exhibit a stable long-term cycling life span exceeding 2000 hours. Meanwhile, Li||NCM90 cells maintain a stable cycling more than 1000 cycles. The anode-free pouch cell with a high areal capacity (6.1 mAh cm^−2^) delivers a high-energy–density of 579 Wh kg^−1^ and 1179 Wh liter^−1^, highlighting the potential of this dipole-mediated solvation-engineering strategy for practical high-energy LMBs.

## RESULTS

### Molecular interactions and solvation structure

To systematically explore the role of polymer–solvent dipole–dipole interaction in modulating the Li^+^ solvation environment, we synthesized in situ polymerized SPEs using two different acrylate monomers: PFA and 2-methoxyethyl acrylate (MeOA) (fig. S1). In each case, lithium bis(fluorosulfonyl)imide (LiFSI) was first dissolved in DME, followed by the addition of the chosen monomer and a cross-linking agent. Upon the introduction of the radical initiator 2,2′-azobis(2-methylpropionitrile) (AIBN) and heating at 60°C for 4 hours, the liquid precursor underwent polymerization, forming cross-linked polymer electrolyte (figs. S2 and S3).

Differential scanning calorimetry (DSC) results (fig. S4) indicate that no distinct *T*_m_ or *T*_c_ was observed for either of the two SPEs, demonstrating that both SPEs are amorphous (noncrystalline). Furthermore, two SPEs exhibit similar thermal properties: a low glass transition temperature (*T*_g_ = −24°C) and excellent thermal stability (120°C with 5% weight loss). In the absence of lithium salt and solvent, the rheological and mechanical properties of the two polymers were characterized via dynamic mechanical analysis (DMA) in compression mode (fig. S5). Within the temperature range of 5° to 60°C and frequency range of 1 to 30 Hz, both poly-PFA and poly-MeOA exhibit elastic solid behavior (storage modulus *G*′ > loss modulus *G*″). Under identical test conditions, the *G*′ of poly-PFA is higher than that of poly-MeOA, indicating that poly-PFA has superior mechanical properties.

**Fig. 2.**
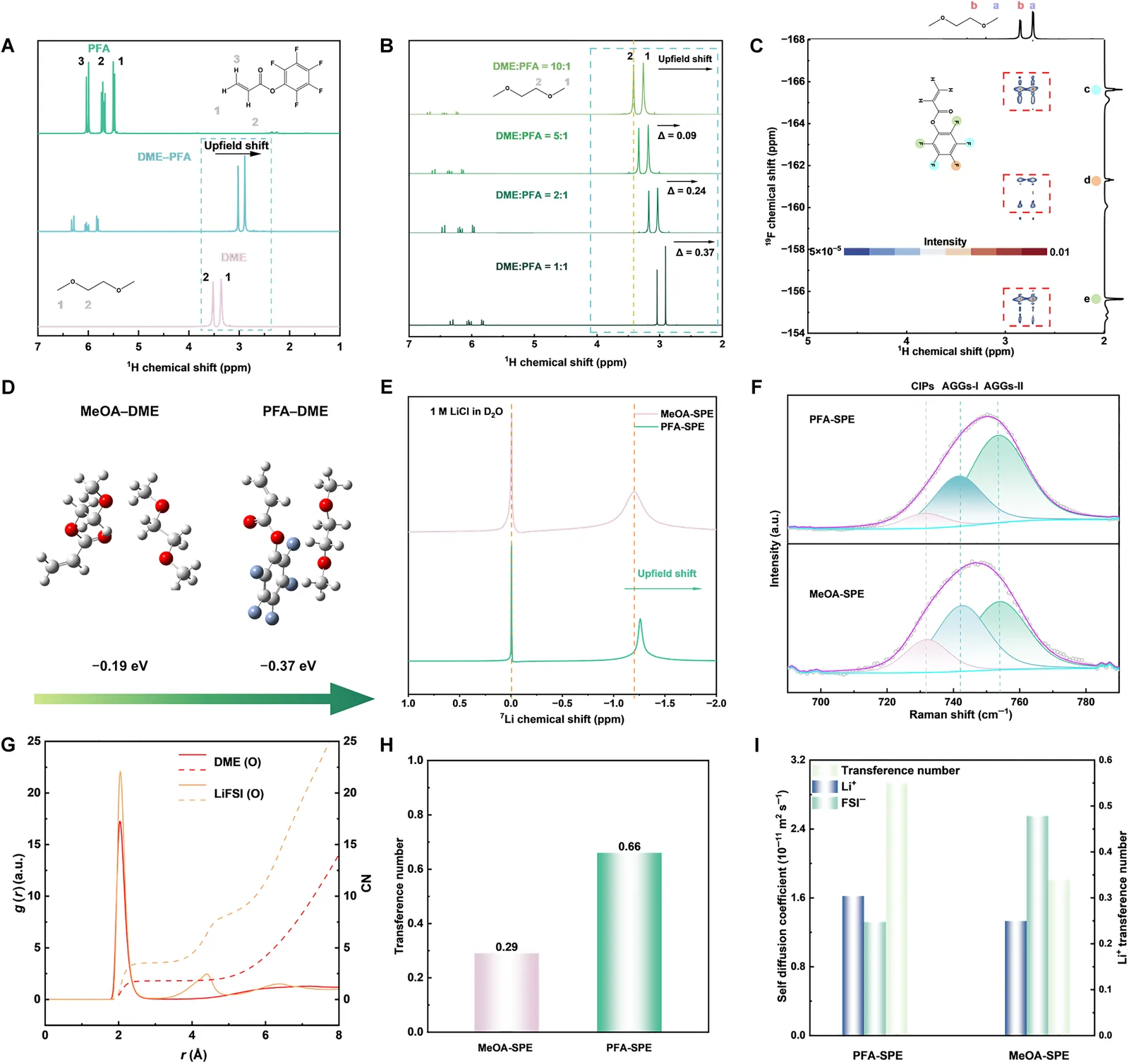
Molecular interaction and solvation structure of electrolytes. (**A**) ^1^H NMR results for DME, PFA, and DME–PFA mixed solution. (**B**) ^1^H NMR results for PFA and DME mixing solution with molar ratios ranging from 1:10 to 1:1. (**C**) 2D ^1^H–^19^F HOESY of poly-PFA and DME. (**D**) DFT results and geometries of binding energies for MeOA–DME and PFA–DME. Color mapping for elements: H, white; C, gray; O, red; F, cyan. (**E**) ^7^Li NMR results for MeOA-SPE and PFA-SPE. (**F**) Raman spectra of different electrolytes in the region of 690–780 cm^−1^. (**G**) Molecular dynamics (MD) simulated results of RDFs and CNs of the Li^+^–DME(O) and Li^+^–FSI^−^(O) in PFA-SPE. (**H**) Li^+^ transference number of different electrolytes tested by Bruce-Vincent method. (**I**) Self-diffusion coefficient of Li^+^ and FSI^−^ and the corresponding Li^+^ transference number measured by PFG NMR technique.

The molecular interactions were characterized by nuclear magnetic resonance (NMR) spectroscopy (*36*). For the PFA–DME solvent system (Fig. 2A), the ^1^H NMR signals of DME exhibit upfield shifts from 3.52 and 3.36 parts per million (ppm) to 3.02 and 2.89 ppm following the addition of PFA monomer (PFA:DME molar ratio = 1:1). The chemical shift changes explicitly indicate the occurrence of intermolecular interaction between PFA and DME. As the PFA:DME molar ratio increases from 1:10 to 1:1, the ^1^H NMR signals of DME gradually shift upfield (Fig. 2B), indicating that the intermolecular interactions between the two components were continuously enhanced with the increasing PFA content. At the same time, as the molar ratio of DME increases, the ^19^F NMR signals of PFA gradually shift downfield (fig. S6). In contrast, for the MeOA–DME mixed system, the ^1^H NMR signals of DME exhibit almost no change (fig. S7). Even as MeOA content increases (MeOA:DME molar ratio ranging from 1:10 to 1:1), the ^1^H NMR signals of DME remain unchanged at 3.52 and 3.36 ppm (fig. S8). This observation implies no significant electron cloud transfer between MeOA and DME, confirming a lack of intermolecular interactions between the two molecules.

These interactions trend persist in the SPEs. The ^1^H NMR signals of DME move from 3.36 to 3.13 ppm after the dissociation of LiFSI (fig. S9). In different SPEs, the ^1^H signals of DME exhibit distinct degrees of upfield chemical shifts, attributed to the varying intensities of dipole–dipole interaction. Respectively, the ^1^H NMR signals are recorded at 3.08 and 3.11 ppm for PFA-SPE and MeOA-SPE, indicating the strongest dipole-dipole interaction between poly-PFA and DME.

To directly probe these dipole–dipole interactions, we performed 2D ^1^H–^19^F heteronuclear overhauser effect spectroscopy (HOESY) (fig. S10). Specifically, cross-peaks between PFA and DME reveal that the hydrogen atoms at sites a and b of DME interact with the fluorine atoms at sites c, d, and e of PFA, with stronger interactions observed at sites c and e compared to site d. Notably, after in situ polymerization of PFA, these cross-peaks persist (Fig. 2C), confirming dipole–dipole interactions between poly-PFA and DME. Furthermore, from the results of density functional theory (DFT) calculations (Fig. 2D), the binding energies for PFA–DME and MeOA–DME are −0.37 and −0.19 eV, respectively, supporting the different intensity of dipole-dipole interactions.

The polymer–solvent interaction plays a crucial role in the Li^+^ solvation structure. The local electron environment of Li^+^ was probed by ^7^Li NMR. From the results, referenced to 1.0 M LiCl in D_2_O (0 ppm), the ^7^Li signals appear at −1.25 and −1.19 ppm in PFA-SPE and MeOA-SPE (Fig. 2E), respectively, indicating a higher electron cloud density around Li^+^ in PFA-SPE. Since the dipole–dipole interaction between poly-PFA and DME solvent attenuates the Coulombic attraction between DME and Li^+^, the enhanced electron cloud near Li^+^ originates from a higher proportion of anions participating in the solvation structure. Raman spectroscopy further elucidates the evolution of the solvation environment (Fig. 2F and fig. S11) (*37*). In SPEs, the solvation structure is mainly composed of CIPs (731 cm^−1^) and AGGs [AGGs-I (742 cm^−1^) and AGGs-II (755 cm^−1^)] (*38*). For PFA-SPE, strong dipole–dipole interaction drives AGGs-II to account for more than 60% of the solvation structure, while CIPs constitute only 7.9%. In contrast, MeOA-SPE, with almost no dipole–dipole interaction, exhibits high proportion of CIPs (16%) and low proportion of AGGs-II (44%). Molecular dynamics (MD) simulations were further used to elucidate the coordination environments of Li^+^ in distinct electrolytes. As a result, PFA-SPE exhibits the elevated Li–O (FSI^−^) coordination numbers, decreased Li–O (DME) coordination numbers, and higher Li^+^ diffusion coefficient (Fig. 2G, fig. S12, and table S1). From the experimental and calculation results, we confirmed the dipole–dipole interaction induced anion-dominated solvation structure in PFA-SPE. This solvation structure can contribute to form a durable, LiF-rich SEI and improve the Li anode cycling stability.

We further evaluated the effect of solvation structure on the ionic conduction behaviors of SPEs. With an increase in the fraction of AGGs-II in the solvation structure, PFA-SPE exhibits a higher ionic conductivity than that of MeOA-SPE at the measured temperature range from −10° to 60°C (fig. S13). The increase in anion proportion within the primary solvation shell is accompanied by enhancement of Li^+^ transference number, as verified by the Bruce-Vincent method (Fig. 2H and fig. S14) (*39*). For PFA-SPE, the fitting curves transitioned from the Vogel-Tammann-Fulcher (red line) to the Arrhenius relationship (green dashed line) (fig. S13), and the elevated Li^+^ transference number (0.66) indicates a variation of Li^+^ migration mechanism from vehicular transport to structural diffusion via anion ligand exchange, attributed to the anion-rich solvation structure. In addition, the self-diffusion coefficients of Li^+^ and FSI^−^ were probed using the pulsed-field gradient NMR (PFG NMR) technique (Fig. 2I and fig. S15) (*40*). In PFA-SPE, the Li^+^ diffusion coefficient exceeds that of FSI^−^, corresponding to a Li^+^ transference number greater than 0.5. The consistency between the two methods (Bruce-Vincent and PFG NMR) validates the results of variation trend of Li^+^ transference number.

### Microstructure of SEIs and morphology of deposited Li metal

**Fig. 3.**
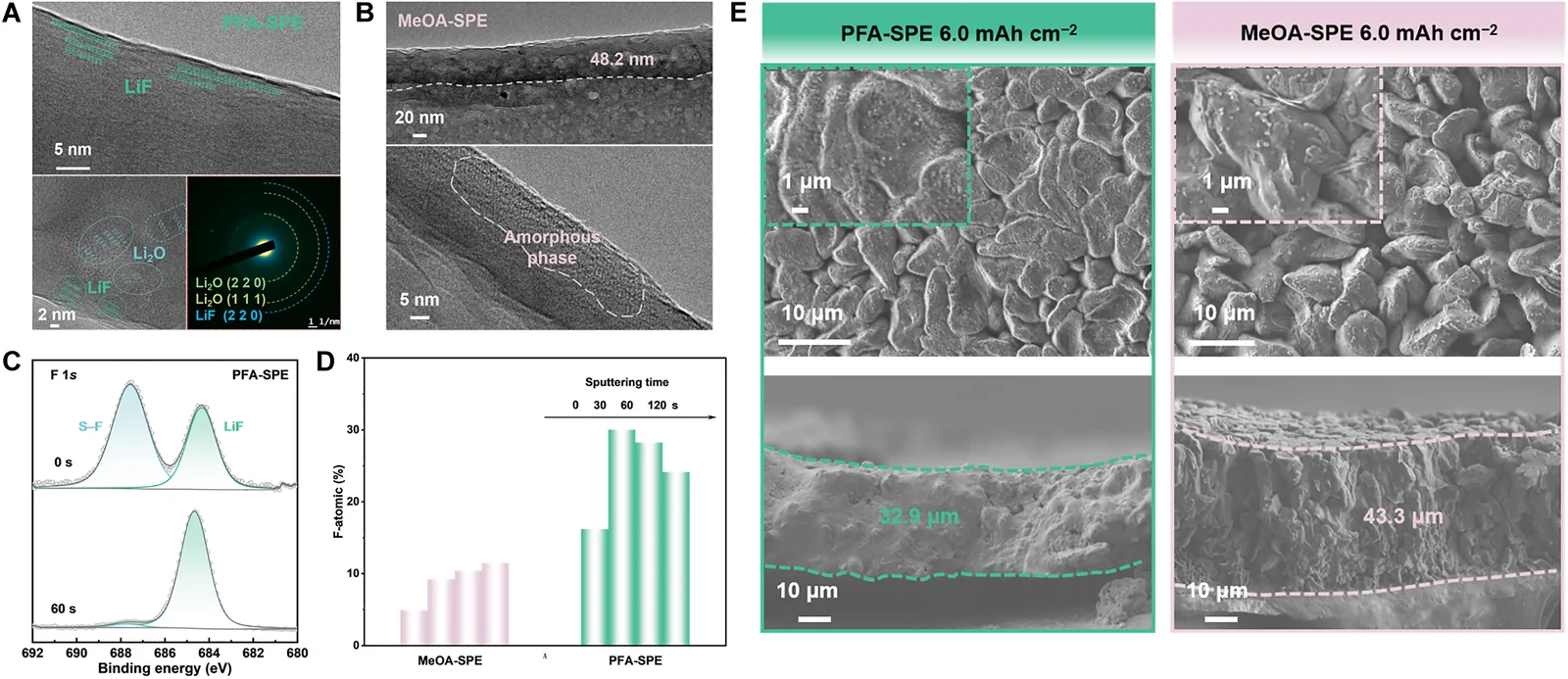
The microstructure of SEIs and the morphology of deposited Li metal. The Cryo-TEM and SAED images depict the structures and components of SEI layer for (**A**) PFA-SPE and (**B**) MeOA-SPE. (**C**) XPS F 1*s* spectra of the SEI layer for PFA-SPE. (**D**) Results of F element atomic ratios at different etching times for the SEI layer of different electrolytes by XPS. (**E**) Surface and cross section morphology of the deposited Li metal at an areal capacity of 6.0 mAh cm^−2^ after first plating characterized by SEM.

The solvation structure critically governs the compositional evolution of the anode SEI. The anion-dominated solvation environment preferentially yields inorganic-rich SEI layers, which improve the interfacial stability of Li metal anodes during prolonged cycling. To elucidate the influence of dipole–dipole interaction induced anion-rich solvation structure on anode–electrolyte interfacial composition and stability, the components and structure of SEI in different electrolytes were characterized by cryo–transmission electron microscopy (cryo-TEM) and x-ray photoelectron spectroscopy (XPS). Notably, PFA-SPE exhibits a uniform thin SEI layer (27.9 nm), whereas MeOA-SPE forms a thicker layer (48.2 nm) (Fig. 3B and fig. S16). Compositional analysis via cryo-TEM images and selected area electron diffraction (SAED) reveals that the SEI layer of PFA-SPE is dominated by continuous inorganic components, primarily LiF and Li_2_O (Fig. 3A). In contrast, the MeOA-SPE SEI consists predominantly of organic amorphous phases (Fig. 3B) (*41*). Furthermore, XPS was used to analyze the SEI compositions and their spatial distribution (Fig. 3, C and D) (*42*). The SEI layer of PFA-SPE exhibits a high LiF content in both the surface and inner layers, whereas the SEI layers formed with MeOA-SPE show insufficient LiF composition (figs. S18 and S19). The XPS results of O 1*s* and N 1*s* further reveal a higher content of Li_2_O and Li_3_N in the PFA-SPE SEI (fig. S20). The tailored solvation environment promotes the formation of a durable, inorganic-dominated SEI, which can effectively suppress dendrite growth and enhance interfacial stability.

On the basis of the distinct SEI layer compositions, the morphology of deposited Li metal was characterized by scanning electron microscopy (SEM). In fig. S20A, the initial nucleation overpotential of PFA-SPE is smaller than that of MeOA-SPE. Li nucleates and grows in smooth and island-like structures in the PFA-SPE, effectively avoiding the formation of whisker-like and dendritic lithium (fig. S21). Benefiting from the durable inorganic-rich SEI layer, even at high areal capacities (6.0 and 3.0 mAh cm^−2^), the first-cycle Li deposits in PFA-SPE exhibit a flat, compact, and homogeneous morphology, free of obvious pores or cracks (Fig. 3E and fig. S22). Such a uniform and dense morphology changes into a porous and loose structure with tiny Li granules for MeOA-SPE. Cross-sectional images further reveal the volume expansion during Li plating. At 6.0 mAh cm^−2^, the deposition thicknesses for PFA-SPE and MeOA-SPE are 32.9 and 43.3 μm, respectively (Fig. 3E and fig. S23), verifying that PFA-SPE enables dense and dendrite-free deposition with minimal volume expansion. Similar trends in deposition thickness and volume expansion are observed at 3.0 mAh cm^−2^ (figs. S22 and S23). Even after 50 cycles in symmetrical Li cells, lithium in PFA-SPE exhibits a flat, smooth, and dense morphology. In contrast, lithium in MeOA-SPE presents a rough and porous structure (fig. S24). In summary, dipole–dipole interaction, driving more anions into the solvation structure, promotes inorganic-rich SEI formation in PFA-SPE, leading to compact, uniform Li deposition and contributing to stable Li anode cycling performance.

### Reversibility and stability of Li anode

**Fig. 4.**
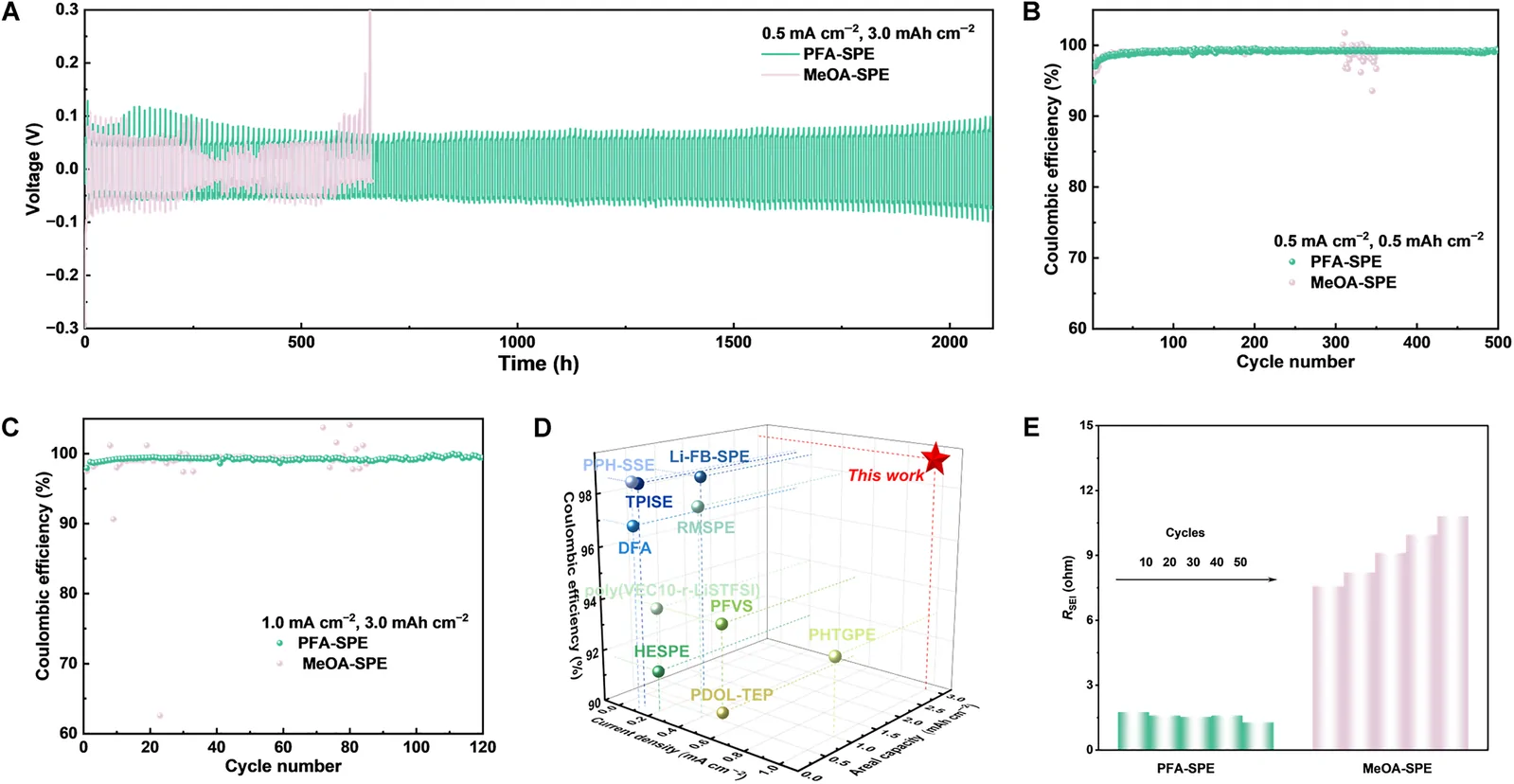
The reversibility and stability of Li anodes. (**A**) Cycling stability of SPEs in Li||Li symmetric cells measured at 0.5 mA cm^−2^ with 3.0 mAh cm^−2^. The CE of SPEs in Li||Cu half cells (**B**) at 0.5 mA cm^−2^ with 0.5 mAh cm^−2^ and (**C**) at 1.0 mA cm^−2^ with 3.0 mAh cm^−2^. (**D**) Comparison of Li||Cu cells’ performance with current works in terms of dimensions: CE, areal capacity, and current density. (**E**) *R*_SEI_ of Li||Li symmetric cells at varying cycles with SPEs.

We further test the impact of our designed solvation structure and SEI on the Li anode performance. The reversible Li plating/stripping behaviors of different electrolytes were evaluated using Li symmetric cells at 0.5 mA cm^−2^ with a high areal capacity of 3.0 mAh cm^−2^ (Fig. 4A). PFA-SPE exhibits stable cycling over 2000 hours with a steady overpotential of 70 mV and no short-circuit, indicating dendrite-free Li deposition and minimal side reactions during long-term cycling. In contrast, Li symmetric cells with MeOA-SPE show overpotential increases at 600 hours due to the unstable SEI layers. The critical current density (CCD) was tested with a gradually increasing current. Each charge/discharge cycle was conducted at a test capacity of 1.0 mAh cm^−2^, with the current density ranging from 0.2 to 5.0 mA cm^−2^. Since the same 32-μm polyethylene (PE) separator was used for both PFA-SPE and MeOA-SPE, both remained short-circuit–free up to 4.5 mA cm^−2^ (fig. S25). The CE test in Li||Cu cells further assessed Li anode reversibility under varying current densities and areal capacities. At 0.5 and 0.5 mAh cm^−2^ (Fig. 4B), Li||Cu cells with PFA-SPE demonstrate 500 cycles of performance with average CEs of 99.1%, confirming enhanced reversibility. However, cells with MeOA-SPE exhibit CE fluctuations after 300 cycles, attributed to micro-short circuits caused by Li dendrite growth. Under harsher conditions (1.0 and 3.0 mAh cm^−2^), MeOA-SPE shows CE fluctuations and drops within 50 cycles, whereas PFA-SPE maintains stable cycling over 100 cycles with an average CE of 99.1% (Fig. 4C). Modified Aurbach CE tests (*43*) further reveal a higher CE of 99.35% for PFA-SPE compared to 99.24% for MeOA-SPE (fig. S26). Increasing the areal capacity to ultrahigh 6.0 and 10.0 mAh cm^−2^, PFA-SPE can achieve stable cycling and high CE over 99.5%) (fig. S26). Because of the anion-dominated solvation structure and resulting durable inorganic-rich SEI, PFA-SPE achieves higher CE and more stable Li anode cycling performance. We made a comparison of Li||Cu cells’ performance with current works, outperforming current state-of-the-art Li||Cu cells in combined current density, areal capacity, and CE metric (Fig. 4D and table S2).

The galvanostatic electrochemical impedance spectroscopy (GEIS) and distribution of relaxation times (DRT) methods (*44*, *45*) were used to analyze the initial plating/stripping processes in Li||Cu cells with different electrolytes (figs. S28 and S29). Throughout both the plating and stripping process, PFA-SPE exhibits the lowest and the most stable interfacial impedance (*R*_i_), consistent with the presence of a durable SEI. For MeOA-SPE, interfacial impedance increases as metallic Li is stripped, attributed to an unstable SEI and accumulation of “dead Li.” EIS and DRT measurements on Li||Li symmetric cells further probe interfacial changes during long-term cycling (Fig. 4E and fig. S30). After 10, 20, 30, 40, and 50 cycles, the resistance of SEI (*R*_SEI_) of PFA-SPE shows a decreasing trend and remains consistently lower than that of the MeOA-SPE system, suggesting the gradual stabilization of the SEI layer. In contrast, *R*_SEI_ of MeOA-SPE increased continuously during cycling, reflecting SEI deterioration and persistent side reactions. These results highlight the critical role of molecular design strategy for anion-rich solvation structure and inorganic-rich SEI in stabilizing Li anodes, particularly under high areal capacities.

### Performances and applications of Li metal full cells

To verify the compatibility with high-voltage cathodes, the highest occupied molecular orbital (HOMO) and the lowest unoccupied molecular orbital (LUMO) energy levels of the monomers were calculated by DFT calculations. PFA shows the lowest HOMO energy level, suggesting good oxidative stability toward the cathode (fig. S31). The linear sweep voltammetry (LSV) measurements were used to characterize the electrochemical stability window of electrolytes (fig. S32A). MeOA-SPE shows a high-voltage tolerance of 4.6 V, whereas PFA-SPE exhibit enhanced oxidative stability (>4.8 V), making them suitable to match with high-voltage nickel-rich NCM cathodes. Furthermore, the electrochemical floating experiments, in which the potentials were held for 3.0 hours, and the current responses were recorded and were carried out to assess the high-voltage resistance (fig. S32B). Between 4.3 and 4.6 V, MeOA-SPE exhibited higher leakage currents compared to PFA-SPE (fig. S32B), indicating that MeOA-SPE has inferior oxidative stability relative to PFA-SPE. Subsequently, we quantified the accumulated capacity during the constant voltage hold period. The accumulated capacities of MeOA-SPE and PFA-SPE at 4.5 V were 0.0044 and 0.0019 mAh cm^−2^, respectively, quantitatively confirming the superior oxidative stability of PFA-SPE (fig. S32C).

According to the excellent compatibility with lithium metal and improved oxidative stability, the in situ polymerized PFA-SPE was evaluated in LMBs with various high-voltage cathodes. First, at a cutoff voltage of 4.3 V, Li|PFA-SPE|NCM811 cells deliver a specific capacity of 157.3 mAh g^−1^ at 0.5 C and 127.1 mAh g^−1^ at 1.0 C (fig. S33), maintaining 80.8 and 89.4% capacity retention after 300 cycles, whereas MeOA-SPE fails to stably cycle for 100 cycles. For rate performance (fig. S33D), PFA-SPE delivers higher discharge specific capacities than that of MeOA-SPE from 0.1 to 4.0 C. At 0.3-C charge and 1.0-C discharge, the initial discharge specific capacity of PFA-SPE is 152.2 mAh g^−1^, with capacity retention of 80.0% after 500 cycles (fig. S33E). Even at a high areal capacity of 21.5 mg cm^−2^, PFA-SPE still exhibits higher specific capacity (157.4 mAh g^−1^) and superior cycling stability (150 cycles) compared to MeOA-SPE (70 cycles) at 0.2 C (fig. S33F).

**Fig. 5.**
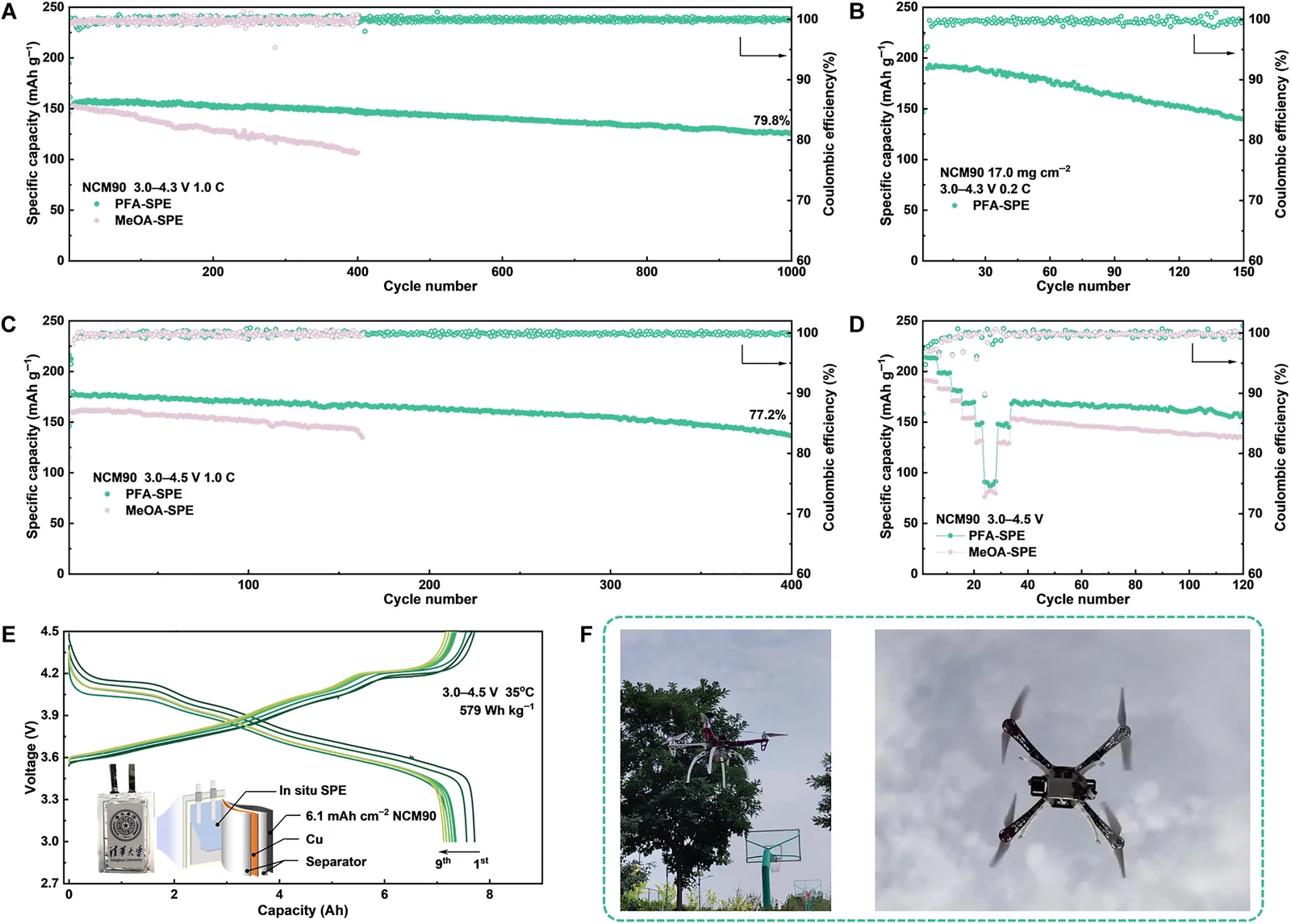
The performances and applications of full cells. The long-cycle performance of Li||NCM90 full cells at a cutoff voltage of 4.3 V at areal capacities of (**A**) 4.0 mg cm^−2^ and (**B**) 17.0 mg cm^−2^. The long-cycle performance (**C**) and rate performance (**D**) of Li||NCM90 full cells at a cutoff voltage of 4.5 V. (**E**) Charge/discharge curves of the anode-free pouch cell. (**F**) The takeoff process of the drone with our PFA-SPE pouch cells from the side view and bottom view.

When matched with NCM90, LMBs incorporating PFA-SPE exhibit minimal capacity decay, retaining 79.8% of the initial capacity (158.5 mAh g^−1^) after 1000 cycles (Fig. 5A and fig. S34). Notably, the cycling life span is more than twice that of the MeOA-SPE system, highlighting the critical role of PFA-SPE in enhancing long-term battery stability. At 0.3-C charge and 1.0-C discharge, Li|PFA-SPE|NCM90 cells deliver a specific capacity of 178.3 mAh g^−1^, and the capacity retention is 79.8% after 500 cycles (fig. S35). Increasing the areal capacity to 17.0 mg cm^−2^, PFA-SPE shows an initial discharge specific capacity of 193.2 mAh g^−1^ at 0.2 C with a capacity retention of 71.9% after 150 cycles (Fig. 5B). Furthermore, to evaluate the reversibility and stability of lithium metal, anode-free LMBs were assembled with NCM cathodes. Matched with NCM811 and NCM90 cathodes, PFA-SPE exhibits initial discharge specific capacities of 176.7 and 192.0 mAh g^−1^, respectively, and reaches the life span of 50 cycles, respectively (fig. S36).

At an elevated cutoff voltage of 4.5 V, PFA-SPE exhibits higher specific capacity of 177.8 mAh g^−1^ and excellent cycling stability of 400 cycles compared to MeOA-SPE (180 cycles) at 1.0 C (Fig. 5C). For rate performance, PFA-SPE delivers the specific discharge capacities of 213.6, 199.1, 181.5, 169.1, 147.6, and 90.9 mAh g^−1^ at 0.1, 0.2, 0.5, 1.0, 2.0, and 4.0 C, respectively, displaying better electrochemical performance and smaller electrochemical polarization than MeOA-SPE (Fig. 5D). The largely improved performance should be attributed to both the stable SEI and cathode electrolyte interphase (CEI), which inhibits the deterioration and continuous capacity decay during long cycles. For NCM90 cathodes after 100 cycles, the HRTEM images indicate that the CEI layer of PFA-SPE is uniform and thin (5.0 nm) (figs. S37, A and C), while the CEI layer of MeOA-SPE is inhomogeneous and thicker (16.0 nm) (figs. S37, B and D).

To meet the demand of ultrahigh-energy–density, an anode-free pouch cell with Cu current collector as anode, lean in situ polymerized electrolyte (polymer electrolyte to capacity ratio, E/C ratio, 1.2 g Ah^−1^) and high-loading NCM90 cathode was constructed by the multistacking method (Fig. 5E). Benefiting from the in situ polymerization process, PFA-SPE can successfully infuse in the void space of cathode particles (fig. S38). The detailed parameters were listed in table S3. Taking the mass and volume of all components into consideration (51.3 g and 25.2 cm^3^ in fig. S39), the designed pouch cell delivers a discharge capacity of 7.72 Ah and reaches a high initial specific energy of 579 Wh kg^−1^ and 1179 Wh liter^−1^ under 35°C, demonstrating the crucial role of PFA-SPE in improving the stability in practical lithium batteries. To demonstrate the practical application prospects, attempts were made to mount the designed batteries on a drone for a test flight. Three ∼6.0-Ah pouch cells (fig. S40) were connected in series and linked to the drone, enabling the drone to take off and hover briefly (Fig. 5F and movies S1 and S2), which greatly validates the commercial application potential of our designed high-energy–density battery system in the field of power batteries. For rate performance, PFA-SPE pouch cells exhibit the discharge capacity of 0.84 Ah at 0.4 C (25°C) and 0.85 Ah at 0.5 C (35°C) in the designed capacity of 1.15-Ah pouch cells (fig. S41, A and B). On the basis of the capacity of 1.15-Ah pouch cell at different rates, we can project the achievable energy density of 7.72-Ah pouch cell at corresponding rates (fig. S41C). From this projection, the 7.72-Ah pouch cell remains capable of delivering an energy density exceeding 400 Wh kg^−1^ at 0.5 C.

For the low-temperature performance, Li symmetric cells exhibited steady cycles during 500 hours at 0.2 mA cm^−2^ with an areal capacity of 0.5 mAh cm^−2^ (fig. S42A). Li||Cu cells with PFA-SPE demonstrate 100 cycles of performance with average CE of 98.5% at 0.5 and 0.5 mAh cm^−2^ (fig. S42B). For full cell performance, Li|PFA-SPE|NCM90 cells deliver a specific capacity of 156 mAh g^−1^ at 0.1 C in coin cell (fig. S42C). For pouch cell, the 1.12-Ah pouch cell at 25°C exhibited a capacity of 0.63 Ah at 0°C (fig. S42D).

## DISCUSSION

In summary, we proposed a cross-linked SPE through thermally initiated in situ polymerization of PFA and a cross-linking agent. The incorporation of PFA facilitates a strong dipole–dipole interaction between the polymer backbone and DME, which attenuates Li^+^–solvent coordination and promotes anion participation in the primary solvation shell. This tailored solvation environment yields an anion-rich solvation sheath and a stable, LiF-dominated SEI, enabling highly reversible Li plating/stripping. The PFA-based electrolyte delivers outstanding electrochemical performance, including >2000-hour cycling in Li||Li symmetric cells, and an average CE of 99.1% in Li||Cu cells. When coupled with high-voltage NCM90 cathodes, the PFA-SPE enables long-term cycling more than 1000 cycles at 1.0 C in coin cells at 25°C and achieves a high-energy–density of 579 Wh kg^−1^ in anode-free pouch cells at 35°C. The practical viability of this electrolyte system is further underscored by the successful demonstration of a drone flight powered by the corresponding pouch cells. Overall, this work establishes a rational molecular-design strategy for solvation structure engineering via dipole-dipole interactions, providing a promising pathway toward safe, high-energy-density LMBs.

## MATERIALS AND METHODS

### Experimental section

#### Materials availability

DME (99.9% purity) and LiFSI (99.9% purity) were purchased from Suzhou Duoduo Chemical Technology Co Ltd., China. Li foils (50 μm in thickness) were purchased from China Energy Lithium Co. PFA (98% purity), 2-methoxyethyl acrylate (MeOA, 98% purity), 2,2′-1,3,5-Tri-2-propenyl-1,3,5-triazine-2,4,6(1H,3H,5H)-trione (TAIC; 98% purity), and azobis(2-methylpropionitrile) (AIBN, 99% purity) were purchased from Shanghai Aladdin Biochemical Technology Co. Ltd. Lithium foils (50 μm in thickness) from China Energy Lithium Co. Ltd. were rolled on copper foils and punched into disks with a diameter of 15.0 mm. PE separator with a diameter of 19.0 mm was purchased from Asahi Kasei Technosystem Co. Ltd. LiNi_0.8_Co_0.1_Mn_0.1_O_2_ (NCM811, 5.0 and 21.5 mg cm^−2^) and LiNi_0.9_Co_0.05_Mn_0.05_O_2_ (NCM90, 4.0 and 17.0 mg cm^−2^) cathode were bought from Guangzhou Kelude Co. and were cut into disks with a diameter of 10.0 mm. LiNi_0.9_Co_0.05_Mn_0.05_O_2_ (NCM90, 6.1 mAh cm^−2^) cathode for pouch cells was purchased from Beijing WeLion New Energy Technology Co. Ltd.

### Preparation of SPEs

The solid polymer electrolytes (SPEs) were prepared by thermal-induced in situ polymerization. First, 1.14 g of LiFSI was dissolved in 0.66 g of DME solvent. After stirring for 1 hour and returning to room temperature, 0.148 g of PFA monomer, 0.052 g of TAIC cross-linking agent, and 0.002 g of AIBN initiator were added to the solution. Then, the polymerized precursor was stirred for 1 hour at 25°C and then heated at 60°C for 4 hours to obtain the polymer electrolyte (PFA-SPE). By the same procedure, the MeOA-SPE was synthesized by replacing the monomer with MeOA. For SPEs, monomer and cross-linking agent together account for 10 weight % of the whole electrolyte, and the molar ratio of the monomer and crosslinking agent is kept at 3:1.

### Coin cells assembly

All coin cells were assembled using standard CR2025 coin-type cells in an Ar-filled glove box with H_2_O < 0.1 ppm and O_2_ < 0.1 ppm. For Li||NCM90 full cells, first, the NCM90 cathode was placed in the cathode case and then 40 μl of the polymerized precursor was added to the surface of the NCM90 cathode. Subsequently, PE separator was placed on the NCM90 cathode, and another 40 μl of the polymerized precursor was added to the surface of the PE separator. Then, the Li metal anode was placed on the surface of the PE separator, and the coin cell was sealed by a manual battery hydraulic sealer sealing machine with a pressure of 1000 psi to enable close contact between each part. Last, the coin cell was heated at 60°C for 4 hours to induce the polymerization process. Other coin cells were assembled in the same way. Before testing, the coin cells were rested at 25°C for about 10 hours.

### Preparation of pouch cells

Cu||NCM90 pouch cells (7.0 × 4.0 cm^2^) were assembled in a dry room at a dew point of −60°C. For the pouch cell (579 Wh kg^−1^), without Li metal foil, the Cu current collectors (6-μm thick) were used as the anode. First, the NCM90 cathodes and Cu current collector were stacked layer-by-layer with a PE separator to assemble 20 anode|PE|cathode units, followed by packing in an aluminum-plastic film package. Then, the polymerized precursor was injected into the pouch cell in an Ar-filled glove box. Subsequently, the pouch cell was sealed under vacuum (−85 kPa) to eliminate residual oxygen and preserve the oxygen-free environment. Last, the pouch cell was rested at 25°C for about 12 hours and heated at 60°C for 4 hours. The detailed parameters of the pouch cell (579 Wh kg^−1^) are listed in table S3. For rate tests and drone flying tests, Li||NCM90 pouch cells were assembled in a dry room at a dew point of −60°C by the same procedure. The Li anode was rolled 50-μm-thick Li foils on both sides of Cu foils.

### Electrochemical tests

All coin cells were measured on a LAND multichannel battery testing system (Wuhan LAND Electronics Co. Ltd.) at 25°C. Li||NCM811 and Li||NCM90 coin cells were cycled in the voltage range of 3.0 to 4.3 V or 3.0 to 4.5 V at 0.05 C/0.1 C (1.0 C = 188.0 mAh g^−1^ for NCM811 and 1.0 C = 200.0 mAh g^−1^ for NCM90) for the initial activation cycle and then for the subsequent cycles. Pouch cells were cycled in the voltage range of 3.0 to 4.5 V at 0.025 C for the initial activation cycle and then at 0.05/0.1 C discharge. For the high-energy–density pouch cell, the anode current collector was prelithiated by charging at 25 mA and discharging at 85 mA. The discharge capacity and energy density were recorded charging at 25 mA and discharging at 85 mA at the first cycle. Subsequent cycles were performed at a charging current of 340 mA and a discharging current of 680 mA. The cycling performance of the pouch cells was tested under a fixing device to provide 1.0-MPa external pressure.

The Aurbach CE test of Li metal anodes was measured in Li||Cu cells. First, a certain amount of lithium (6.0 mAh cm^−2^) was plated and stripped as a “stabilization process” to form a passivation layer on electrodes. Then, a given amount of lithium (*Q*_T_, 6.0 mAh cm^−2^, 0.5 mA cm^−2^) was first deposited on Cu substrate as Li reservoir. Afterward, a smaller amount of this charge (*Q*_C_, 3.0 mAh cm^−2^, 1.0 mA cm^−2^) was plated and stripped for *n* (*n* = 20) cycles. Last, the remaining Li reservoir (*Q*_S_) was stripped until the cutoff voltage of 1.0 V. The average CE was calculated on the basis of

CEavg=nQC+QSnQC+QT (1)

The CCD was tested with a gradually increasing current. Each charge/discharge cycle was conducted at a test capacity of 1.0 mAh cm^−2^, with the current density ranging from 0.2 to 5.0 mA cm^−2^.

Electrochemical impedance spectroscopy (EIS) was performed using a Solartron 1470E electrochemical workstation. The frequency range was set from 10^6^ to 10^−1^ Hz with an amplitude of 10 mV. The GEIS was tested in a frequency range from 1 MHz to 0.1 Hz at an amplitude of 10 mV.

The ionic conductivity of MeOA-SPE at different temperatures was fitted into Vogel-Tammann Fulcher (eq. S2). σ is the ionic conductivity. *A* represents the preexponential factor. *R* is the Boltzmann constant. *T* is the testing temperature. *T*_0_ is the reference temperature, which was 50 K below the glass transition temperature (*T*_g_ − 50 K). *E*_a_ is the activation energy

$$\sigma =\text{Aexp}\left[-\frac{E_{a}}{R\left(T-T_{0}\right)}\right]$$ (2)

The ionic conductivity of PFA-SPE at different temperatures was fitted into Arrhenius (eq. S3). σ is the ionic conductivity. *A* represents the pre-exponential factor. *R* is the Boltzmann constant. *T* is the testing temperature. *E*_a_ is the activation energy.

$$\sigma =\text{Aexp}\left(-\frac{E_{a}}{\mathrm{RT}}\right)$$ (3)

The ESW of the SPEs was measured at 25°C by LSV on the Solartron 1470E electrochemical workstation at a scan rate of 0.5 mV s^−1^. The electrochemical floating experiments were preceded by holding for 3 hours at increasingly higher potentials, and the current response was recorded with the same battery model.

Li^+^ transference number (*t*_+_) was performed on the Solartron 1470E electrochemical workstation. The *t*_+_ was measured using the potentialstatic polarization method established by Bruce and Vincent using the Li||Li cell configuration. *t*_Li+_ was calculated using equation

$$t_{+}=\frac{I_{s}\left(∆V-I_{0}R_{i}^{0}\right)}{I_{0}\left(∆V-I_{s}R_{i}^{s}\right)}$$ (4)

In Eq. 4, ∆*V*, *I*_0_, and *I*_s_ represent the polarization voltage, initial current, and steady-state current, respectively. $R_{i}^{0}$ and $R_{i}^{s}$ denote the charge transfer resistance at the initial and steady state, respectively.

### Sample characterizations

The electrolyte was analyzed using liquid NMR on a JNM-ECZ400S spectrometer. We used the coaxial NMR tubes, where the inner tube containing the standard NMR solvent (dimethyl sulfoxide-d6 or D_2_O) is inserted into an outer tube holding the sample solution. After adding the liquid precursor into the NMR tube, the polymer or SPEs were formed via in situ solidification process directly within the NMR tube. For ^7^Li NMR, the D_2_O solution of 1 M LiCl was added to the inner NMR tube, where D_2_O served as the deuterated reagent. The ^7^Li NMR shifts were calibrated via the internal standard method, with the ^7^Li chemical shifts of 1 M LiCl in D_2_O set as 0 ppm. The 2D ^1^H-^19^F HOESY experiment was performed on a 600-MHz NMR spectrometer operating at 600.21 MHz for ^1^H and 564.76 MHz for ^19^F. The spectrum was acquired with 1280 complex points in the direct ^1^H dimension and 32 increments in the indirect ^19^F dimension. For each increment, 16 scans were accumulated using a relaxation delay of 1.5 s and a mixing time of 1 s. The PFG NMR technique was applied to investigate the Li^+^ transference number of polymer electrolytes. ^7^Li and ^19^F PFG measurements were performed to determine the self-diffusion coefficients of Li^+^ and FSI^−^. Raman measurements were performed on a Horiba HR 800 spectrometer equipped with a 785-nm laser as the excitation source. Fourier transform infrared (NICOLET 6700) was applied to characterize the structure of the materials in the attenuated total reflection mode.

The thermogravimetric analysis was tested using a Q5000IR (TA Instruments) from room temperature to 500°C under an N_2_ atmosphere at a temperature increase of 5°C min^−1^. DSC was tested using a Q5000IR (TA Instruments) from −90° to 100°C at a temperature increase of 3°C min^−1^. DMA was performed using a Q800 (TA Instruments) in compression mode.

The electrode morphology characterization was conducted using SEM (JSM 7401F at 5.0 kV) and transmission electron microscopy (TEM; JEOL Ltd. at 120 kV). The cycled coin cells were disassembled in the Ar-filled glove box and Li metal anode was washed three times with DME solvent to remove the residual electrolytes. For the SEM tests, samples of Li metal were transferred using a sealed container without exposing to air and moisture. For the cryo-TEM tests, Li metal was deposited on a Cu grid with a capacity of 0.5 mAh cm^−2^ at 0.2 mA cm^−2^. Using a sealed container, Cu grid was directly mounted to the holder (Gatan 698, cryo-transfer holder), and the cryo-TEM holder was quickly inserted into the TEM chamber. Then, liquid nitrogen was poured into the cryo-TEM holder until the sample temperature dropped below −170°C. All cryo-TEM images were taken at cryogenic temperature (−170°C).

The SEI was examined using XPS with an ESCALAB Xi^+^ spectrometer from ThermoFisher Scientific in the United Kingdom. Detailed SEI spectra were acquired through Ar^+^ sputtering at 2 keV (30 nm/min SiO_2_/Si) for 60 s. Precise binding energy data correction was performed using the carbon peak in the C 1*s* spectra (284.8 eV). Samples of Li metal were transferred for XPS under Ar protection.

### Computational details

#### DFT calculations

All DFT calculations were performed in the Gaussian (G16) program (*46*) using Becke’s three-parameter hybrid method using the Lee–Yang–Parr correlation functional (B3LYP) (*47*) at 6-311++G(d, p) level. The DFT calculations were carried out with solvation conditions parameterized by the experimentally measured dielectric constants of the two systems. The dielectric constant (ε_r_) of PFA and MeOA monomers are 12.15 and 11.43, respectively. Frequency analysis was conducted to ensure the ground state of molecular structures. Based on the optimized structures, the HOMO and the LUMO energy levels were analyzed using the natural bond orbital (NBO) (*48*) theory.

The binding energy ($E_{b}$) between a DME molecule and a monomer (MeOA/PFA) was defined as follows

$$E_{b}=E_{\text{DME}–\text{monomer}}-E_{\text{DME}}-E_{\text{monomer}}$$ (5)

where $E_{\text{DME}-\text{monomer}}$, $E_{\text{DME}}$, and $E_{\text{monomer}}$ denote the total energy of the cluster, DME, and monomer, respectively.

### MD simulations

MD simulations were performed using the Large Scale Atomic/Molecular Massively Parallel Simulator (LAMMPS) code (*49*). Three models were constructed: (i) PFA-SPE system with 400 LiFSI, 480 DME and 12 Poly-PFA molecules (total number of atoms: 892); (ii) MeOA-SPE system with 400 LiFSI, 480 DME and 12 Poly-MeOA molecules (total number of atoms: 892). The polymers are simulated using the polymerization of three monomers. The OPLS-AA force field (*50*) parameters for solvents were generated by the LigParGen web server (*51*), except that the advanced restrained electrostatic potential (RESP2) atomic partial charges (*52*) were assigned to each atom, which were obtained on the basis of electrostatic potential (ESP) charges using the Multiwfn program (*53*). Also, the parameters for Li^+^ and FSI^−^ were obtained from (*54*) and (*55*) respectively. The initial atomic coordinates were generated with the Packing Optimization for Molecular Dynamics Simulations (Packmol) program (*56*). The snapshots of MD boxes and the solvation structures were visualized by VESTA (*57*).

Periodic boundary conditions were applied in all three dimensions. A cutoff of 12 Å was used for both van der Waals interactions and long-range correction (particle-particle particle-mesh) of Coulombic interactions. The time step was set at 1 fs. All electrolyte models are first equilibrated in NPT ensemble (isobaric-isothermal ensemble) using the Parrinello-Rahman barostat (*58*) for 2 ns to maintain a temperature of 298 K with time constants of 0.1 ps and a pressure of 1 atm with time constant of 1 ps. After that, the system was heated from 298 to 450 K within 0.5 ns and maintained at 450 K for 1 ns, followed by annealing from 450 to 298 K within 0.5 ns. The system was then equilibrated at 298 K in NPT ensemble for another 4 ns. A 7-ns production run was finally conducted for all systems in a canonical (NVT) ensemble under Nose-Hoover thermostat (*59*, *60*). The radical distribution function, solvation structures and coordination number were analyzed using the Python MDTraj package (*61*) by processing all 5000 frames of the last 5-ns simulations.

The solvation structures were analyzed using the Python MDTraj package (*61*) by traversing all 5000 frames of the last 5-ns simulations. The radial distribution function [g(r)] was calculated on the basis of the following formula

$$g_{i}\left(r\right)=\frac{\rho_{i}\left(r\right)}{\rho_{i,\text{total}}}$$ (6)

where i is the selected atoms (oxygen atoms belonging to DME or FSI^−^). *r* is the distance from the reference atom Li^+^. $\rho_{i}\left(r\right)$ and $\rho_{i,\text{total}}$ represent the local density of selected atoms [$dN_{i}\left(r\right)/4\pi r^{2}\mathrm{dr}$] and the corresponding bulk density ($N_{i,\text{total}}/V$), respectively. $N_{i}\left(r\right)$ is the average number of selected atoms in the shell between *r* and r+dr. $N_{i,\text{total}}$ is the total number of selected atoms in the system. $N_{i,\text{total}}$ is the volume of the system.

Diffusion coefficient calculation from MD trajectories was carried out using the Einstein mean-squared displacement (MSD) formalism with MDAnalysis. Unwrapped coordinates were used to remove discontinuities under periodic boundary conditions. For a selected species (e.g., Li^+^), the MSD was computed as a time origin–averaged quantity

$$\text{MSD}\left(∆t\right)=\frac{1}{N}\sum_{i=1}^{N}\langle {|r_{i}\left(∆t\right)-r_{i}\left(0\right)|}^{2}\rangle$$ (7)

and the diffusion coefficient D was obtained from the Einstein relation

$$D=\underset{t\to \infty }{\text{lim}}\left\{\frac{1}{2\text{dt}}\left[\frac{1}{N}\sum_{i=1}^{N}\langle r_{i}{\left(t\right)}^{2}\rangle \right]\right\}=\underset{t\to \infty }{\text{lim}}\left[\frac{1}{2\text{dt}}\text{MSD}\right]$$ (8)

where N is the number of particles of the selected species. $r_{i}\left(t\right)$ is the position vector of particle i at time t. ∆t is the lag time. 〈·〉 denotes averaging over time origins (and particles). d is the dimensionality of the system (three for isotropic bulk). The MSD–time curve from the NVT production trajectories was inspected to identify a linear regime.

## Supplementary Materials

### The PDF file includes:

Figs. S1 to S42 Tables S1 to S4 Legends for movies S1 and S2 References
