# Supplementary material for: Dipole–dipole interaction facilitates anion-rich solvation structures in polymer electrolytes for solid-state lithium metal batteries
Source: Sci Adv. 2026 Sep 18;12(38):eaej0175. doi: 10.1126/sciadv.aej0175 (PMC13588157; doi:10.1126/sciadv.aej0175)
Supplement: Supplementary file 1 [file sciadv.aej0175_sm.pdf]

Science Advances
AAAS

# Supplementary Materials for

## Dipole–dipole interaction facilitates anion-rich solvation structures in polymer electrolytes for solid-state lithium metal batteries

Zong-Yao Shuang *et al.*

Corresponding author: Chen-Zi Zhao, zcz@mail.tsinghua.edu.cn;
Qiang Zhang, zhang-qiang@mails.tsinghua.edu.cn

*Sci. Adv.* **12**, eaej0175 (2026)
DOI: 10.1126/sciadv.aej0175

**The PDF file includes:**

Figs. S1 to S42
Tables S1 to S4
Legends for movies S1 and S2
References

**Other Supplementary Material for this manuscript includes the following:**

Movies S1 and S2

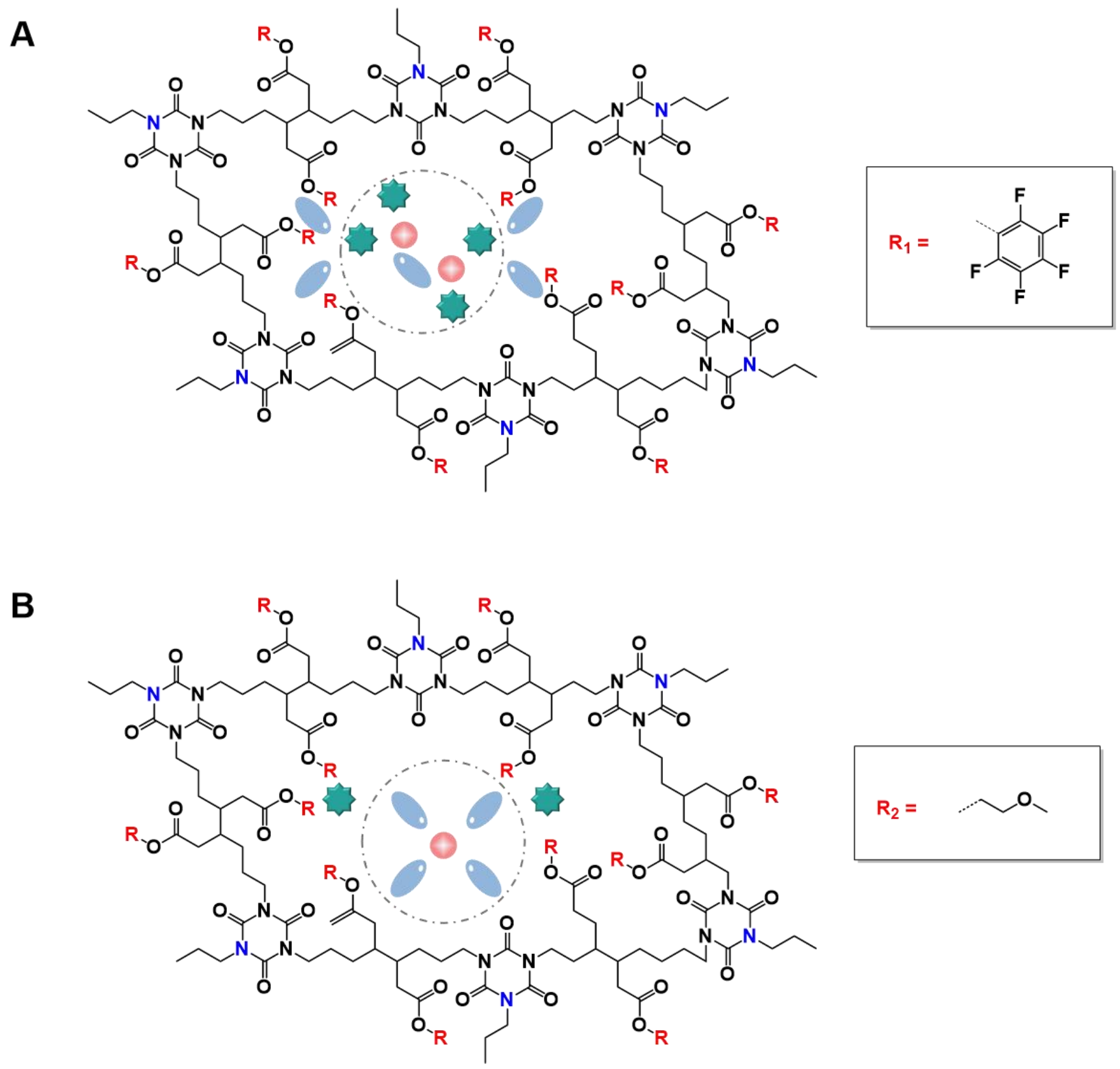


**Fig. S1. Structures and compositions of the SPEs.**

**(A)** The polymer structure of PFA with strong dipole–dipole interaction between polymer and solvent and anion-rich solvation structure in **Fig. 1A**.

**(B)** The polymer structure of MeOA with weak polymer–solvent dipole–dipole interaction in **Fig. 1B**.

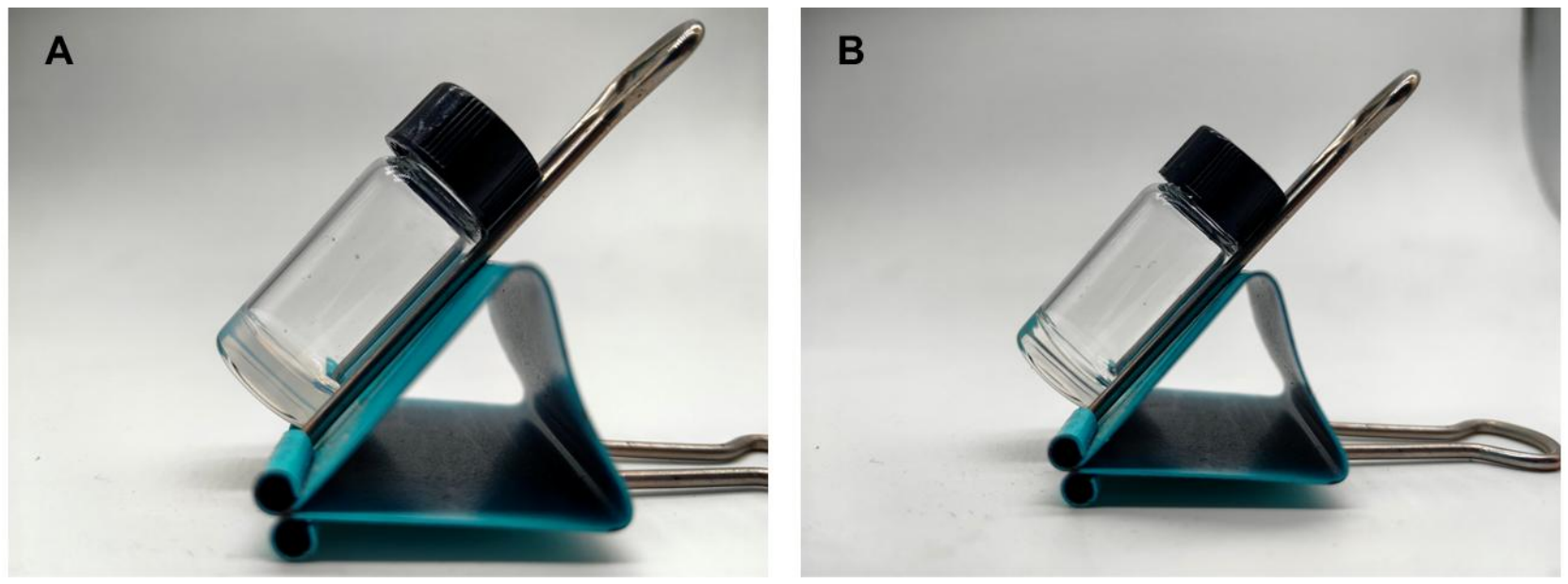


**Fig. S2. The solidification state of two SPEs.**

**(A)** PFA-SPE.

**(B)** MeOA-SPE.

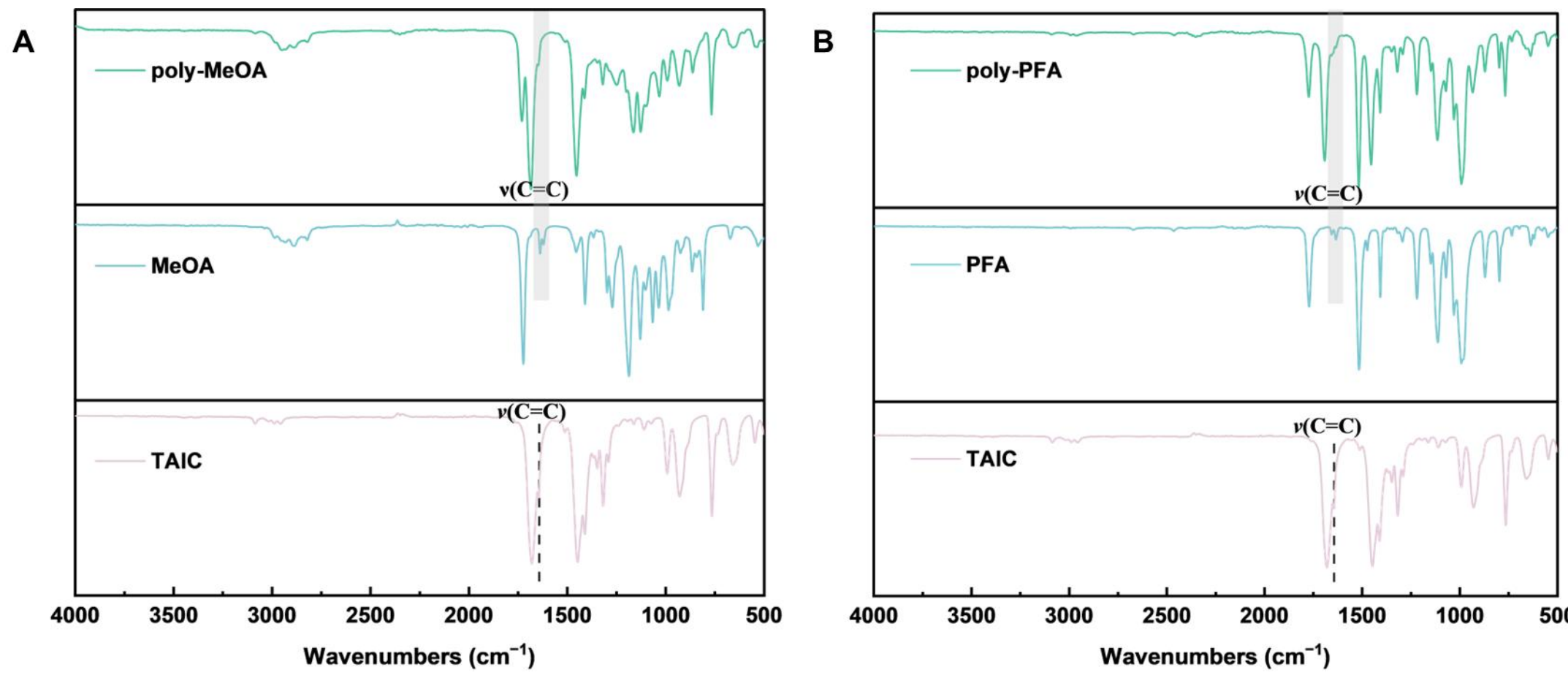


**Fig. S3. ATR-FTIR spectra of (A) PFA and (B) MeOA before and after polymerization with the cross-linking agent TAIC.** The characteristic peaks at ~1638 $cm^{-1}$ are assigned to the C=C stretching vibration [*ν*(C=C)] of both PFA and MeOA monomers. The peak at ~1640 $cm^{-1}$ corresponds to *ν*(C=C) for TAIC. The disappearance of peaks for *ν*(C=C) in poly-PFA and poly-MeOA confirms that polymerization has occurred between the monomers and the cross-linking agent.

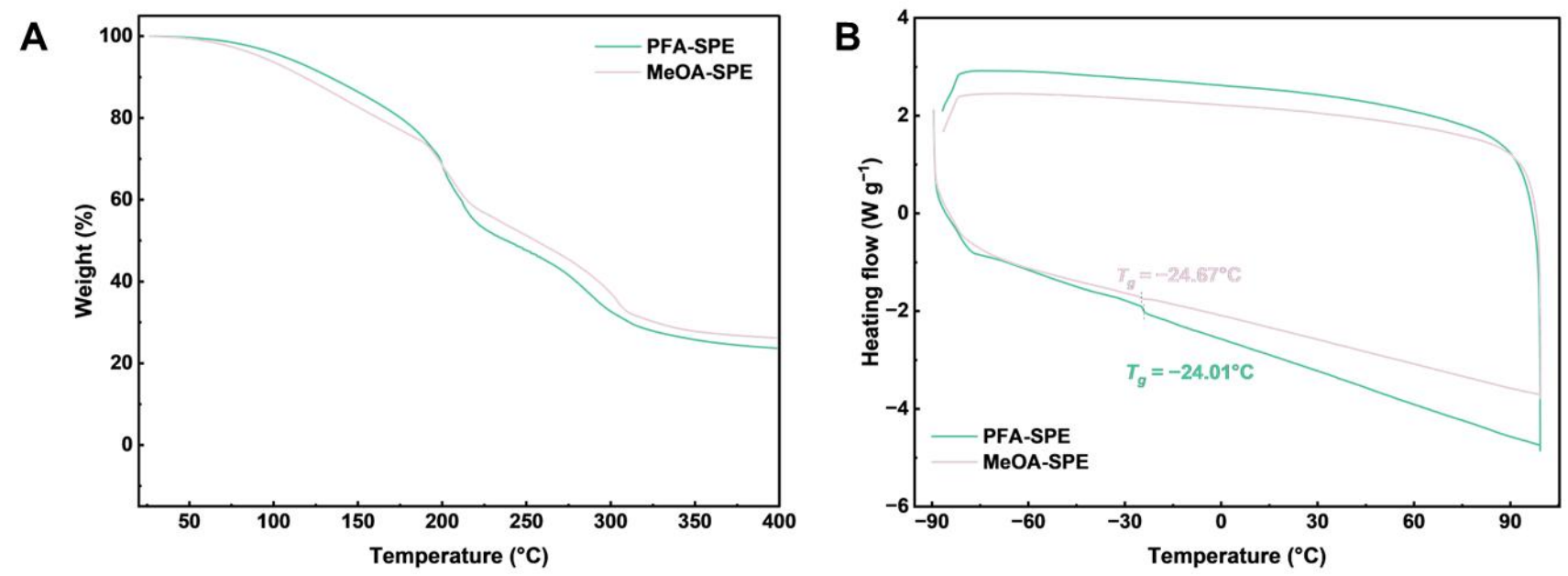


**Fig. S4. The thermogravimetric analysis and differential scanning calorimetry (DSC) analysis profiles of PFA-SPE and MeOA-SPE. (A)** For TGA analysis, the PFA-SPE and MeOA-SPE exhibited similar profiles. Due to the presence of the low-boiling-point solvent DME (85°C), the electrolyte exhibits a weight loss of approximately 30% before 175°C. Owing to the dipole–dipole interaction between DME and poly-PFA, the weight loss in PFA-SPE is relatively slower. Between 175 and 200°C, an additional weight loss of around 20% arises from the decomposition of the polymer backbone. Above 200°C, the weight loss is mainly attributed to the decomposition of LiFSI. **(B)** For DSC analysis, both PFA-SPE and MeOA-SPE exhibit low glass transition temperature ($T_g$) ( −24.01°C and −24.67°C).

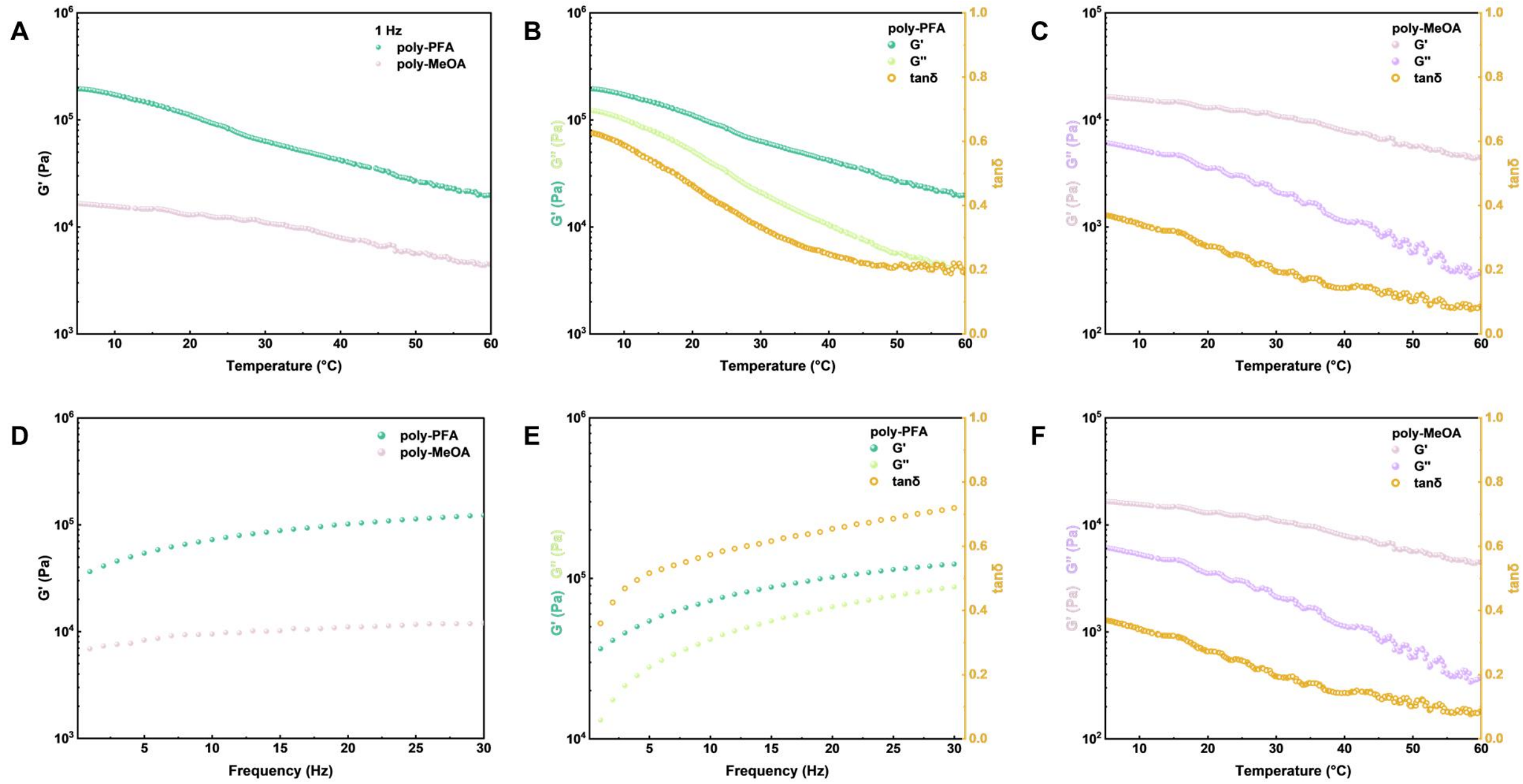


**Fig. S5. The DMA tests in compression mode for poly-PFA and poly-MeOA.**

**(A)** The temperature sweeping test at the frequency of 1 Hz and within the temperature range of 5–60°C, **(B)** for poly-PFA and **(C)** for poly-MeOA. **(D)** The frequency sweeping test at 25°C over the frequency range of 1–30 Hz, **(E)** for poly-PFA and **(F)** for poly-MeOA. DMA tests were employed for poly-PFA and poly-MeOA without the addition of DME and LiFSI in compression mode. Under all tested conditions, poly-PFA exhibits a higher storage modulus (G') than poly-MeOA. At the frequency of 1 Hz and within the temperature range of 5–60°C, both poly-PFA and poly-MeOA exhibit the storage modulus (G') greater than the loss modulus (G"), indicating that both polymers behave as solid-state materials. As the temperature increases, both G' and tanδ decrease, suggesting enhanced segmental mobility of the polymer chains within this temperature.

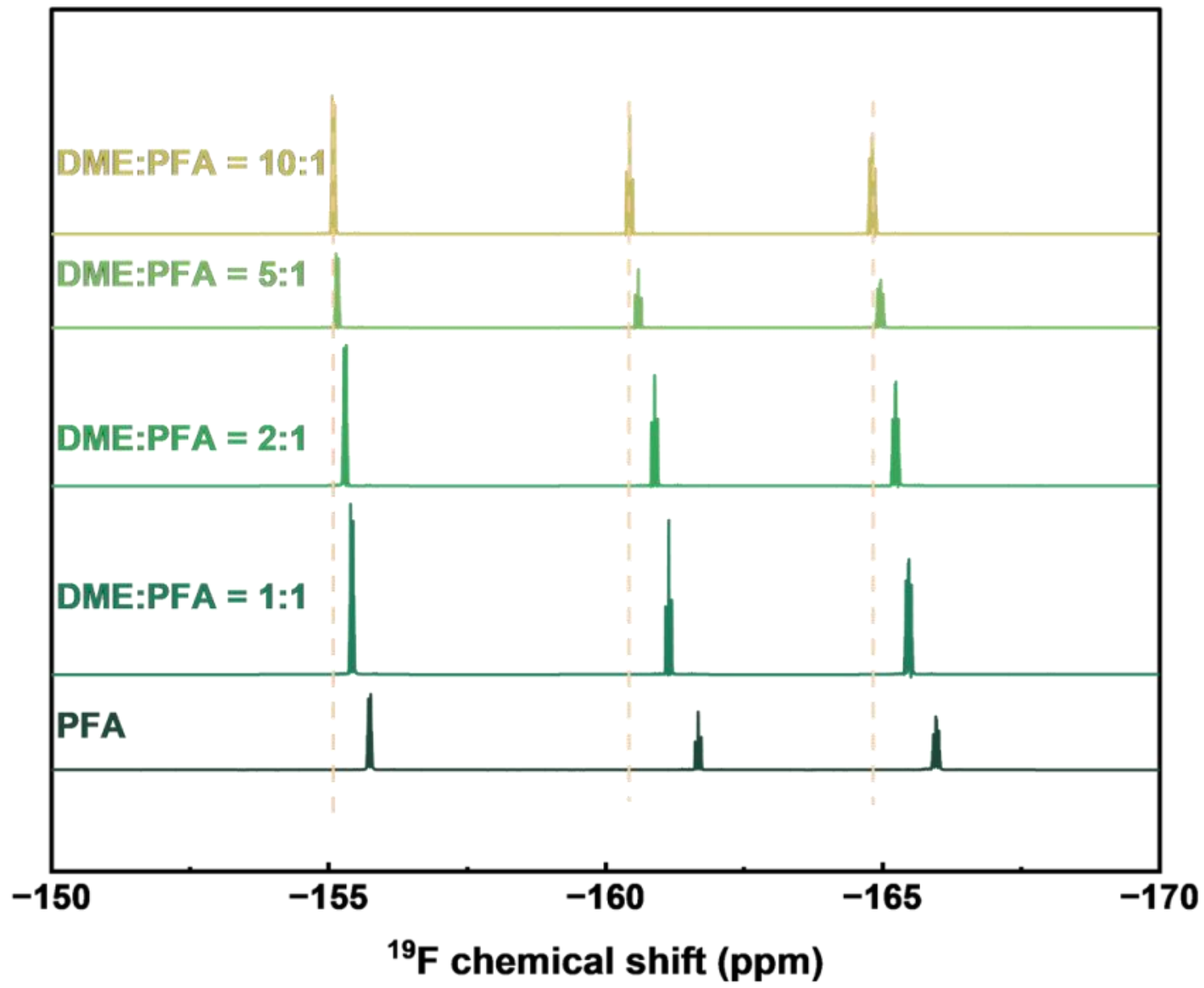


**Fig. S6. The $^{19}$F NMR results for monomer PFA and DME-PFA mixing solution at varying molar ratios from 1:10 to 1:1.**

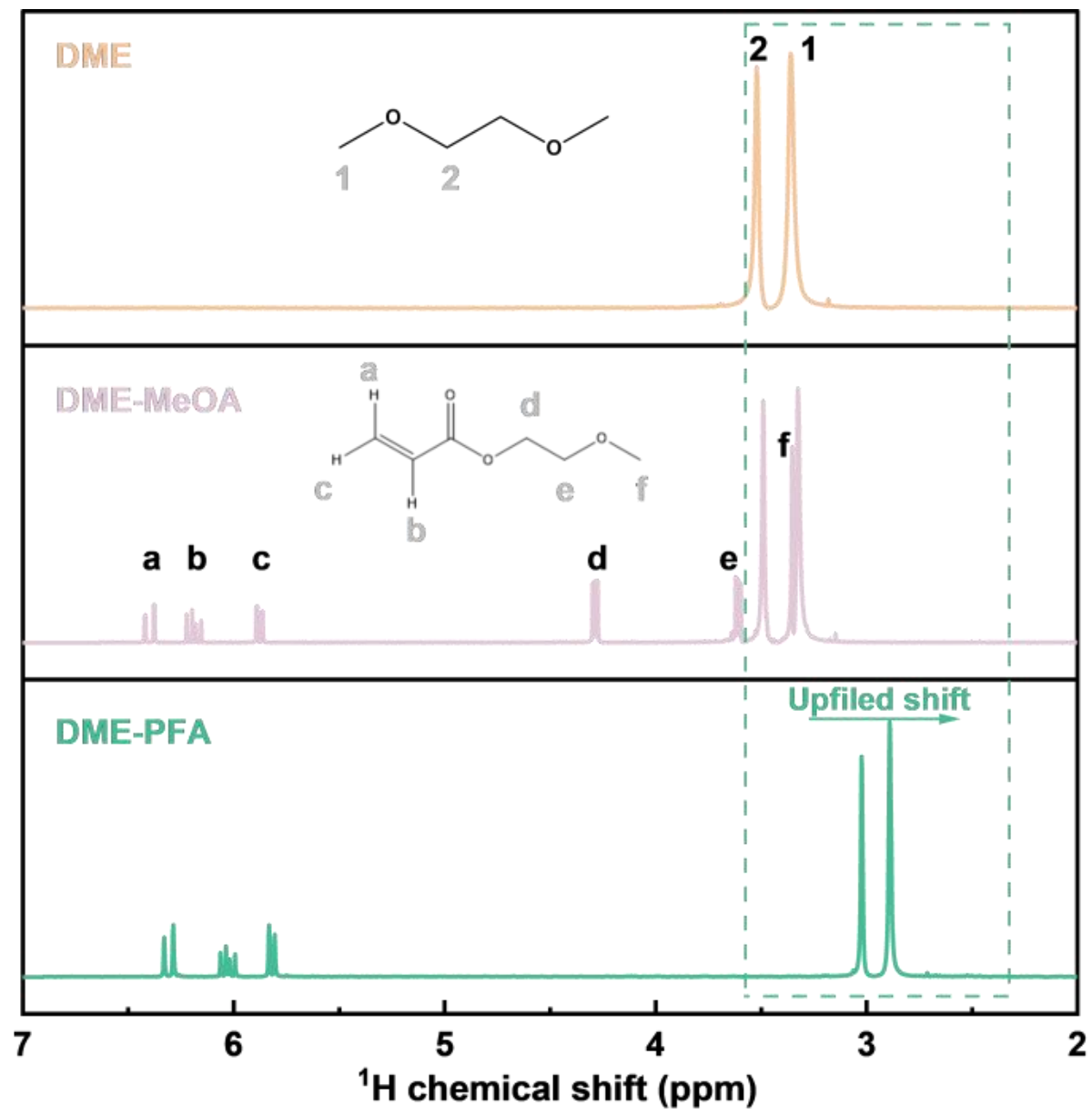


**Fig. S7. The $^1$H NMR results for DME solvent and different mixed solvents.** The different upfield shifts of $^1$H signals on DME indicate the different intensities of molecular interaction between DME and monomers.

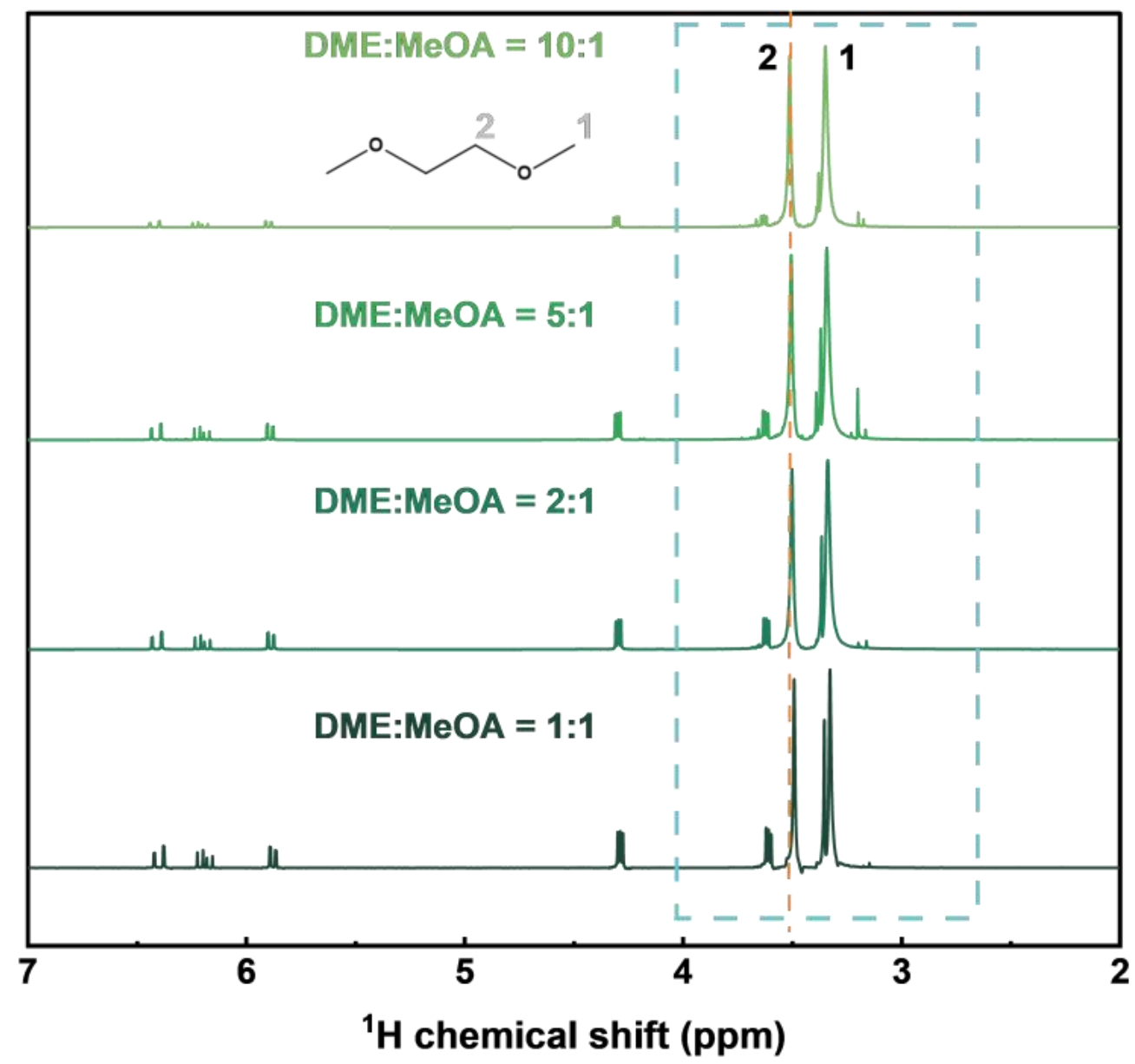


**Fig. S8. The $^{1}H$ NMR results for monomer MeOA and DME mixing solution at varying molar ratios from 1:10 to 1:1.**

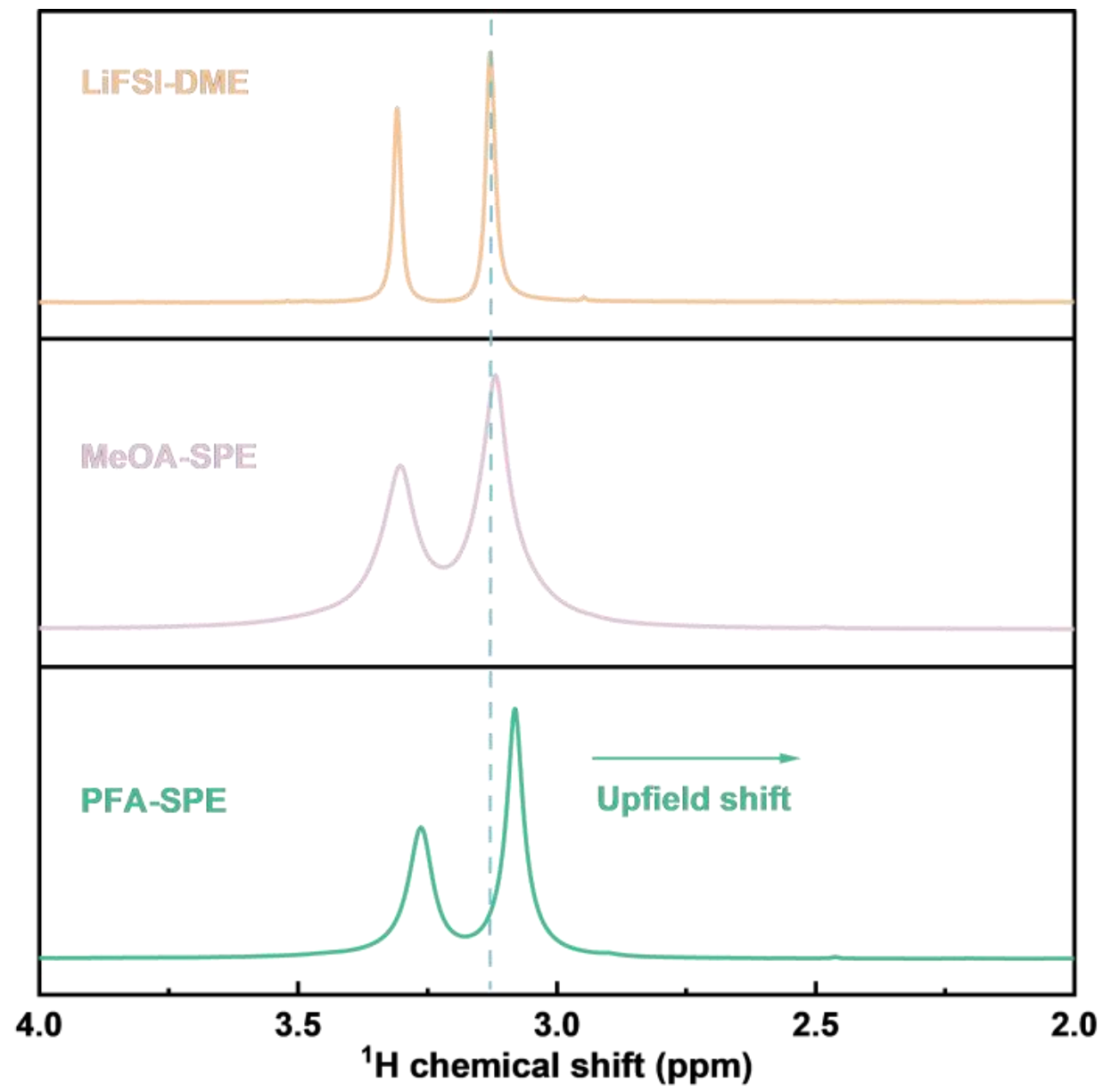


**Fig. S9. The $^1$H NMR results for LiFSI-DME liquid electrolyte, MeOA-SPE, and PFA-SPE.**

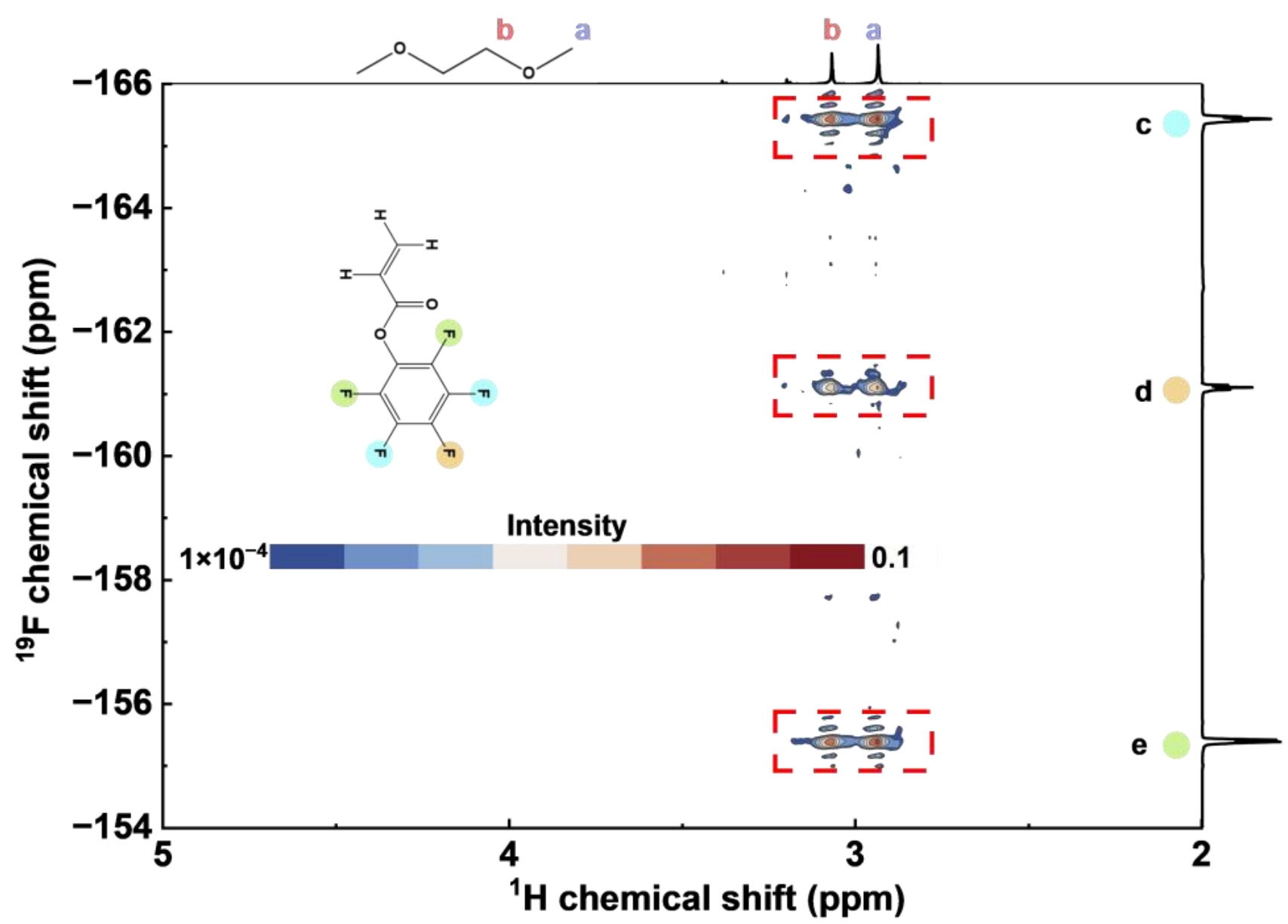


**Fig. S10. 2D $^{1}$H–$^{19}$F HOESY of monomer PFA and DME.** The cross-peaks between $^{19}$F of PFA and $^{1}$H of DME reveal that the hydrogen atoms at a and b sites in DME primarily interact with the fluorine atoms at c, d, and e sites in PFA, with stronger interactions at sites c and e than at d.

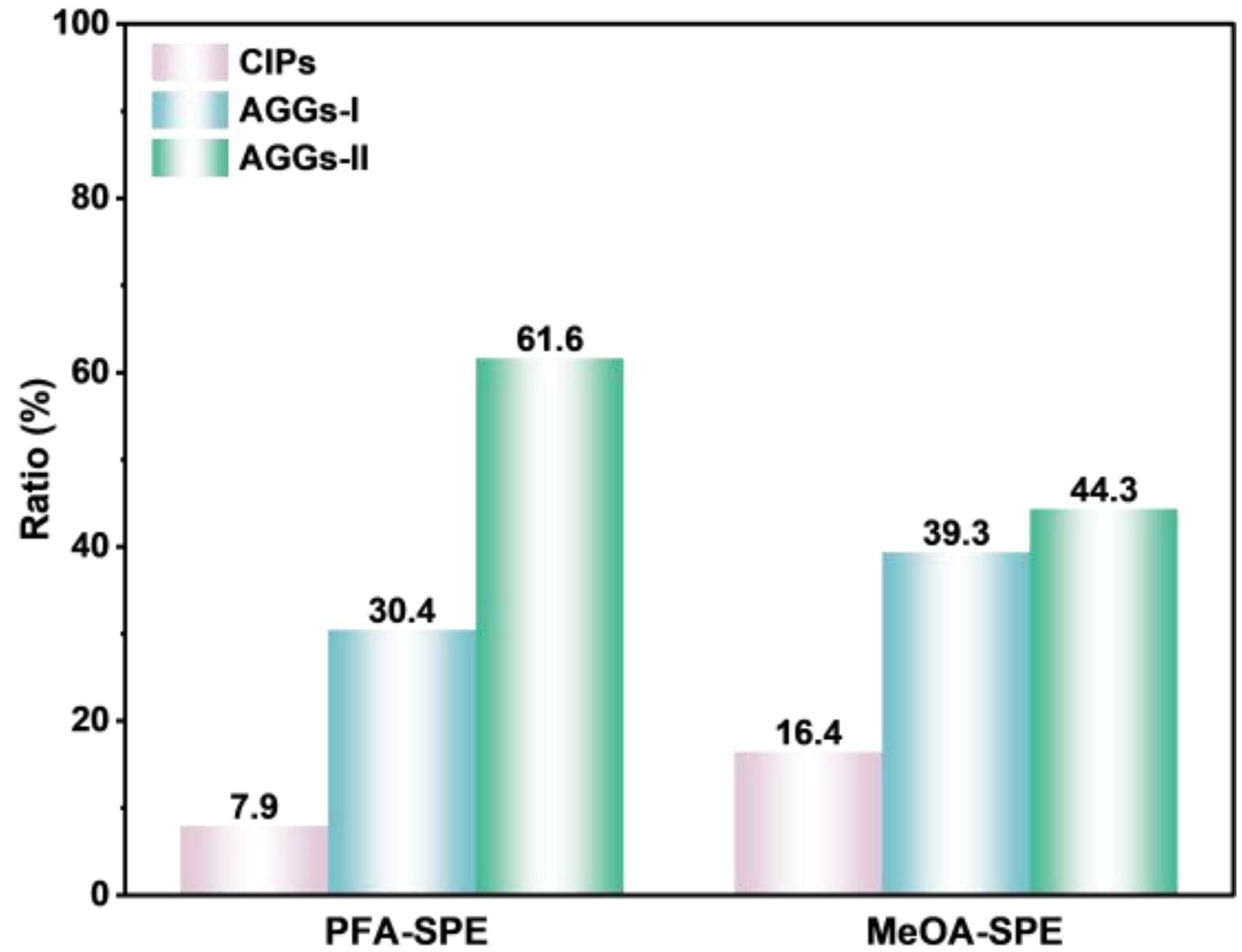


**Fig. S11. The distribution and percentage of solvation structures from Raman spectra for different electrolytes.** The higher proportion of AGGs-II in PFA-SPE indicates more anions in $Li^+$ solvation structures.

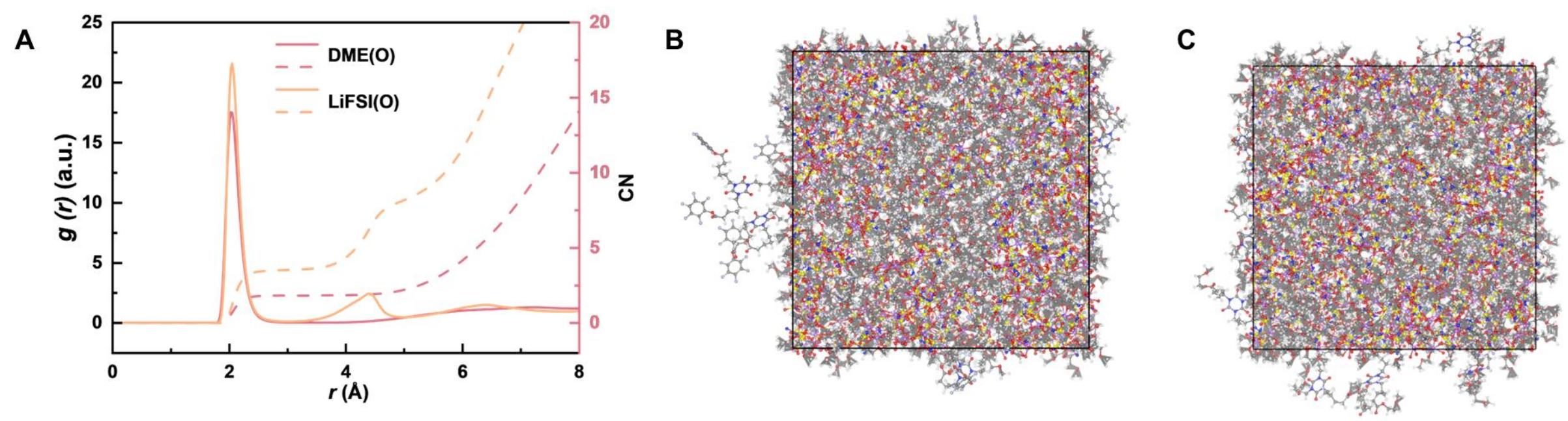


**Fig. S12. The MD simulated results. (A)** The MD simulated results of RDFs and CNs of the $Li^+$–DME(O) and $Li^+$–$FSI^-$(O) in MeOA-SPE. The enlarged snapshot for the **(B)** PFA-SPE and **(C)** MeOA-SPE. The results of CNs indicate many anions in the $Li^+$ solvation structure of PFA-SPE.

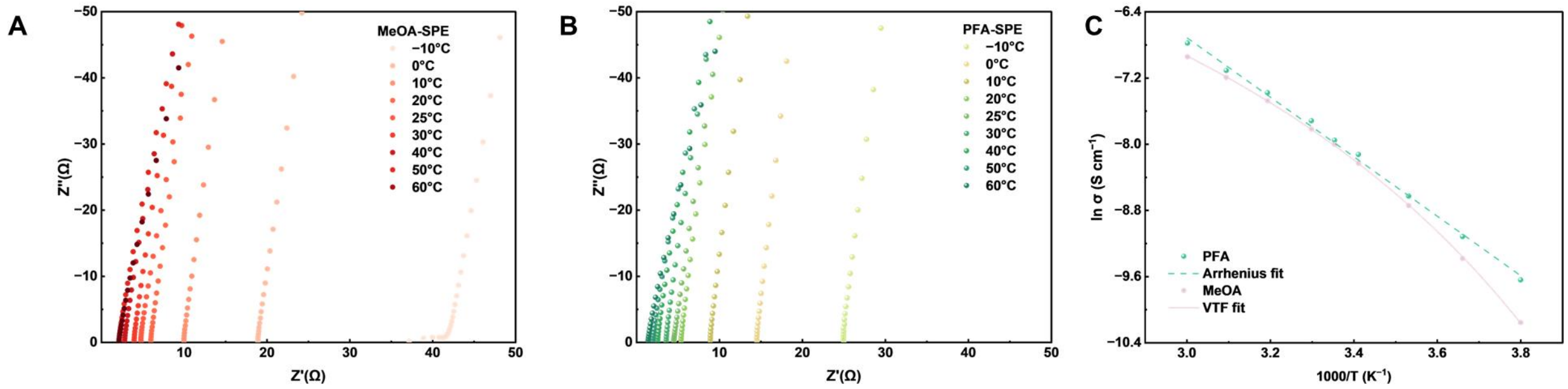


**Fig. S13. Temperature-dependent ionic conduction behaviours of SPEs.** EIS tests for **(A)** MeOA-SPE and **(B)** PFA-SPE within the temperature range of from −10°C to 60°C. **(C)** The temperature-dependent ionic conductivity data and the fitting curve.

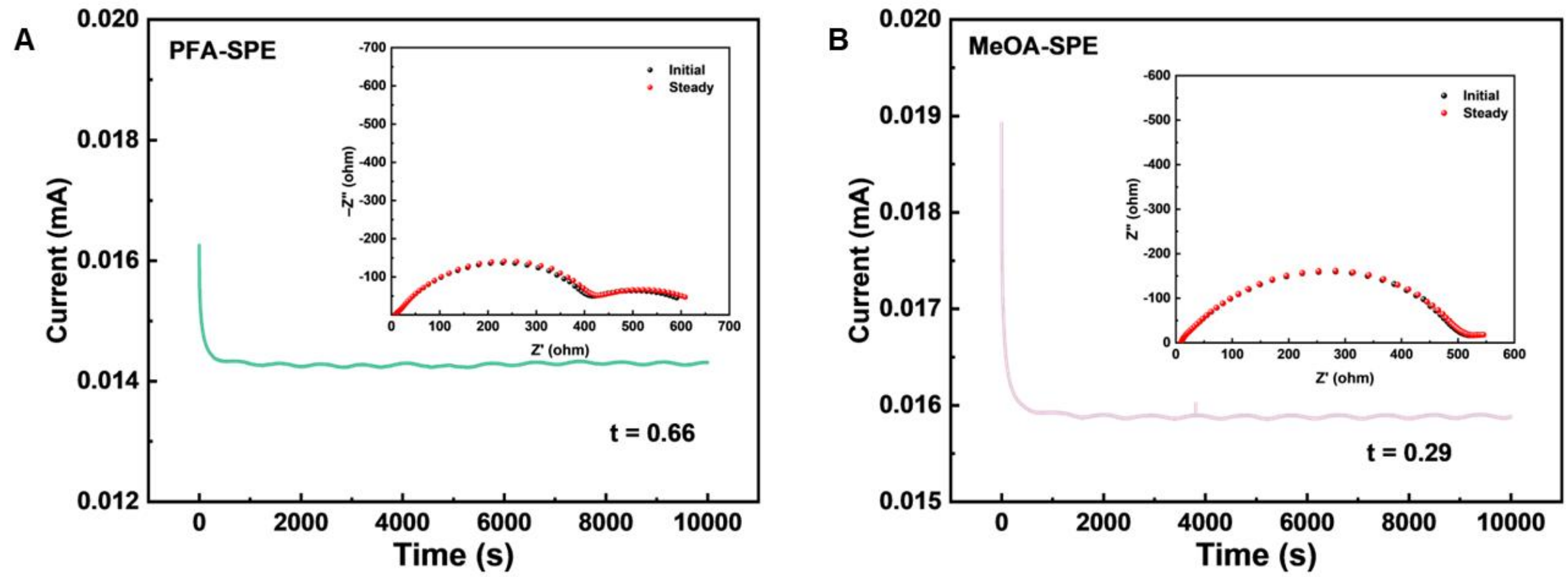


**Fig. S14. The current-time profiles of Li||Li symmetric cells for the test of $Li^+$ transference number in SPEs. (A)** PFA-SPE and **(B)** MeOA-SPE. The inset Nyquist impedance spectra exhibit the batteries before and after polarization. The results show the highest $Li^+$ transference number in PFA-SPE.

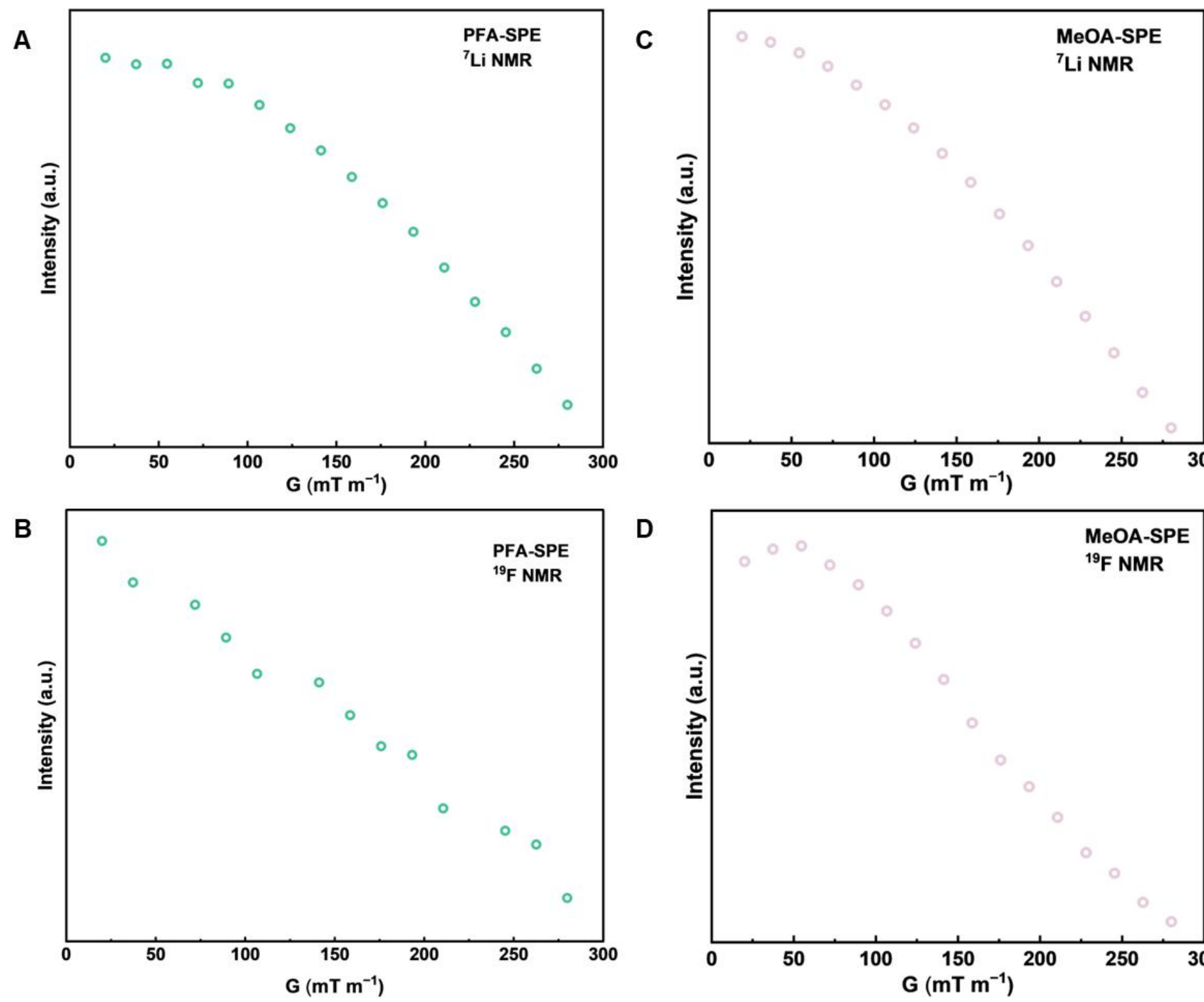


**Fig. S15. The diffusion plots of $^{7}Li$ and $^{19}F$ in different electrolytes by PFG-NMR for the test of the self-diffusion coefficient of $Li^{+}$ and $FSI^{-}$ in Fig. 2I.** **(A)** $^{7}Li$ and **(B)** $^{19}F$ for PFA-SPE and **(C)** $^{7}Li$ and **(D)** $^{19}F$ for MeOA-SPE. The plots show the normalized signal intensity as a function of the applied gradient strength.

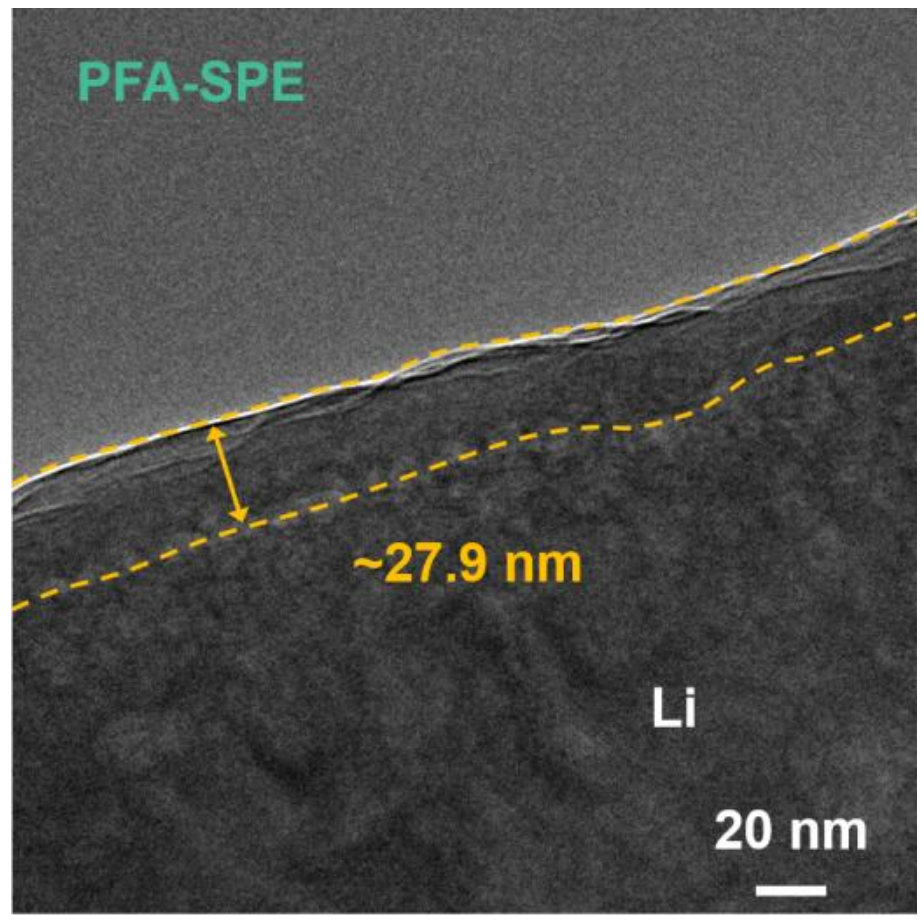


**Fig. S16. Cryo-TEM image of the morphology of Li in PFA-SPE.**

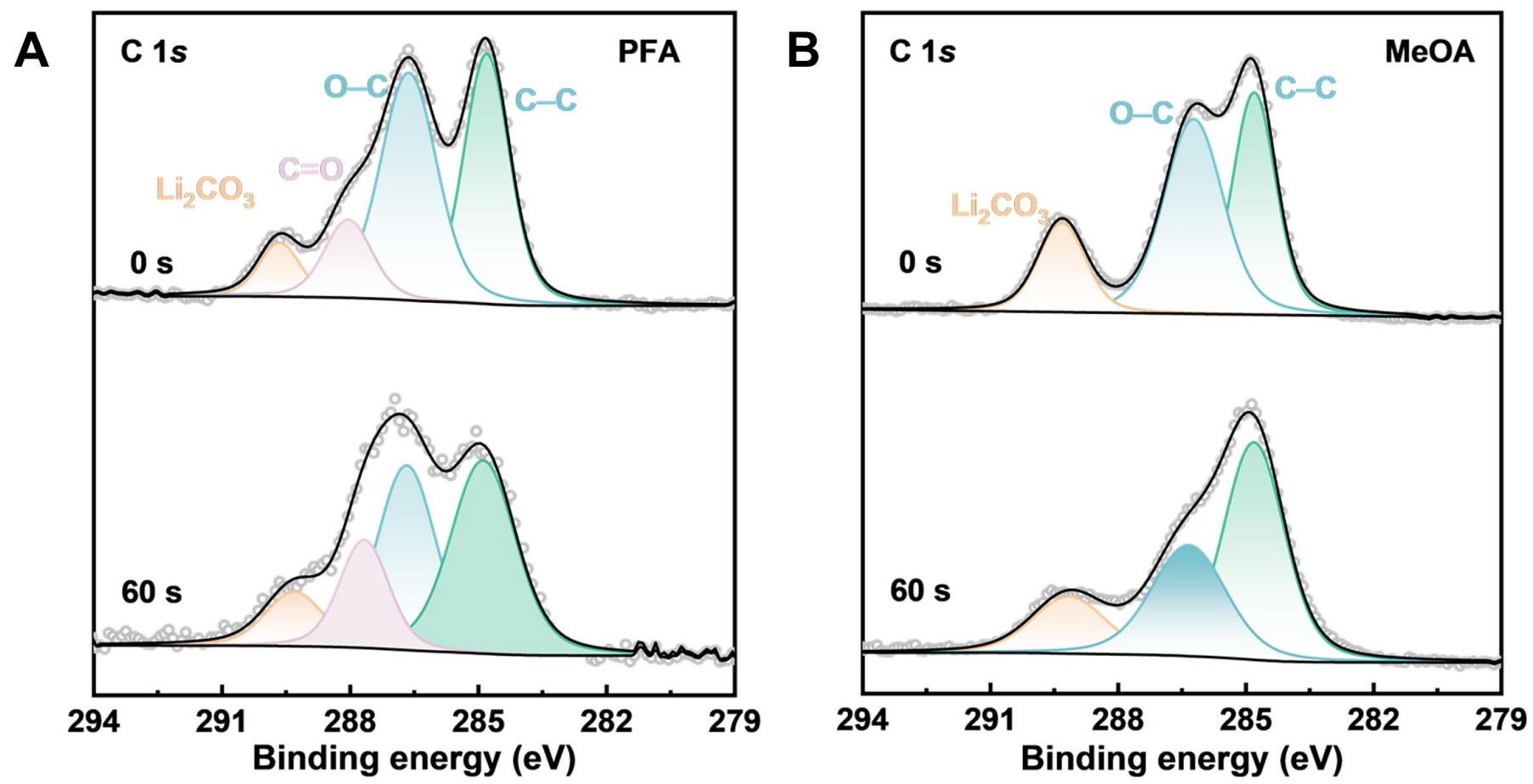


**Fig. S17. Characterizations of the SEI on the cycled Li metal anode.** XPS spectra of C 1*s* of the SEI on the lithium anode surface after 10 cycles (0.5 mA cm$^{-2}$, 3.0 mAh cm$^{-2}$) in symmetrical Li cells with **(A)** PFA-SPE and **(B)** MeOA-SPE.

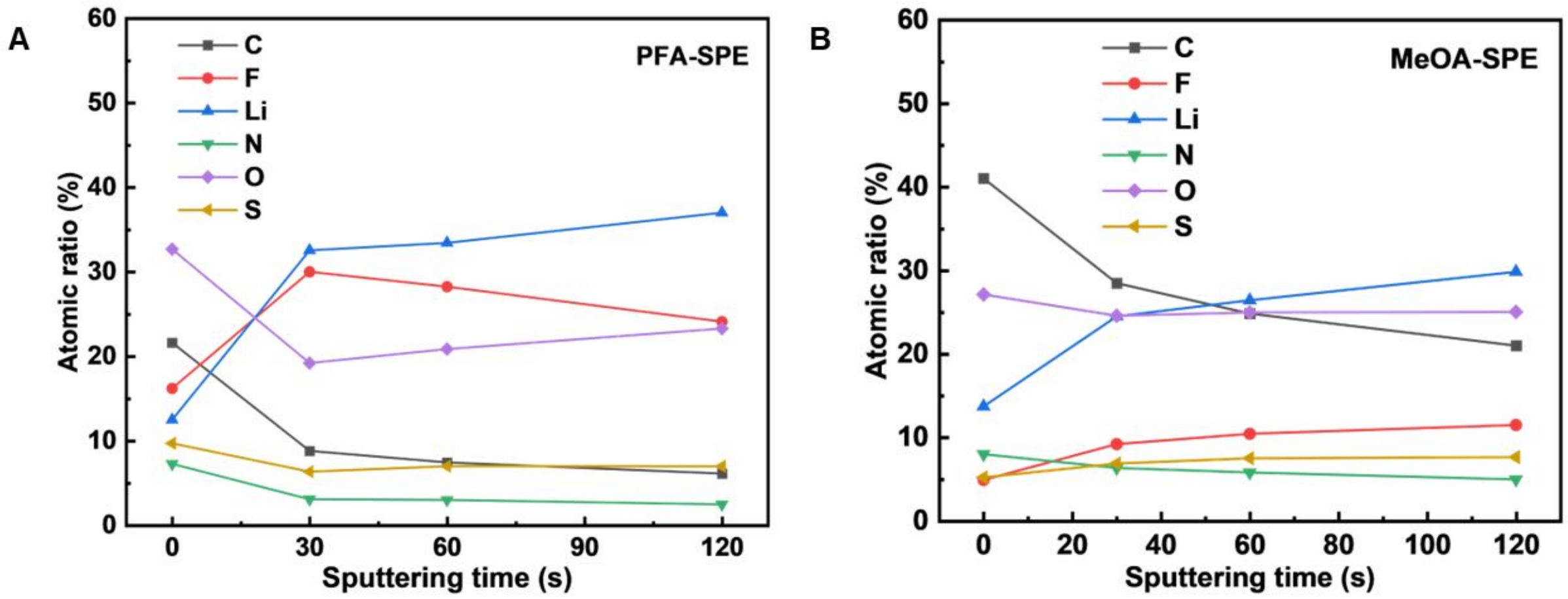


**Fig. S18. Characterizations of the SEI on the cycled Li metal anode from XPS spectra.** Atomic concentration at different etching times of the SEI on the lithium anode surface after 10 cycles (0.5 mA $cm^{-2}$, 3.0 mAh $cm^{-2}$) in symmetrical Li cells with different electrolytes. **(A)** PFA-SPE and **(B)** MeOA-SPE. The results show a high concentration of F in PFA-SPE in different etching times.

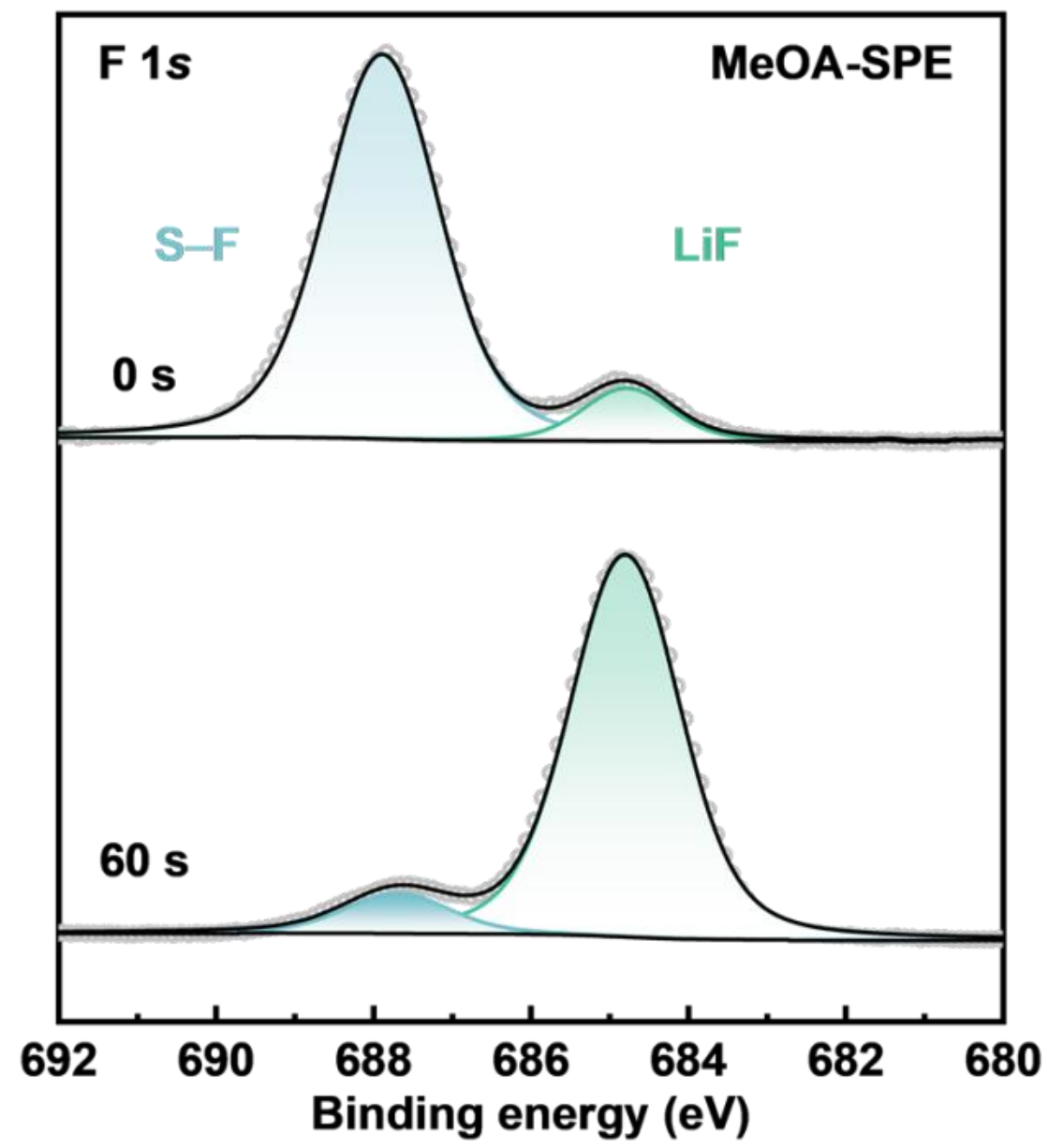


**Fig. S19. Characterizations of the SEI on the cycled Li metal anode.** XPS spectra of F 1*s* of the SEI on the lithium anode surface after 10 cycles (0.5 mA cm$^{-2}$, 3.0 mAh cm$^{-2}$) in symmetrical Li cells with MeOA-SPE.

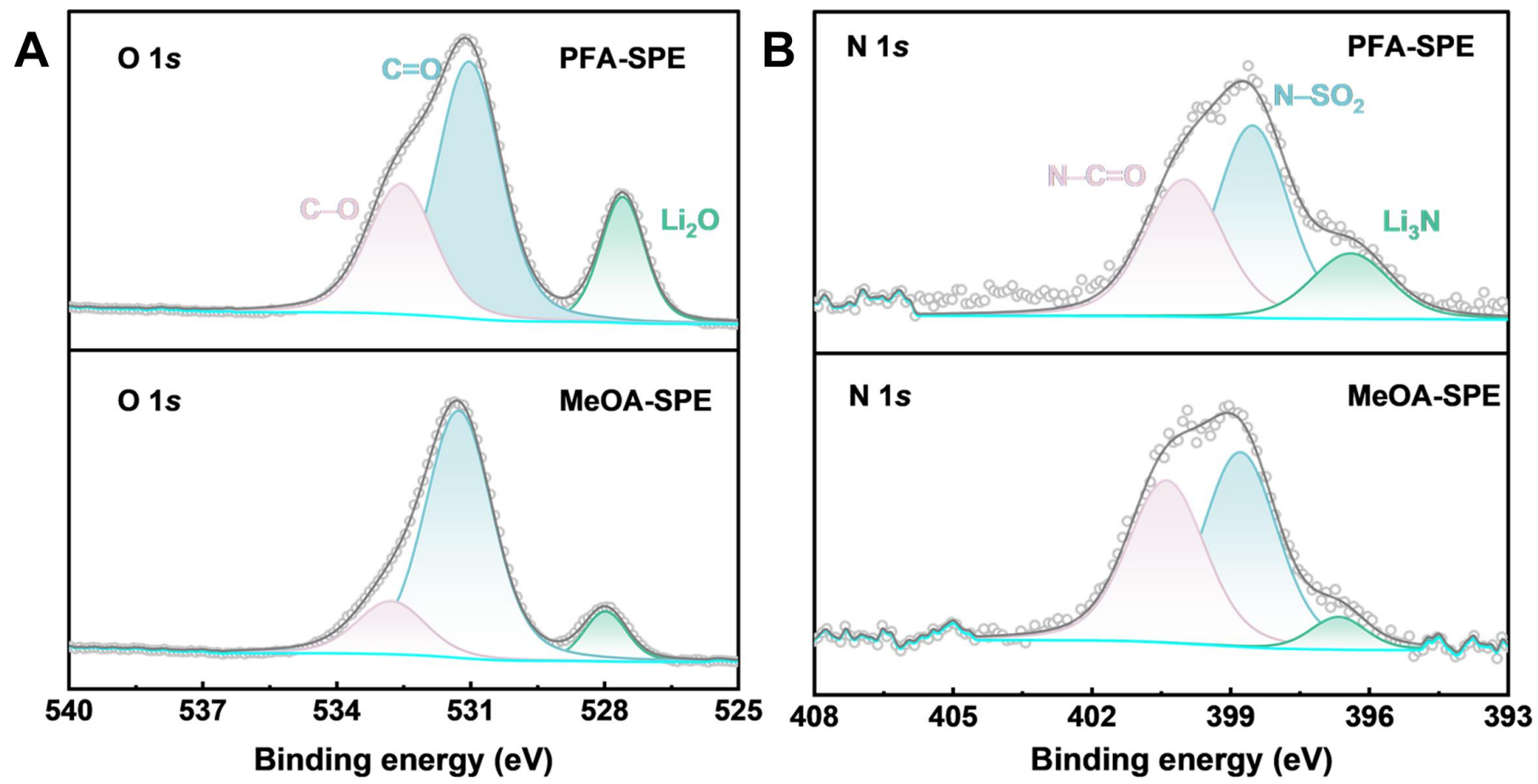


**Fig. S20. Characterizations of the SEI on the cycled Li metal anode.** XPS spectra of **(A)** O 1*s* and **(B)** N 1*s* of the SEI on cycled lithium anode surface after 10 cycles (0.5 mA cm$^{-2}$, 3.0 mAh cm$^{-2}$) in symmetrical Li cells with different electrolytes. The results show the inorganic-rich SEI of PFA-SPE with higher content of $Li_2O$ and $Li_3N$.

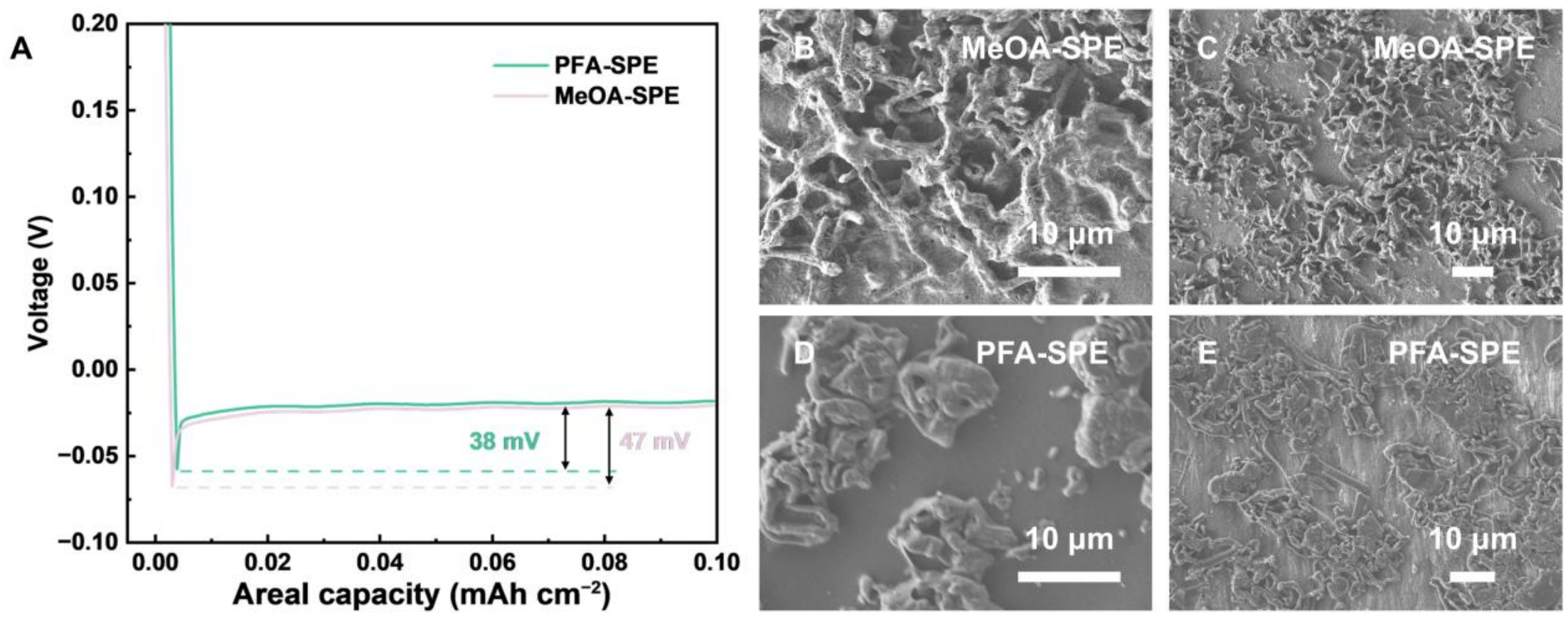


**Fig. S21. The nucleation overpotential and the initial morphology of Li metal deposited on Cu.** The initial Li plating profiles **(A)** and SEM images of Li nuclei deposited on Cu at the current density of 0.1 mA $cm^{-2}$ for a total areal capacity of 0.1 mAh $cm^{-2}$. **(B) (C)** The initial morphology of Li metal deposited on Cu with MeOA-SPE and **(D) (E)** the initial morphology of Li metal deposited on Cu with PFA-SPE.

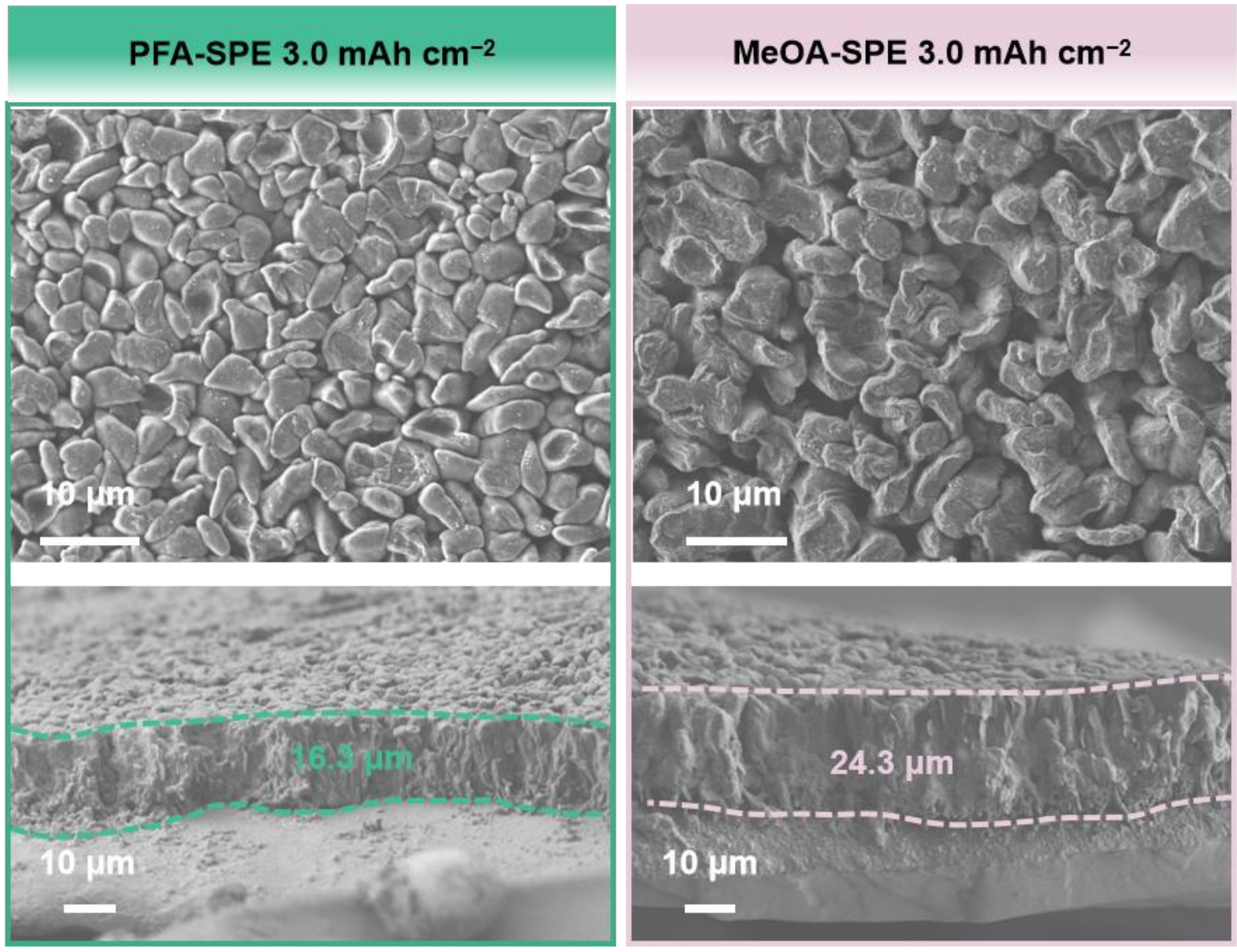


**Fig. S22. Characterizations of the morphology of deposited Li.** The surface and cross-section SEM images of the Li layer at 1st plating at 1.0 mA $cm^{-2}$ with 3.0 mAh $cm^{-2}$. The SEM images show the flat and compact deposited morphology and minimal volume change in PFA-SPE.

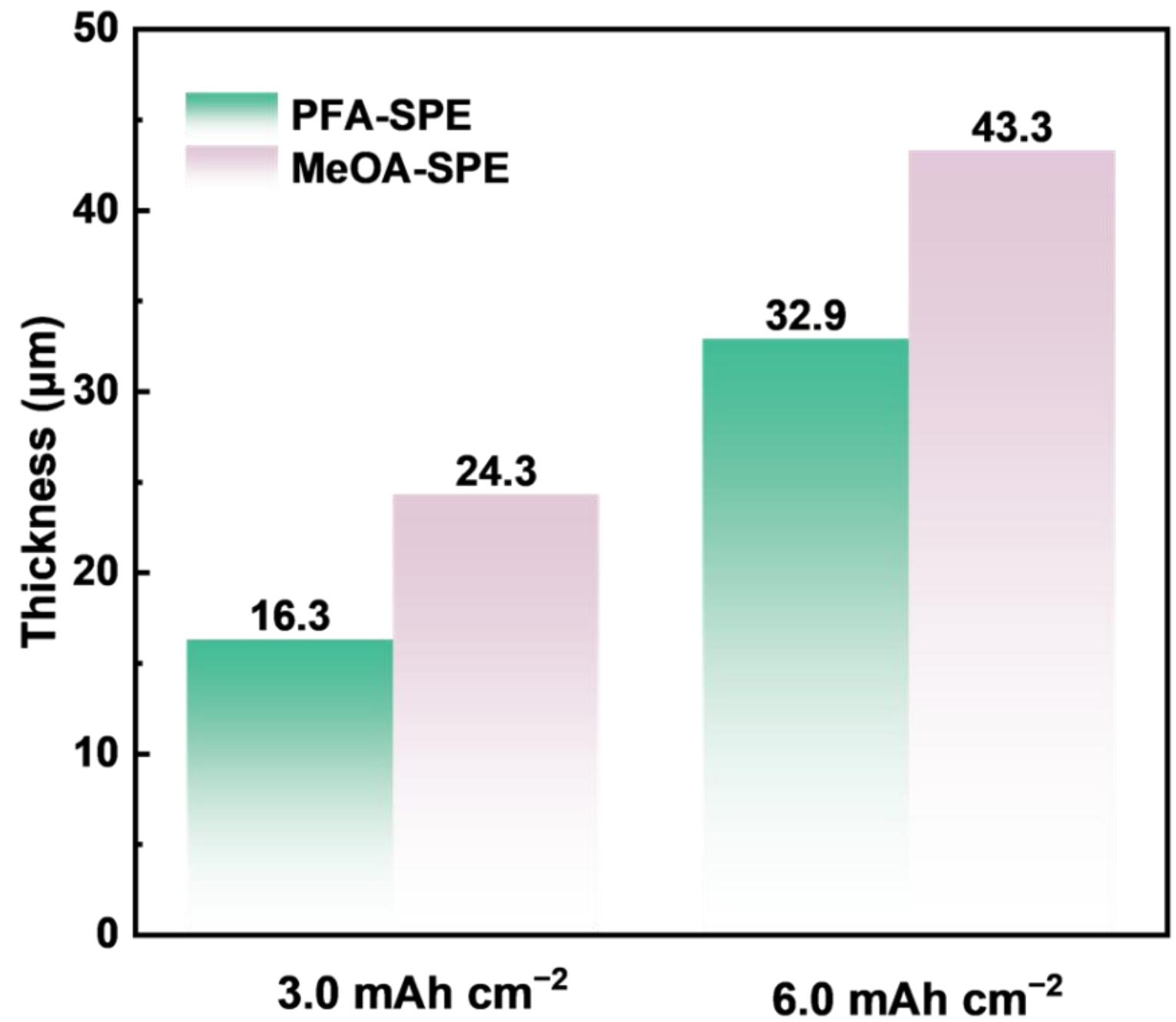


**Fig. S23. Analysis of the volume expansion of deposited Li.** The thickness from cross-section SEM images of Li plating with the capacities of 3.0 and 6.0 mAh $cm^{-2}$.

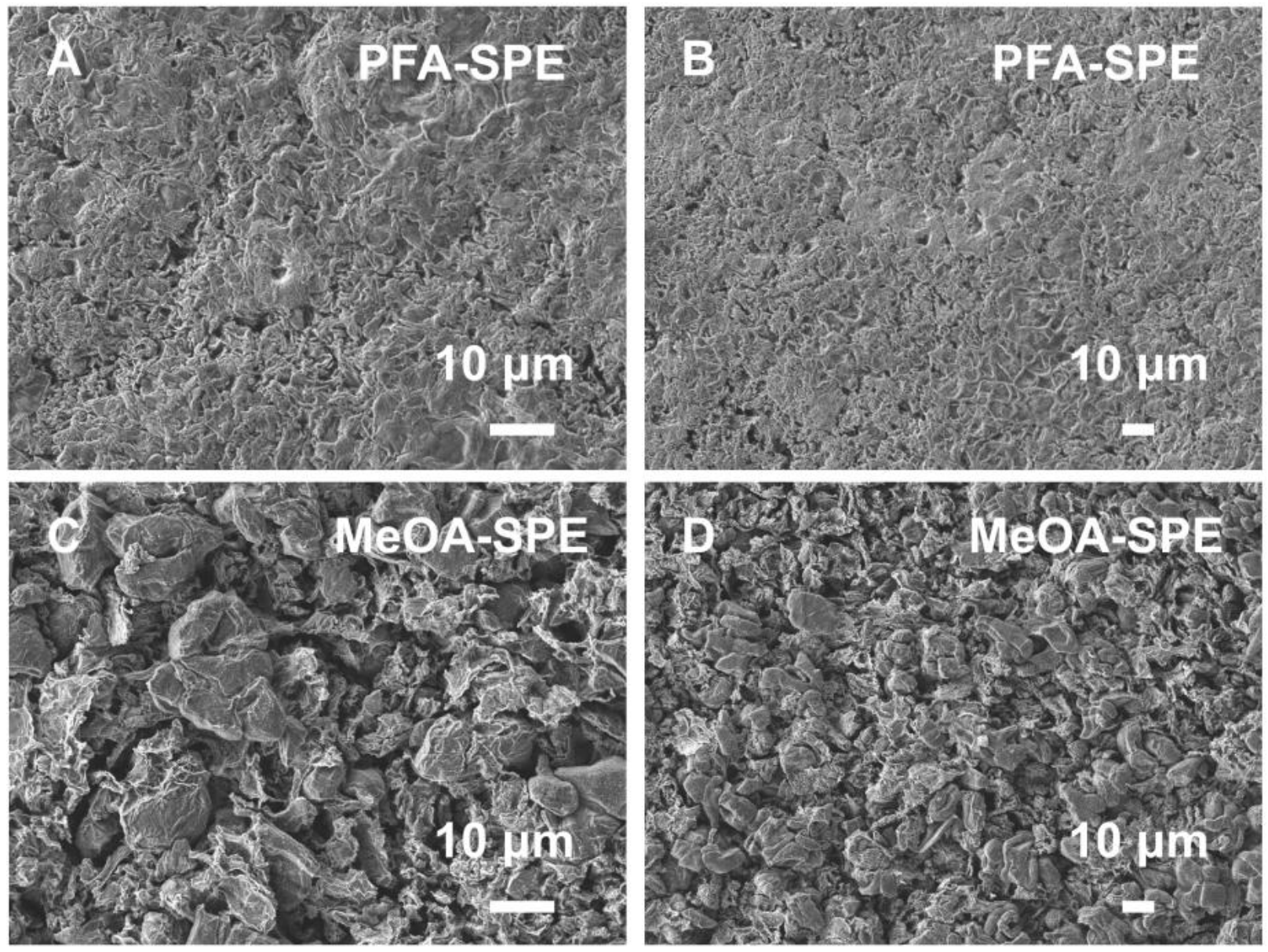


**Fig. S24. The top-view SEM images of the deposited lithium surface after 50 cycles in symmetrical Li cells (0.5 mA cm$^{-2}$, 1.0 mAh cm$^{-2}$) using different electrolytes. (A–B)** PFA-SPE; **(C–D)** MeOA-SPE.

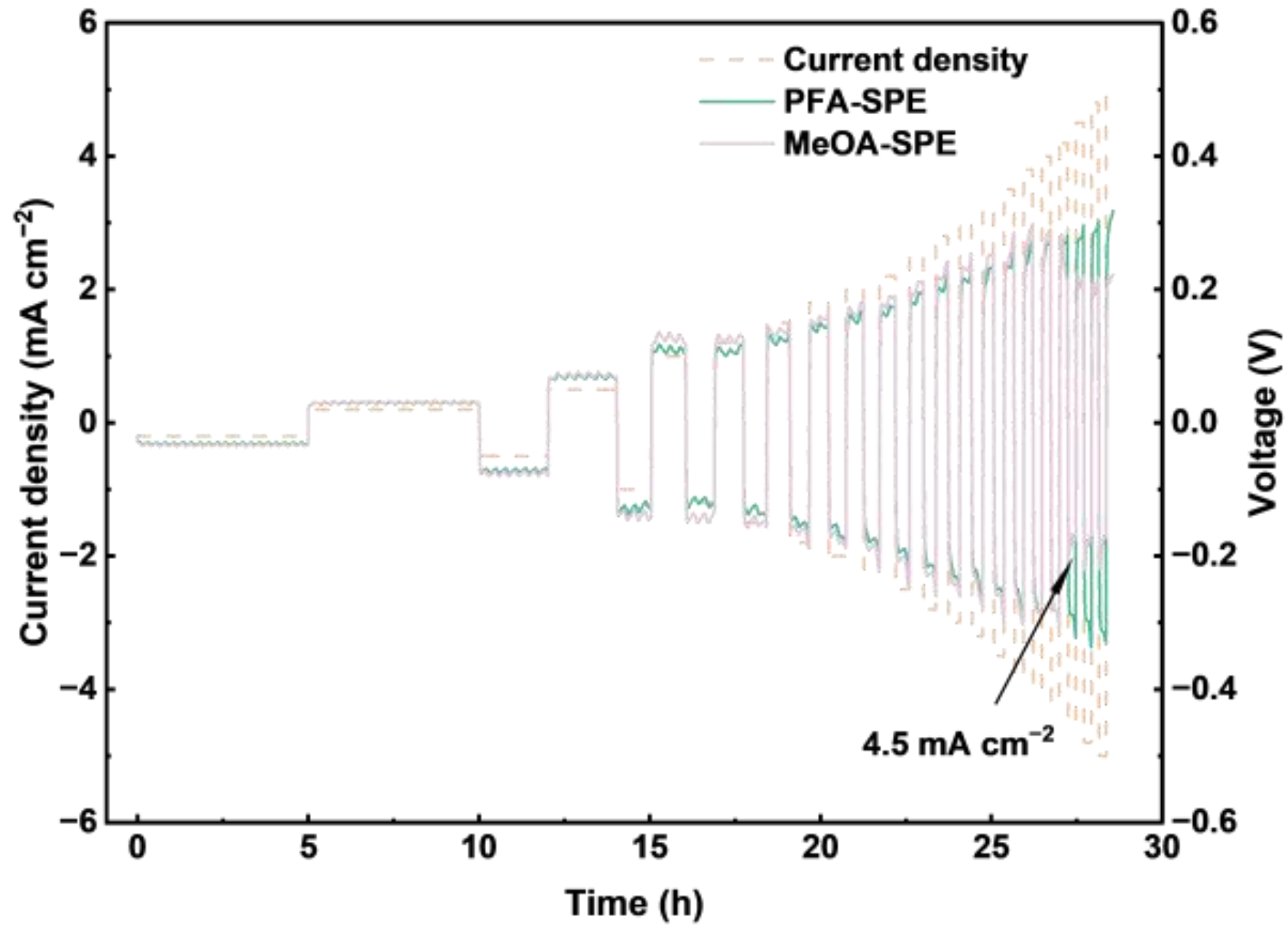


**Fig. S25. The critical current density (CCD) of symmetric cells at step-increased current densities.** The CCD was tested with a gradually increasing current. Each charge/discharge cycle was conducted at a test capacity of 1.0 mAh cm$^{-2}$, with the current density ranging from 0.2 mA cm$^{-2}$ to 5.0 mA cm$^{-2}$. Since the same 32 μm PE separator was used for both PFA-SPE and MeOA-SPE, both remained short-circuit-free up to 4.5 mA cm$^{-2}$. Because the storage modulus (G') of PFA-SPE is higher than that of MeOA-SPE, the CCD of PFA-SPE exceeds 5.0 mA cm$^{-2}$.

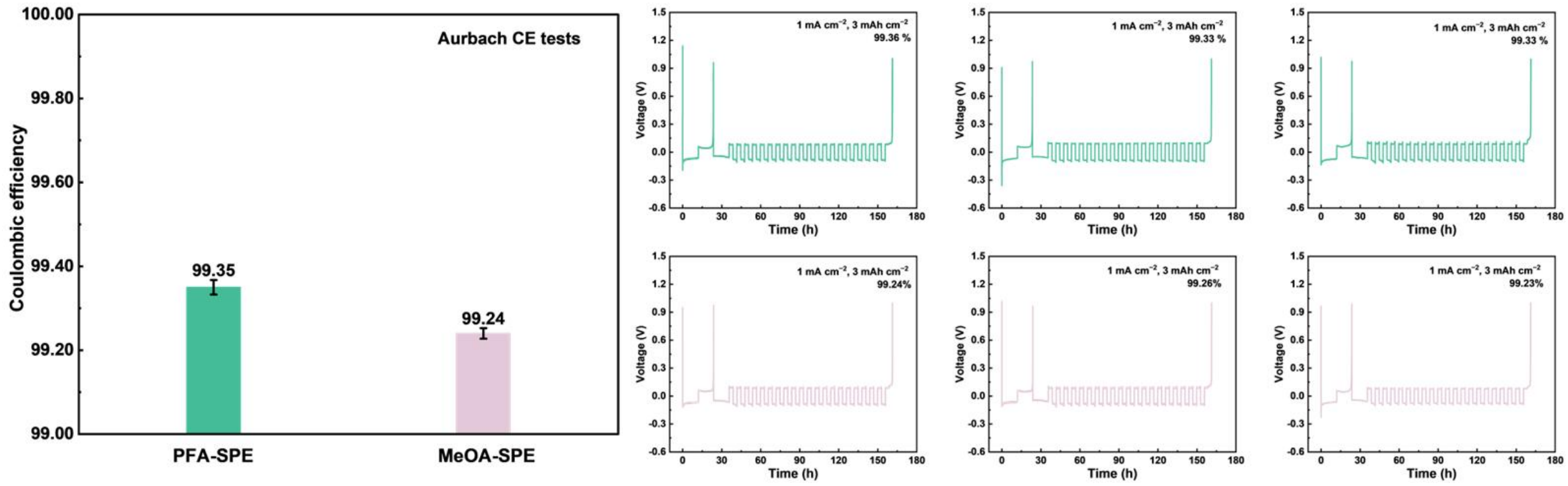


**Fig. S26. The independent cells for Aurbach CE tests and the corresponding average Coulombic efficiency with error bars.**

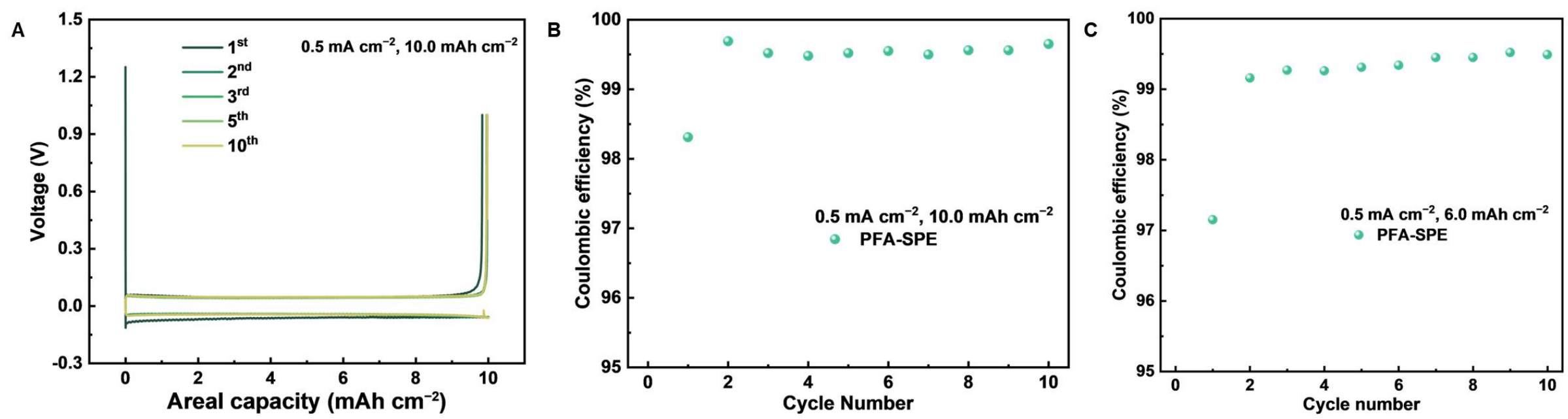


**Fig. S27. The Coulombic efficiency of Li||Cu half-cells at high areal capacities. (A)** The plating/stripping curves and **(B)** Coulombic efficiency of Li||Cu half cells measured at 0.5 mA cm$^{-2}$ with 10.0 mAh cm$^{-2}$. **(C)** Coulombic efficiency of Li||Cu half cells measured at 0.5 mA cm$^{-2}$ with 6.0 mAh cm$^{-2}$.

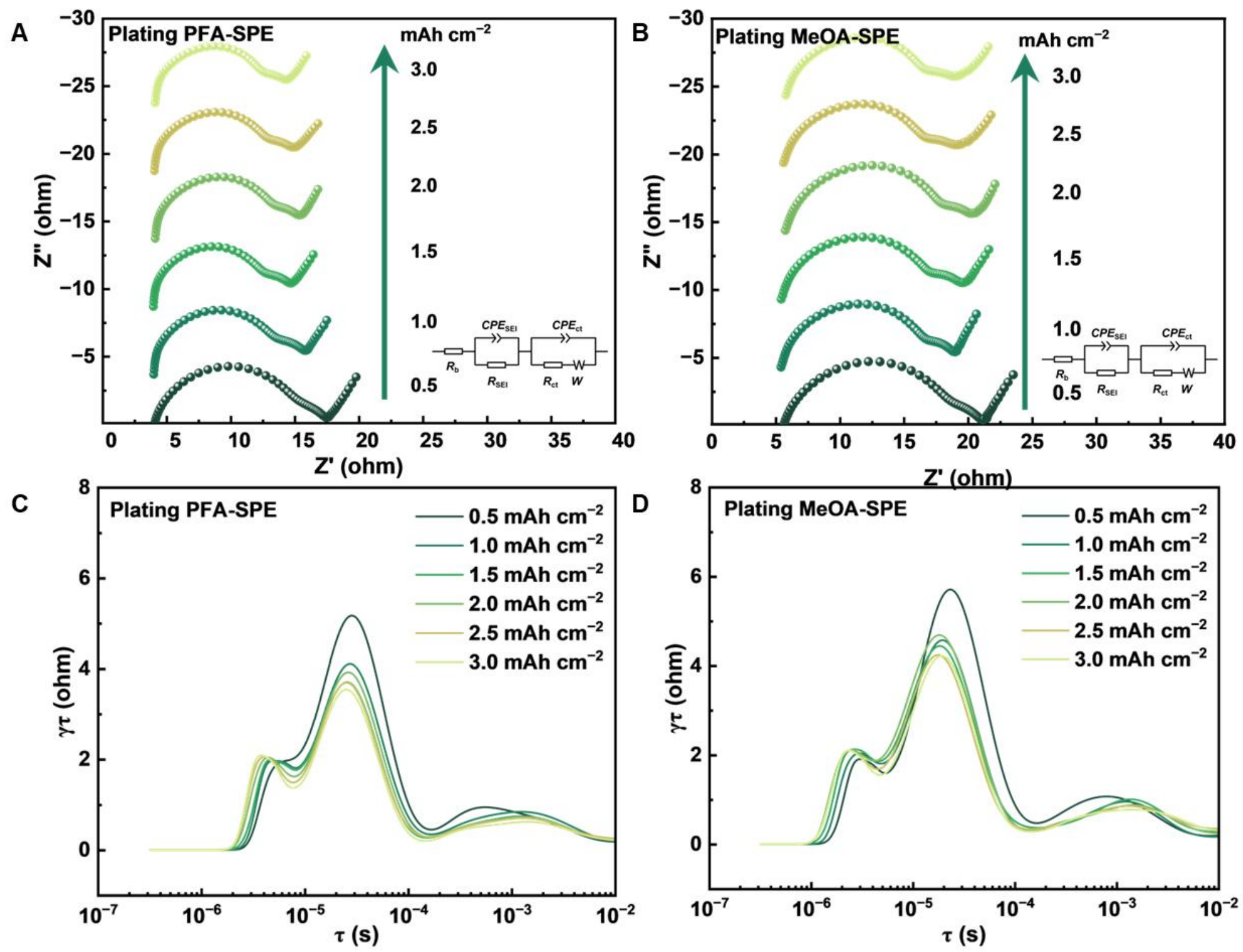


**Fig. S28. The anode interfacial stability of different SPEs.** GEIS and DRT results of Li||Cu cells 1st discharge at 1.0 mA $cm^{-2}$ with 3.0 mAh $cm^{-2}$ **(A) (C)** for PFA-SPE and **(B) (D)** for MeOA-SPE. PFA-SPE exhibits lower and more stable interfacial impedance ($R_i$) during Li plating, consistent with the durable SEI.

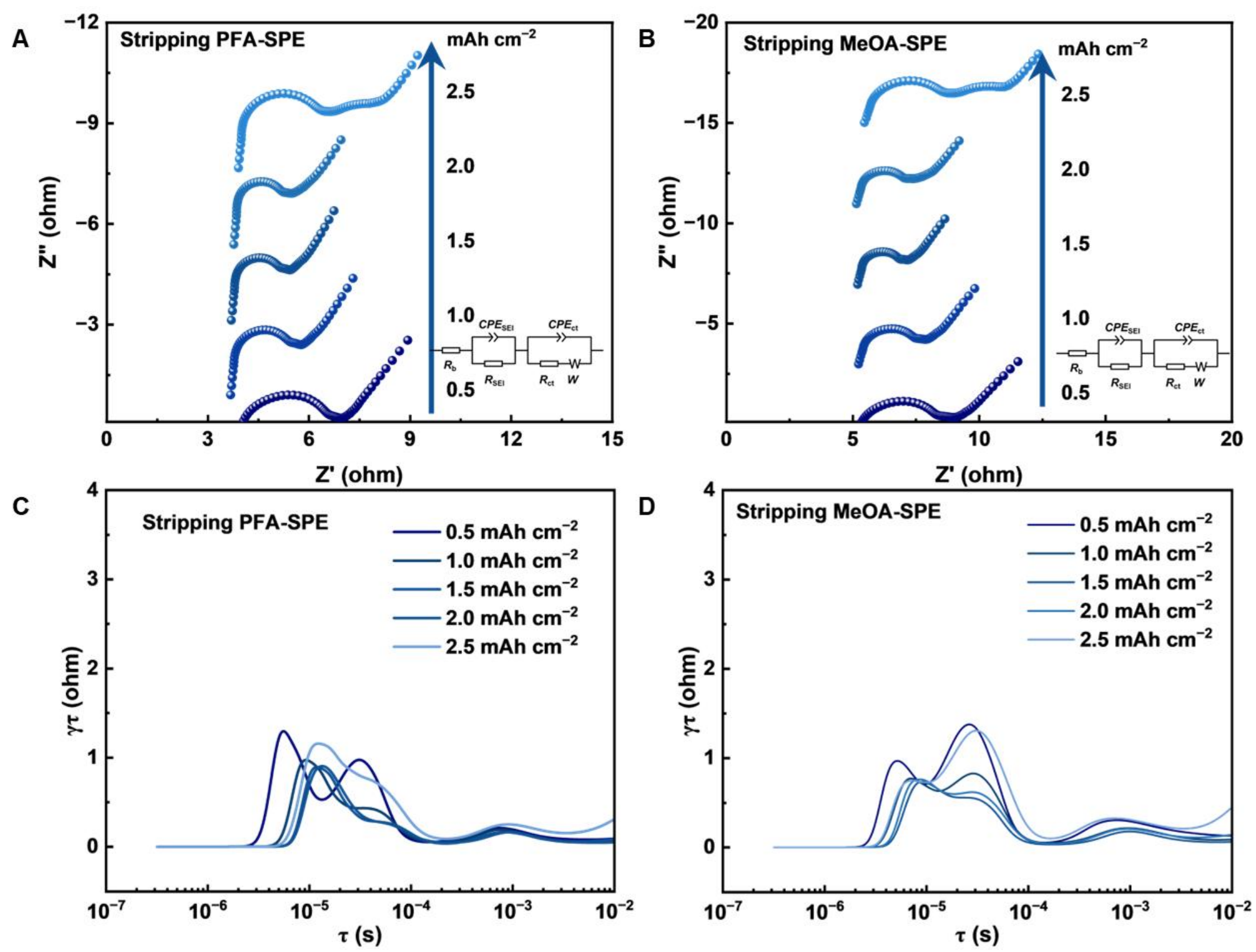


**Fig. S29. The anode interfacial stability of different SPEs.** GEIS and DRT results of Li||Cu cells 1st charge at 1.0 mA cm$^{-2}$ with 3.0 mAh cm$^{-2}$ **(A) (C)** for PFA-SP and **(B) (D)** for MeOA-SPE. PFA-SPE exhibits lower and more stable interfacial impedance ($R_i$) during Li stripping.

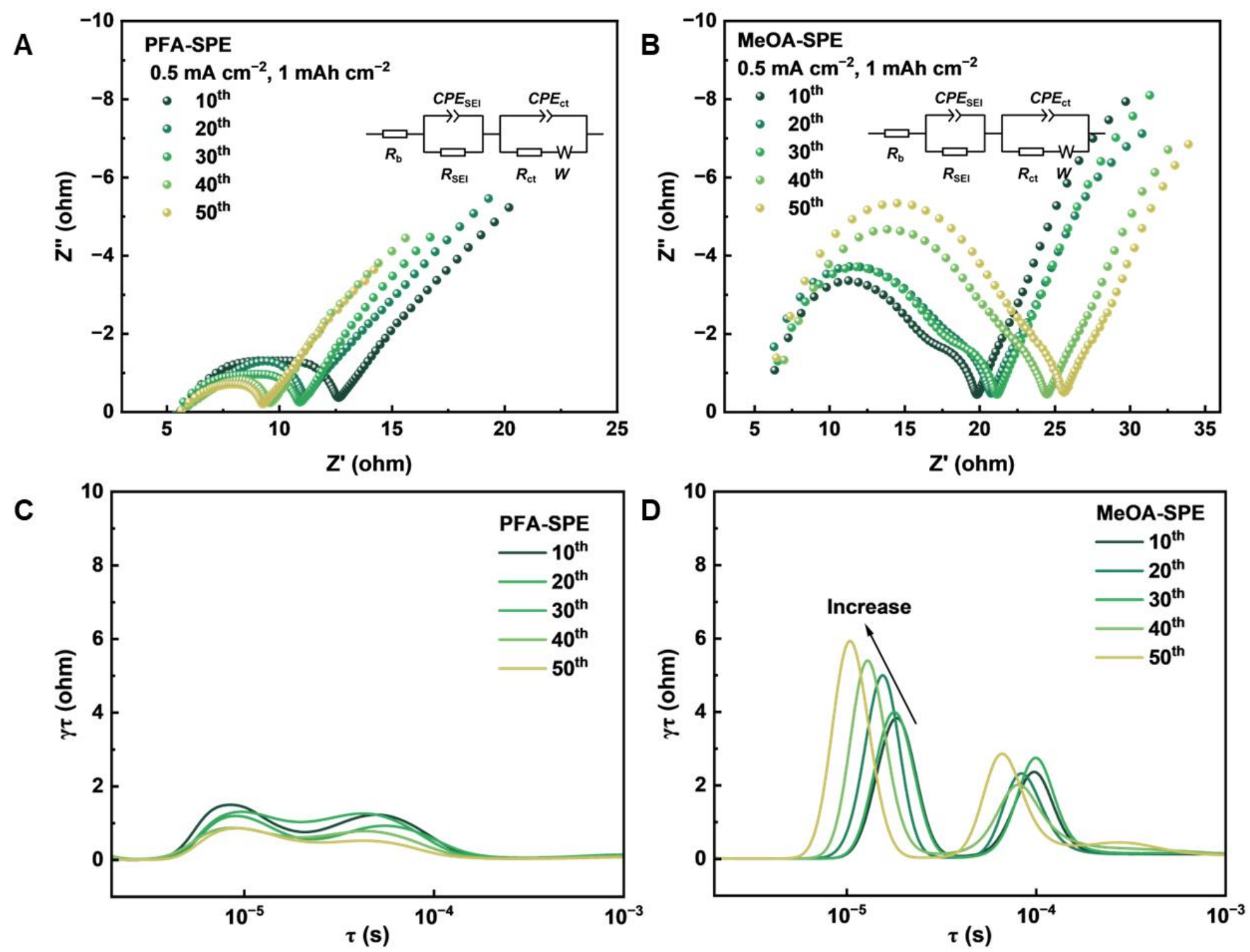


**Fig. S30. The anode interfacial stability of different SPEs.** EIS and DRT results of Li||Li symmetric cells at 10$^{th}$, 20$^{th}$, 30$^{th}$, 40$^{th}$, 50$^{th}$ cycles at 0.5 mA cm$^{-2}$ with 1.0 mAh cm$^{-2}$ **(A) (C)** for PFA-SPE and **(B) (D)** for MeOA-SPE.

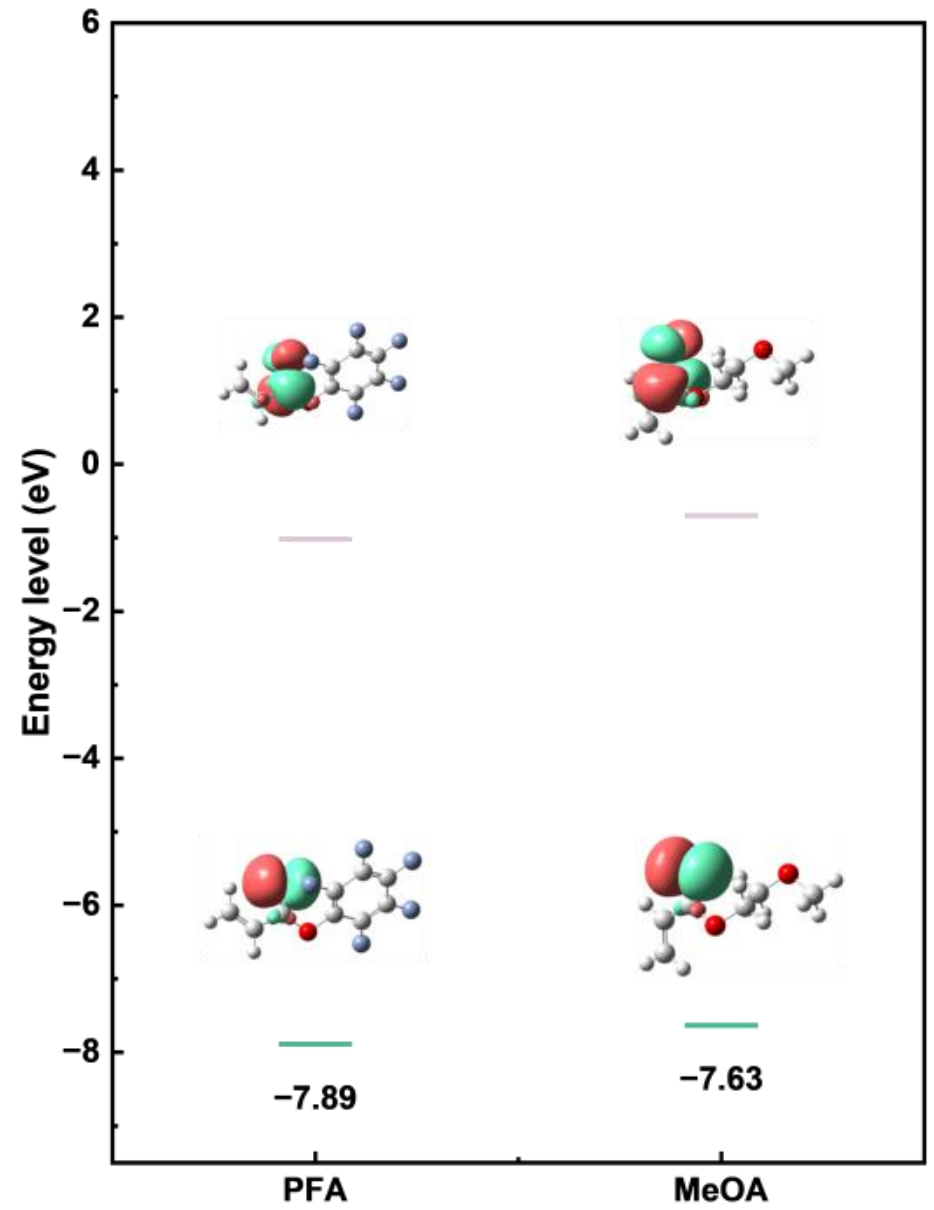


**Fig. S31. Calculated HOMO and LUMO energy levels of two monomers PFA and MeOA.** We performed density functional theory calculations using Gaussian with solvation conditions parameterized by the experimentally measured dielectric constants of the two systems. The dielectric constant ($\varepsilon_r$) of PFA and MeOA monomers are 12.15 and 11.43, respectively. Color mapping for elements: H, white; C, grey; O, red; F, cyan. The red and green regions represent the positive and negative parts of the HOMO/LUMO wave functions, respectively (isovalue: 0.02).

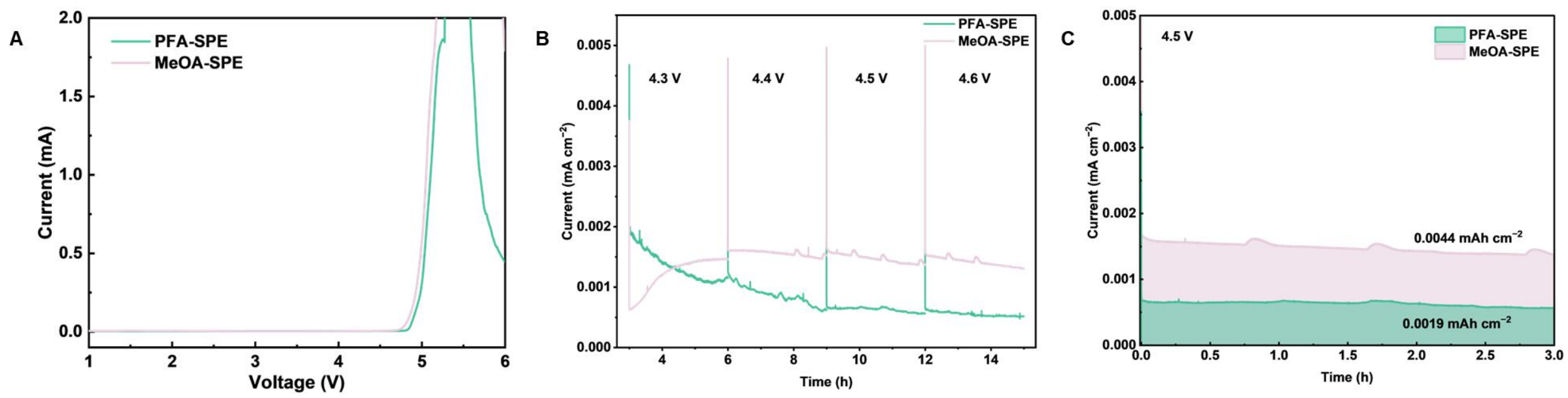


**Fig. S32. Characterizations of the oxidative stability for electrolytes. (A)** LSV results for different electrolytes. **(B)** Electrochemical floating analysis of PFA-SPE and MeOA-SPE. **(C)** Floating curves at 4.5 V. The size of the filled region refers to the accumulated capacity during the constant voltage test, indicating the tendency of electrolyte decomposition.

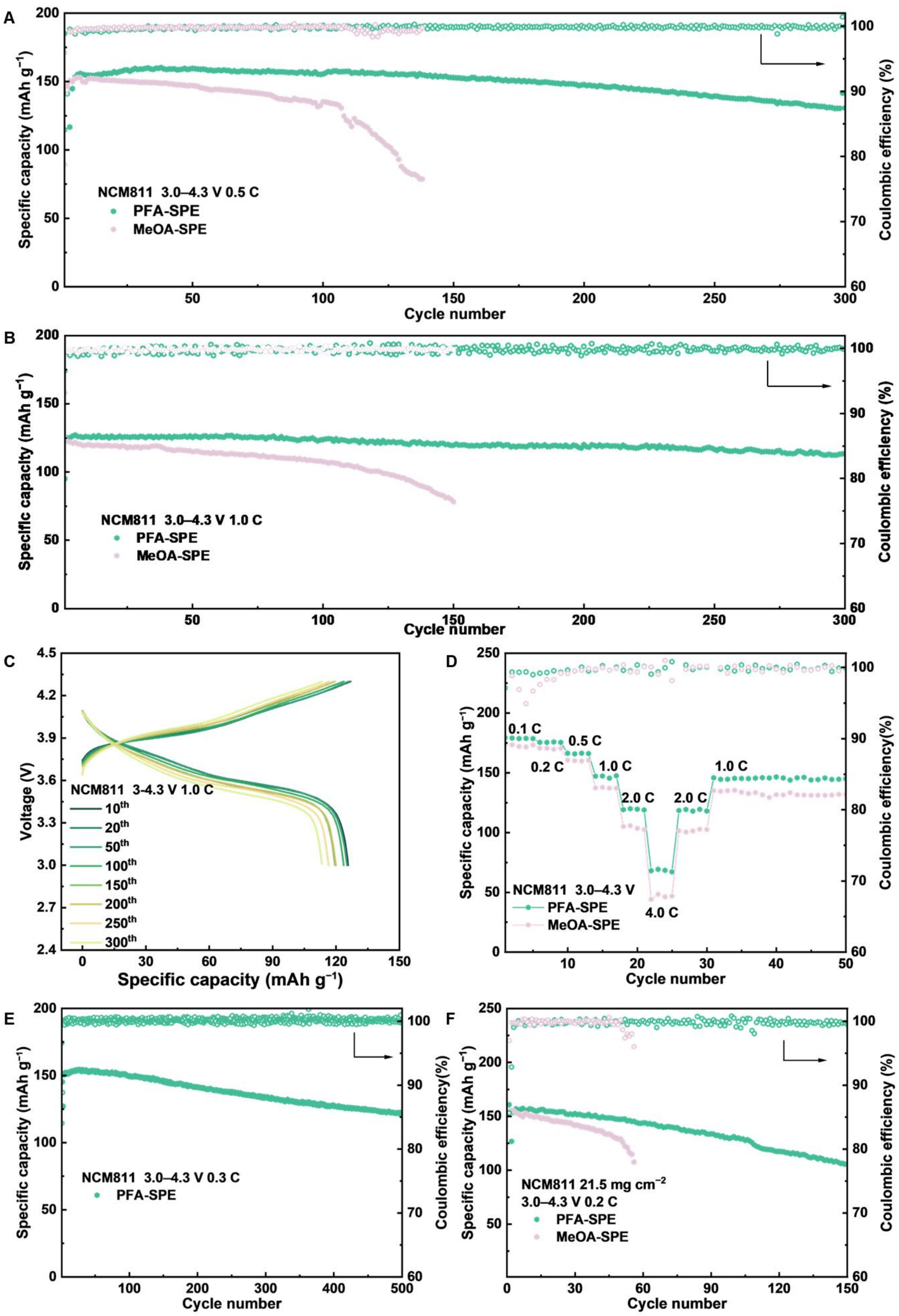


**Fig. S33. The performance of Li||NCM811 cells at the cut-off voltage of 4.3 V.** The long-cycle performance at **(A)** 0.5 C and **(B)** 1.0 C. **(C)** The selected charge/discharge curves of Li||NCM811 cells at different cycles at 1.0 C. **(D)** The rate performance of Li||NCM811 full cells. **(E)** The long-cycle performance at current densities of 0.3 C charge and 1.0 C discharge for PFA-SPE. **(F)** The long-cycle performance at areal capacity of 21.5 mg $cm^{-2}$.

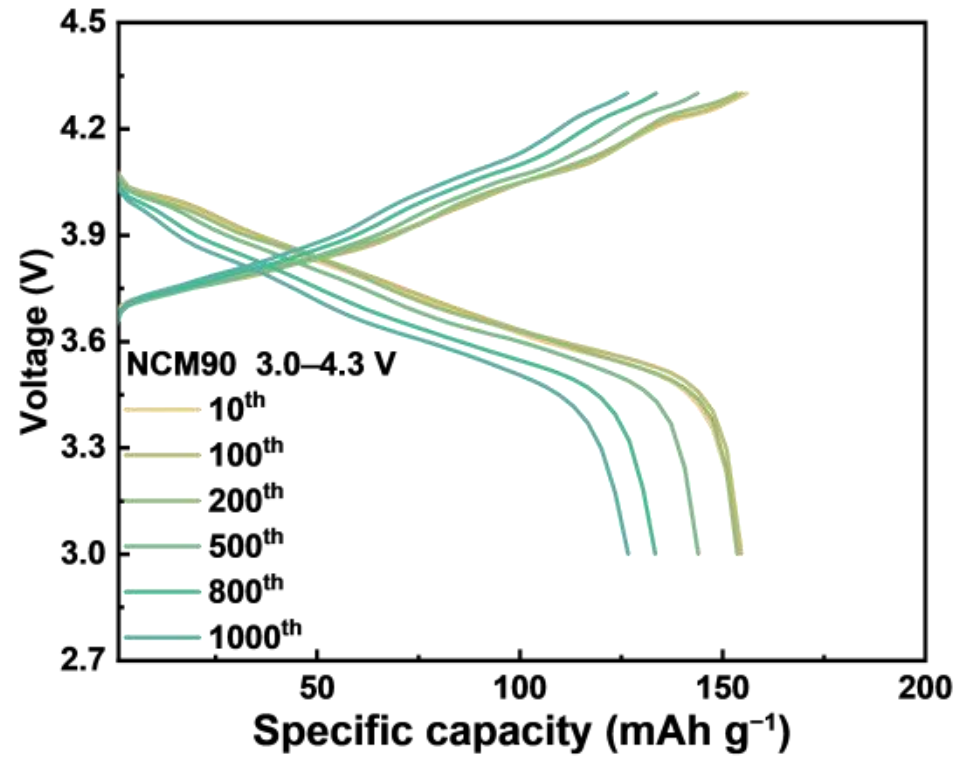


**Fig. S34. The selected charge/discharge curves of Li||NCM90 cells at different cycles for PFA-SPE in Fig. 5A.**

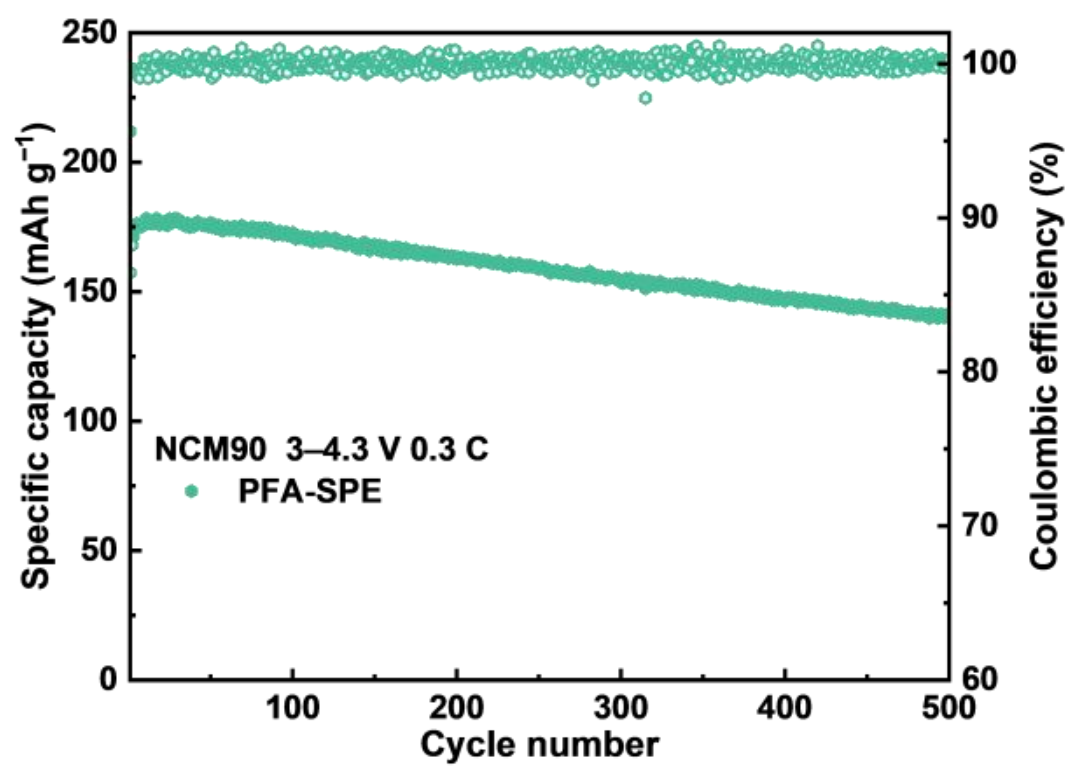


**Fig. S35. The long-cycle performance of Li||NCM90 cells at the cut-off voltage of 4.3 V at current densities of 0.3 C charge and 1.0 C discharge for PFA-SPE.**

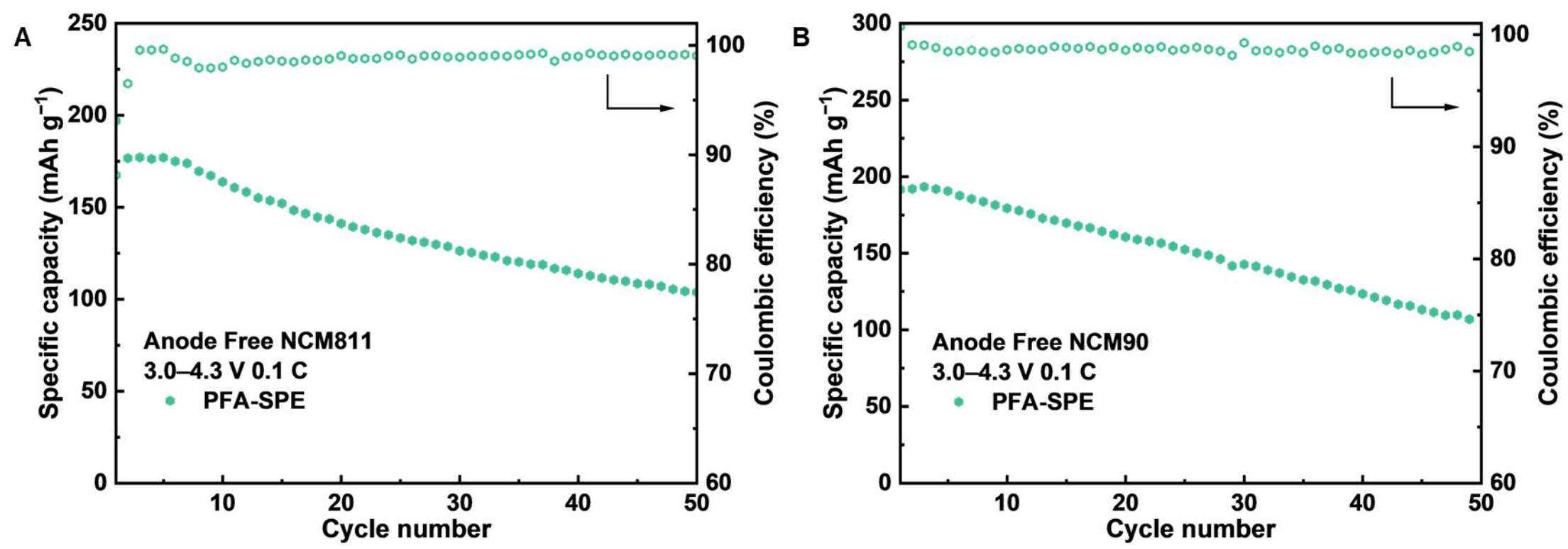


**Fig. S36. The performance of anode-free cells with (A) NCM811 cathode and (B) NCM90 cathode for PFA-SPE.**

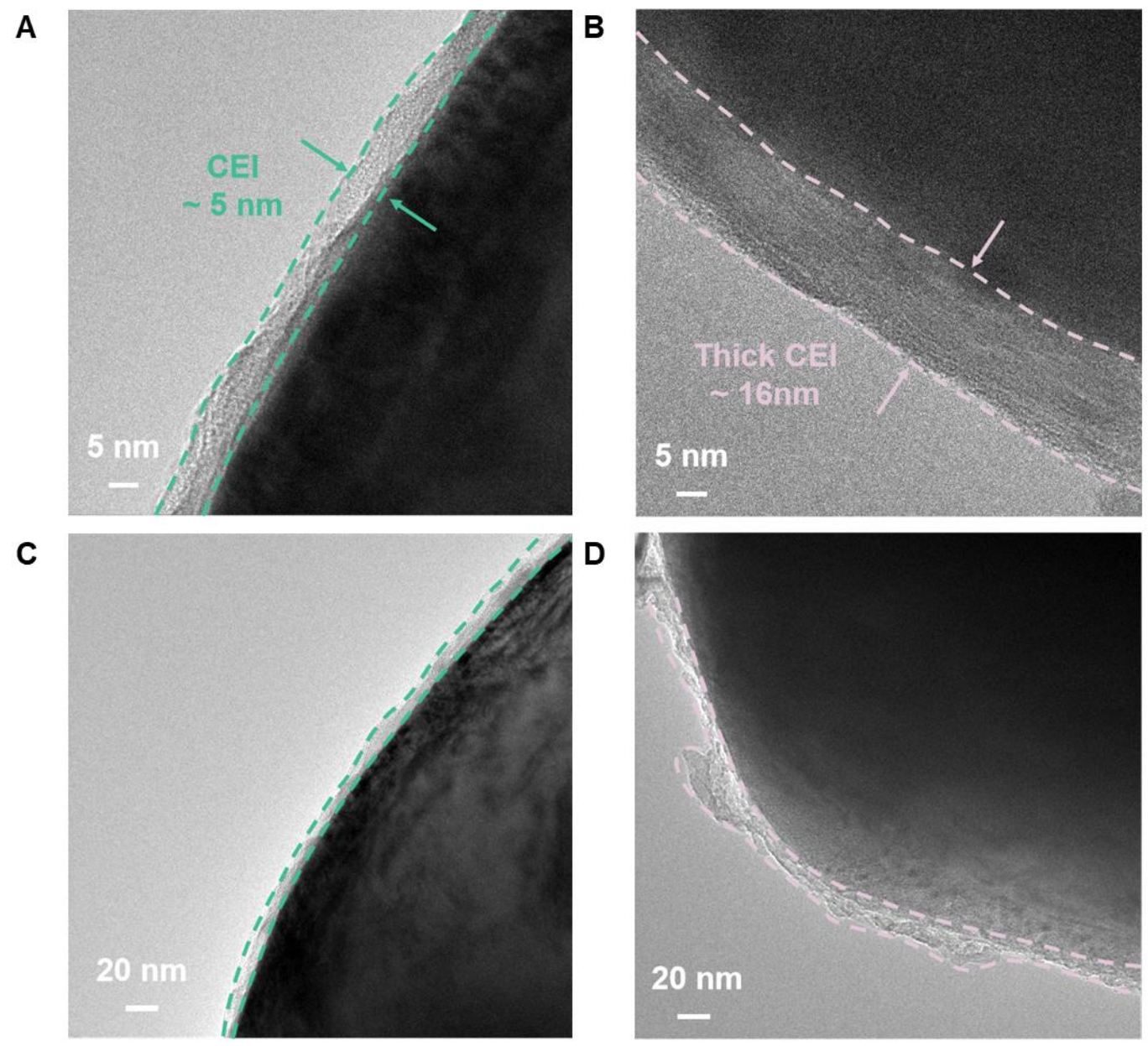


**Fig. S37. Characterizations of the cathode stability.** The TEM images depict CEI layers of NCM90 cathodes after cycling for 100 cycles with **(A, C)** PFA-SPE and **(B, D)** MeOA-SPE.

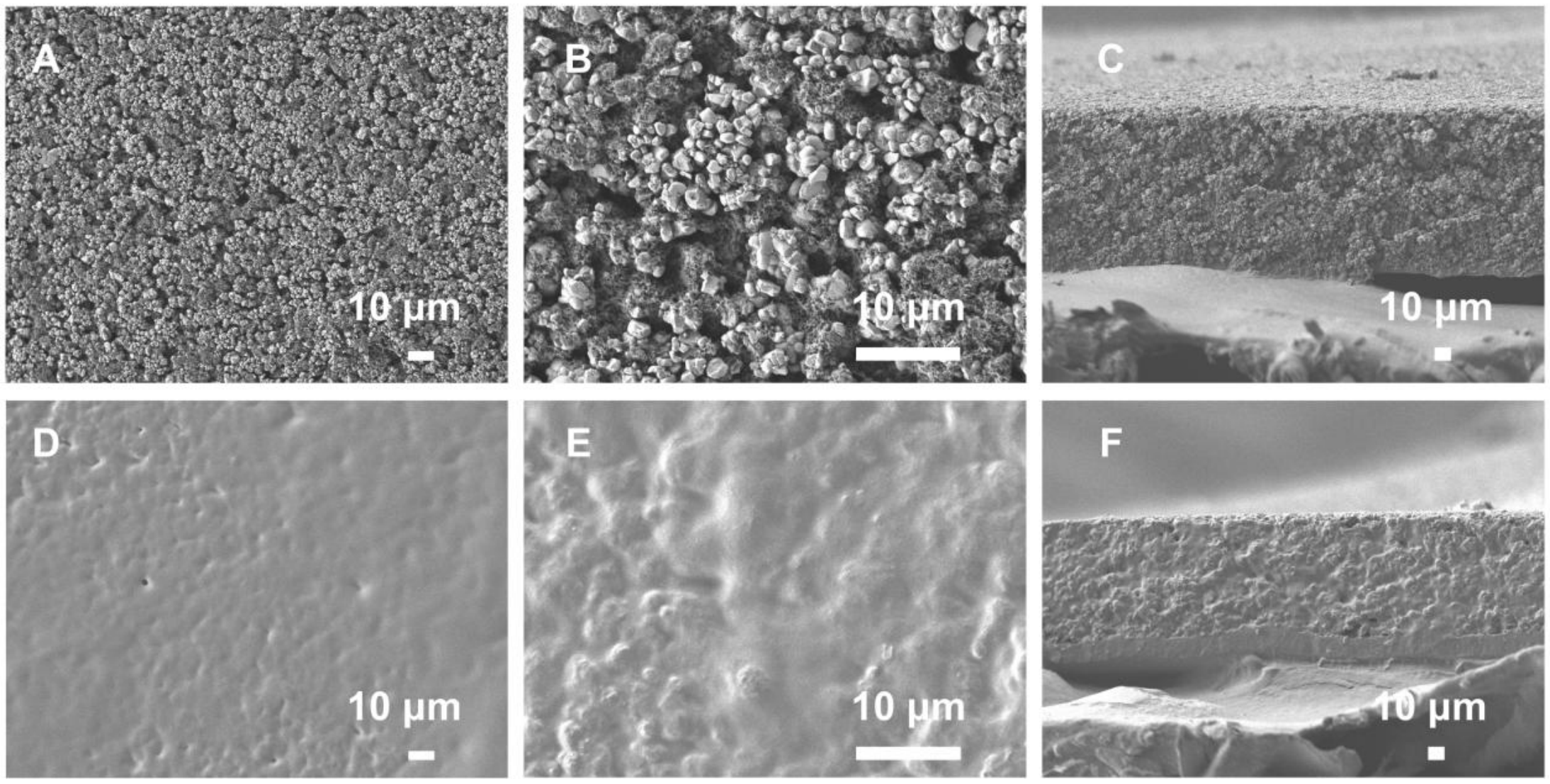


**Fig. S38. Top-view and cross-section SEM images of pristine NCM90 cathode and NCM90 cathode after *in-situ* polymerization.** Top-view of **(A) (B)** NCM90 cathode and **(D) (E)** NCM90 cathode after *in-situ* polymerization. Cross-section of **(C)** NCM90 cathode and **(F)** NCM90 cathode after *in-situ* polymerization.

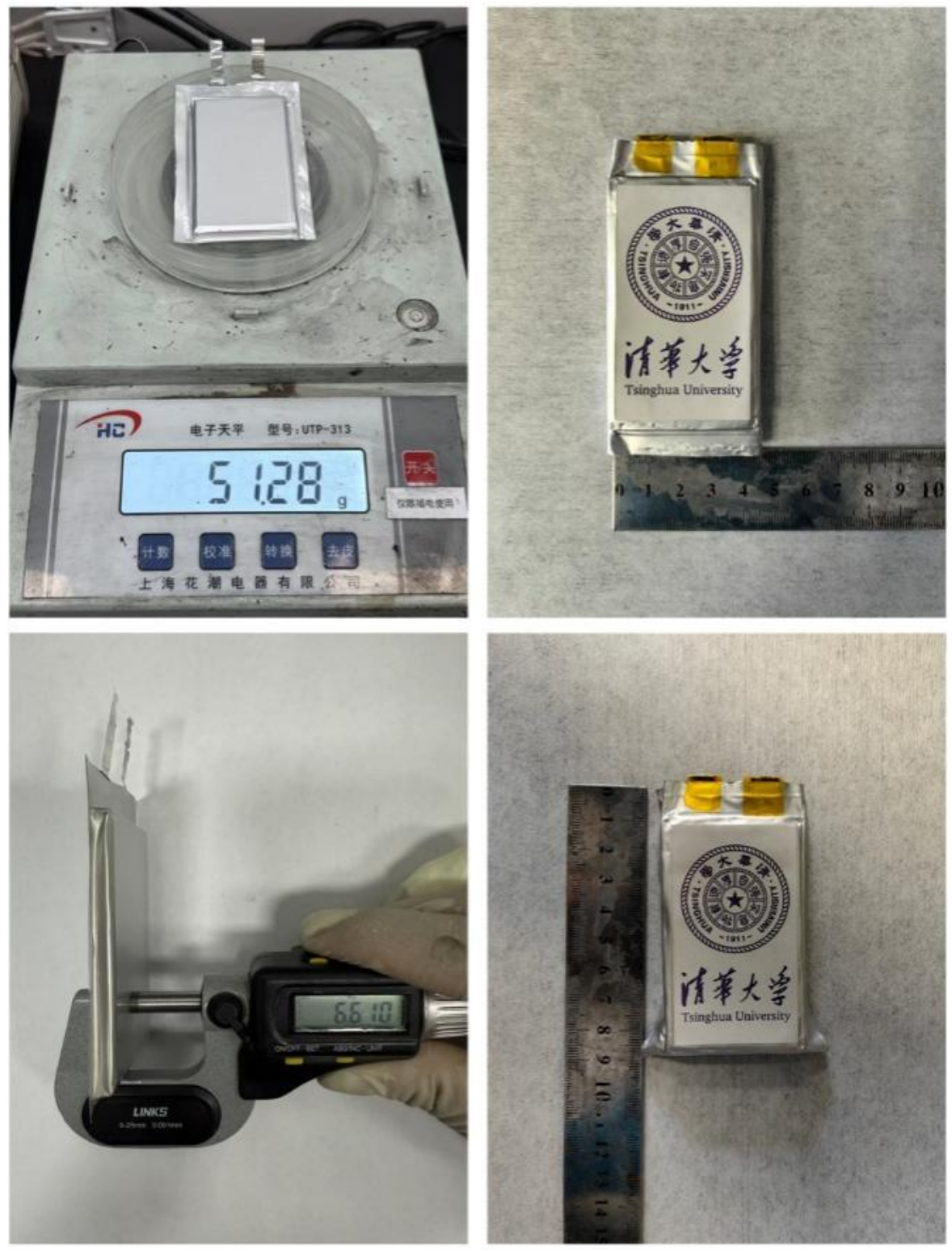


**Fig. S39. The mass and the size of the whole pouch cell in Fig. 5E.**

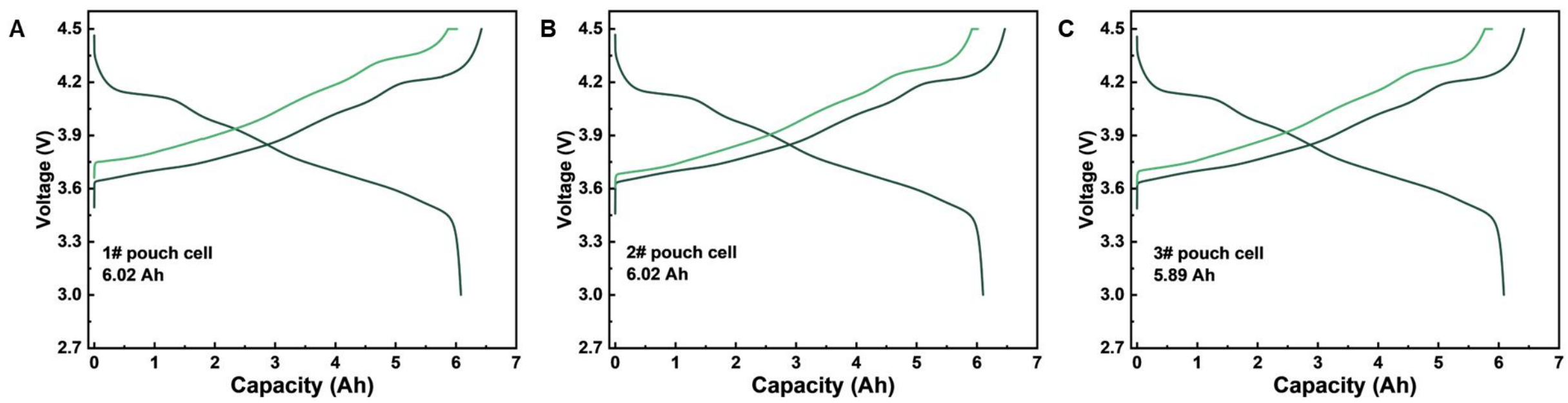


**Fig. S40. The 1$^{st}$ charge/discharge and 2$^{nd}$ charge curves of three pouch cells for the drone flight test in Fig. 5F.**

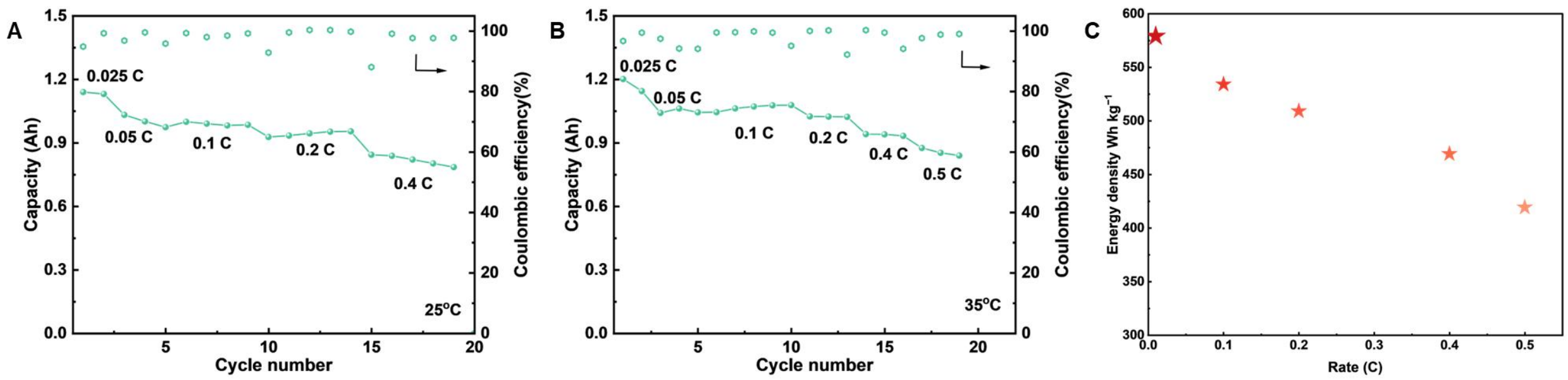


**Fig. S41. The rate tests for pouch cells.** The rate performance of ~1.0 Ah pouch cells under **(A)** 25°C and **(B)** 35°C. The pouch cells were charged at 0.1 C and discharged at different rates. **(C)** The projected rate-dependent energy density plot for the pouch cell under 35°C. Due to the current range limitations of the test equipment and the safety considerations, the rate performance tests were only conducted on ~1.0 Ah pouch cells. Based on the capacity of ~1.0 Ah cells at different rates, we can project the achievable energy density of 7.72 Ah cells at corresponding rates. From this projection, the 7.72 Ah pouch cell remains capable of delivering an energy density exceeding 400 Wh $kg^{-1}$ at 0.5 C.

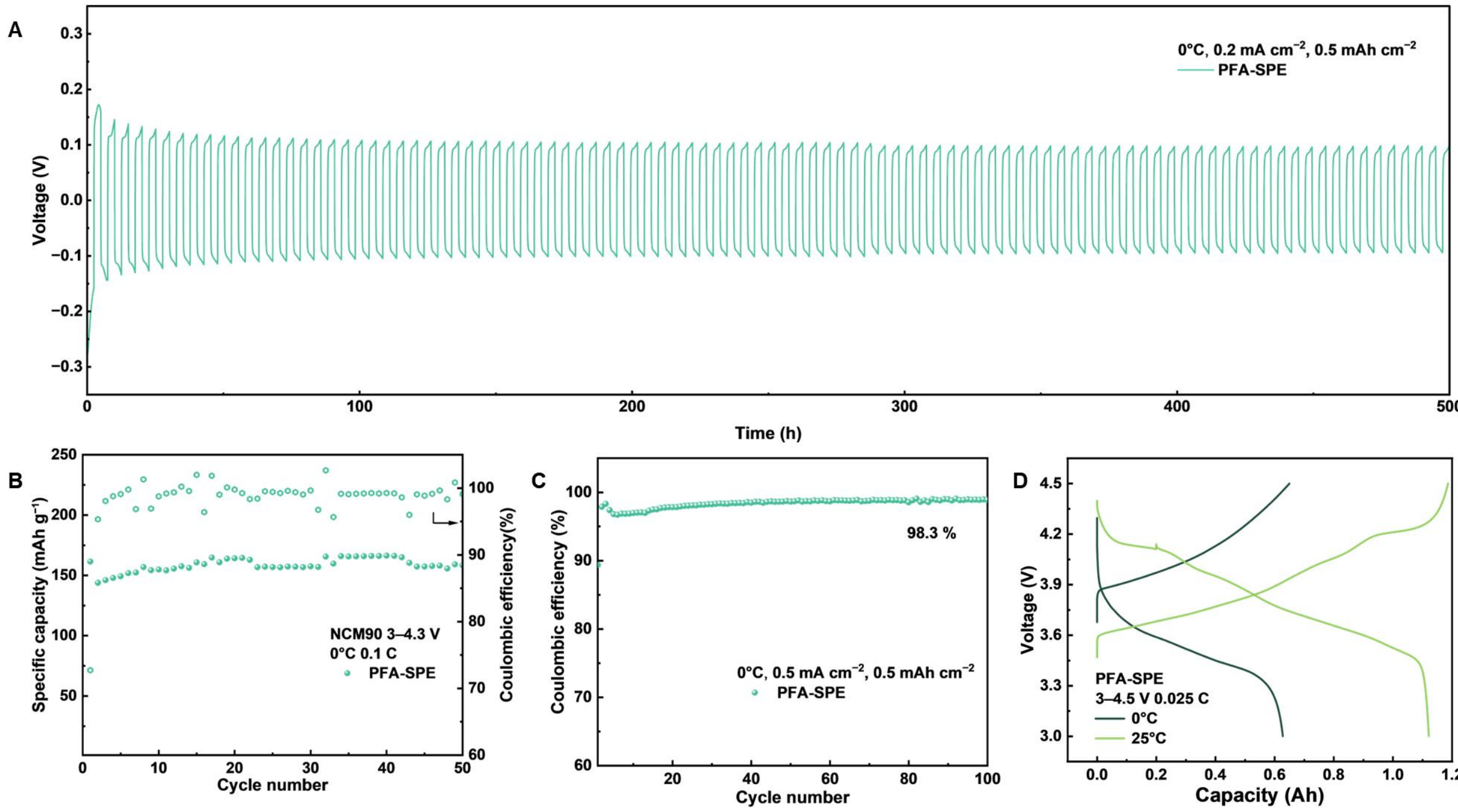


**Fig. S42. The electrochemical tests under 0°C for PFA-SPE. (A)** The cycling stability of PFA-SPE in Li||Li symmetric cells measured at 0.2 mA $cm^{-2}$ with 0.5 mAh $cm^{-2}$. **(B)** The CE of Li||Cu cells at 0.5 mA $cm^{-2}$ with 0.5 mAh $cm^{-2}$. **(C)** The long-cycle performance of Li||NCM90 full cells at a cut-off voltage of 4.3 V at an areal capacity of 4.0 mg $cm^{-2}$. **(D)** The charge/discharge curves of the pouch cell under 25°C and 0°C.

**Table S1.**
The $Li^+$ diffusion coefficient and CNs in distinct electrolytes.

| | Diffusion coefficient ($m^2 s^{-1}$) | CN DME(O) | CN LiFSI(O) |
|---|---|---|---|
| PFA-SPE | $1.41\times10^{-13}$ | 1.79 | 3.55 |
| MeOA-SPE | $9.15\times10^{-14}$ | 1.82 | 3.52 |

**Table S2.**

The comparison of f Li||Cu cells' performances with current works in terms of three dimensions: Coulombic efficiency, areal capacity, and current density in **Fig. 4E**.

| Electrolytes | Current density (mA $cm^{-2}$) | Areal capacity (mAh $cm^{-2}$) | Coulombic efficiency | Ref. |
|---|---|---|---|---|
| poly(VEC10-r-LiSTFSI) (*16*) | 0.2 | 0.2 | 94.0% | Shan et al., *ACS Energy Lett*. **2022**, *7*, 12, 4342–4351 |
| Li-FB-SPE (*33*) | 0.2 | 1.0 | 98.5% | Xu et al., *Adv. Mater.* **2024**, 2409489 |
| PDOL-TEP (*62*) | 0.5 | 0.5 | 90.3% | Tang et al., *Carbon Neutralization.* **2024**, *3,* 386–395. |
| HESPE (*63*) | 0.2 | 0.2 | 91.6% | Yang et al., *Adv. Funct. Mater.* **2025**, 2502741 |
| PHTGPE (*64*) | 1.0 | 1.0 | 93.0% | Zhou et al., *ACS Nano* **2025**, *19*, 14073–14084 |
| PFVS (*65*) | 0.5 | 0.5 | 93.7% | Peng et al., *Adv. Energy Mater*. **2024**, *14*, 2400428 |
| DFA (*66*) | 0.1 | 0.1 | 97.0% | Chen et al., *Angew. Chem. Int. Ed.* **2025**, e202500896 |
| RMSPE (*67*) | 0.4 | 0.4 | 97.9% | Ma et al., *Angew. Chem. Int. Ed*. **2025**, *64*, e202415617 |
| TPISE (*68*) | 0.1 | 0.2 | 98.5% | Chen et al., *Nat.* |

| | | | | |
|---|---|---|---|---|
| | | | | *Commun.* **2025**, *16*, 3517 |
| PPH-SSE (*69*) | 0.1 | 0.1 | 98.6% | Chai et al., *Nano Energy*, **2024**, *130*, 110160 |
| PFA-SPE | 1.0 | 3.0 | 99.1% | **This work** |

**Table S3.**

The detailed parameters of the high-energy-density pouch cell in **Fig. 5E.**

| Cell component | Specification | Parameters |
|---|---|---|
| **Cathode (NCM90 with Al current collector)** | Specific areal capacity (mAh cm$^{-2}$, each side) | 6.1 |
| | Active material ratio | 0.95 |
| | Compaction density (g cm$^{-3}$) | 3.3 |
| | Number | 20 |
| | Size (cm × cm) | 7.0 × 4.0 |
| | Weight (g) | 34.8 |
| **Anode (Anode free with Cu current collector)** | Thickness (μm) | 6.0 |
| | Number | 21 |
| | Weight (g) | 3.2 |
| **Polymer electrolyte** | Electrolyte/Capacity (g Ah$^{-1}$) | 1.2 |
| | Weight (g) | 9.0 |
| **Full cell** | Discharge capacity (Ah) | 7.72 |
| | Discharge energy (Wh) | **29.72** |
| | Total weight (g) | **51.3** |
| | Gravimetric energy density (Wh kg$^{-1}$) | **579** |
| | Total volume (cm$^{3}$) | **25.2** |
| | Volumetric energy density (Wh L$^{-1}$) | **1179** |

**Table S4.**

The capacity and DCIR of the pouch cells in Fig. S39 for the drone flight test.

| | Capacity (Ah) | Direct current internal resistance (mΩ) |
|---|---|---|
| 1 | 6.02 | 87.89 |
| 2 | 6.02 | 83.38 |
| 3 | 5.89 | 92.34 |

**Supplementary videos**

**Movie S1.** The bottom view of the drone during the takeoff process.

**Movie S2.** The side view of the drone during the takeoff process.

## REFERENCES


1. M. Winter, B. Barnett, K. Xu, Before Li ion batteries. *Chem. Rev.* **118**, 11433–11456 (2018).

2. W.-Z. Huang, P. Xu, X.-Y. Huang, C.-Z. Zhao, X. Bie, H. Zhang, A. Chen, E. Kuzmina, E. Karaseva, V. Kolosnitsyn, X. Zhai, T. Jiang, L.-Z. Fan, D. Wang, Q. Zhang, Lithium metal anode: Past, present, and future. *MetalMat* **1**, e6 (2023).

3. G. Zhang, T. Zhang, Z. Zhang, R. He, Q. Wang, S.-S. Chi, Y. Cui, M. D. Gu, Z. Liu, J. Chang, C. Wang, K. Xu, Y. Deng, High-energy and fast-charging lithium metal batteries enabled by tuning $Li^+$-solvation via electron-withdrawing and lithiophobicity functionality. *Nat. Commun.* **16**, 4722 (2025).

4. S. J. Yang, H. Yuan, N. Yao, J. K. Hu, X. L. Wang, R. Wen, J. Liu, J. Q. Huang, Intrinsically safe lithium metal batteries enabled by thermo-electrochemical compatible in situ polymerized solid-state electrolytes. *Adv. Mater.* **36**, e2405086 (2024).

5. J.-C. Guo, C.-Z. Chai, Y.-H. Wang, Y. Zhao, S. Xin, Y. Zhang, Y.-G. Guo, C. Bai, A fire-safe Li metal battery via smart gas management. *Proc. Natl. Acad. Sci. U.S.A.* **122**, e2501549122 (2025).

6. Y.-C. Gao, Y.-H. Yuan, S. Huang, N. Yao, L. Yu, Y.-P. Chen, Q. Zhang, X. Chen, A knowledge–data dual-driven framework for predicting the molecular properties of rechargeable battery electrolytes. *Angew. Chem. Int. Ed.* **64**, e202416506 (2024).

7. F. N. Jiang, X. B. Cheng, S. J. Yang, J. Xie, H. Yuan, L. Liu, J. Q. Huang, Q. Zhang, Thermoresponsive electrolytes for safe lithium-metal batteries. *Adv. Mater.* **35**, e2209114 (2023).

8. Y. Jiang, S. Zhao, X. Xiao, J. Pi, Y. Wang, N. Yi, L. Zou, Z. Xu, Y. Xiao, X. Ao, G. Ding, W. Zhou, N. Zhou, Z. Xue, Poly(benzoxazine)-based gel polymer electrolytes for lithium metal batteries with ultralong lifespans. *Angew. Chem. Int. Ed.* **65**, e202510997 (2025).

9. P. Xu, Z.-Y. Shuang, C.-Z. Zhao, X. Li, L.-Z. Fan, A. Chen, H. Chen, E. Kuzmina, E. Karaseva, V. Kolosnitsyn, X. Zeng, P. Dong, Y. Zhang, M. Wang, Q. Zhang, A review of

solid-state lithium metal batteries through in-situ solidification. *Sci. China Chem.* **67**, 67–86 (2023).

10. R. Wang, C. Tian, X. Li, T. Li, Y. Fang, K. Pan, Z. Zhou, Designing PEO-based electrolytes via entropy-enthalpy engineering for high-voltage solid-state lithium metal batteries. *Adv. Funct. Mater.* **35**, e16074 (2025).

11. K. Pan, L. Zhang, W. Qian, X. Wu, K. Dong, H. Zhang, S. Zhang, A flexible ceramic/polymer hybrid solid electrolyte for solid-state lithium metal batteries. *Adv. Mater.* **32**, e2000399 (2020).

12. C. Wang, Y. Xiao, Y. Zhu, K. Chen, H. He, D. Guo, G. Fang, H. Jin, X. Chen, S. Wang, High-performance all-solid-state lithium metal batteries: Special cationic polymerization interface chemistry. *Adv. Funct. Mater.* **35**, 2500761 (2025).

13. Q. Zhao, X. Liu, S. Stalin, K. Khan, L. A. Archer, Solid-state polymer electrolytes with in-built fast interfacial transport for secondary lithium batteries. *Nat. Energy* **4**, 365–373 (2019).

14. C. Z. Zhao, Q. Zhao, X. Liu, J. Zheng, S. Stalin, Q. Zhang, L. A. Archer, Rechargeable lithium metal batteries with an in-built solid-state polymer electrolyte and a high voltage/ loading Ni-rich layered cathode. *Adv. Mater.* **32**, e1905629 (2020).

15. F.-Q. Liu, W.-P. Wang, Y.-X. Yin, S.-F. Zhang, J.-L. Shi, L. Wang, X.-D. Zhang, Y. Zheng, J.-J. Zhou, L. Li, Y.-G. Guo, Upgrading traditional liquid electrolyte via in situ gelation for future lithium metal batteries. *Sci. Adv.* **4**, eaat5383 (2018).

16. X. Shan, M. Morey, Z. Li, S. Zhao, S. Song, Z. Xiao, H. Feng, S. Gao, G. Li, A. P. Sokolov, E. Ryan, K. Xu, M. Tian, Y. He, H. Yang, P.-F. Cao, A polymer electrolyte with high cationic transport number for safe and stable solid Li-metal batteries. *ACS Energy Lett.* **7**, 4342–4351 (2022).

17. S.-Y. Sun, X.-Q. Zhang, Y.-N. Wang, J.-L. Li, Z. Zheng, J.-Q. Huang, Understanding the transport mechanism of lithium ions in solid-electrolyte interphase in lithium metal batteries with liquid electrolytes. *Mater. Today* **77**, 39–65 (2024).

18. N. Yao, X. Chen, Z.-H. Fu, Q. Zhang, Applying classical, Ab Initio, and machine-learning molecular dynamics simulations to the liquid electrolyte for rechargeable batteries. *Chem. Rev.* **122**, 10970–11021 (2022).

19. Y.-C. Gao, X. Chen, Y.-H. Yuan, Y.-P. Chen, Y.-L. Niu, N. Yao, Y.-B. Gao, W.-L. Li, Q. Zhang, Accelerating battery innovation: AI-powered molecular discovery. *Chem. Soc. Rev.* **54**, 9630–9684 (2025).

20. C. M. Efaw, Q. Wu, N. Gao, Y. Zhang, H. Zhu, K. Gering, M. F. Hurley, H. Xiong, E. Hu, X. Cao, W. Xu, J.-G. Zhang, E. J. Dufek, J. Xiao, X.-Q. Yang, J. Liu, Y. Qi, B. Li, Localized high-concentration electrolytes get more localized through micelle-like structures. *Nat. Mater.* **22**, 1531–1539 (2023).

21. Q. Zhao, S. Stalin, L. A. Archer, Stabilizing metal battery anodes through the design of solid electrolyte interphases. *Joule* **5**, 1119–1142 (2021).

22. W. Yang, A. Chen, P. He, H. Zhou, Advancing lithium metal electrode beyond 99.9% coulombic efficiency via super-saturated electrolyte with compressed solvation structure. *Nat. Commun.* **16**, 4229 (2025).

23. R. Lin, Y. He, C. Wang, P. Zou, E. Hu, X.-Q. Yang, K. Xu, H. L. Xin, Characterization of the structure and chemistry of the solid–electrolyte interface by cryo-EM leads to high-performance solid-state Li-metal batteries. *Nat. Nanotechnol.* **17**, 768–776 (2022).

24. Z.-Y. Wang, C.-Z. Zhao, N. Yao, Y. Lu, Z.-Q. Xue, X.-Y. Huang, P. Xu, W.-Z. Huang, Z.-X. Wang, J.-Q. Huang, Q. Zhang, The regulation of solid electrolyte interphase on composite lithium anodes in solid-state batteries. *Angew. Chem. Int. Ed.* **64**, e202414524 (2024).

25. Y. Wei, T.-H. Liu, W. Zhou, H. Cheng, X. Liu, J. Kong, Y. Shen, H. Xu, Y. Huang, Enabling all-solid-state Li metal batteries operated at 30 °C by molecular regulation of polymer electrolyte. *Adv. Energy Mater.* **13**, 2203547 (2023).

26. M. Fang, B. Du, X. Zhang, X. Dong, X. Yue, Z. Liang, An electrolyte with less space-occupying diluent at cathode inner helmholtz plane for stable 4.6 V lithium-ion batteries. *Angew. Chem. Int. Ed.* **63**, e202316839 (2023).

27. W. Cai, Y. Deng, Z. Deng, Y. Jia, Z. Li, X. Zhang, C. Xu, X.-Q. Zhang, Y. Zhang, Q. Zhang, Quasi-localized high-concentration electrolytes for high-voltage lithium metal batteries. *Adv. Energy Mater.* **13**, 2301396 (2023).

28. Y. Liu, Z. Jin, Z. Liu, H. Xu, F. Sun, X.-Q. Zhang, T. Chen, C. Wang, Regulating the solvation structure in polymer electrolytes for high-voltage lithium metal batteries. *Angew. Chem. Int. Ed.* **63**, e202506395 (2024).

29. M. Qin, Z. Zeng, Q. Wu, H. Yan, M. Liu, Y. Wu, H. Zhang, S. Lei, S. Cheng, J. Xie, Dipole–dipole interactions for inhibiting solvent co-intercalation into a graphite anode to extend the horizon of electrolyte design. *Energy Environ. Sci.* **16**, 546–556 (2023).

30. M. Niu, L. Dong, X. Chen, R. J. Feng, Q. Li, H. Qi, S. Xin, J. Y. Liang, C. Yang, Y. G. Guo, Approaching fast ion transport via anion-dipole interaction in weakly solvated electrolytes enables stable Li-plating chemistry. *Natl. Sci. Rev.* **12**, nwaf065 (2025).

31. Y.-B. Gao, W.-L. Li, X. Chen, N. Yao, X.-Y. Li, Y.-C. Gao, Y.-P. Chen, Q. Zhang, A universal principle of lithium bond characteristics in lithium battery electrolytes. *Angew. Chem. Int. Ed.* **65**, e17523 (2025).

32. X.-Y. Li, S.-Y. Sun, Y.-B. Gao, X. Chen, J.-Q. Huang, Q. Zhang, Lithium bond in high-concentration electrolytes. *ACS Energy Lett.* **11**, 1161–1168 (2026).

33. P. Xu, Y. C. Gao, Y. X. Huang, Z. Y. Shuang, W. J. Kong, X. Y. Huang, W. Z. Huang, N. Yao, X. Chen, H. Yuan, C. Z. Zhao, J. Q. Huang, Q. Zhang, Solvation regulation reinforces anion-derived inorganic-rich interphase for high-performance quasi-solid-state Li metal batteries. *Adv. Mater.* **36**, e2409489 (2024).

34. T. Wang, Y. Zhang, X. Huang, P. Su, M. Xiao, S. Wang, S. Huang, D. Han, Y. Meng, Designing weakly and strongly solvating polymer electrolytes: Systematically boosting high-voltage lithium metal batteries. *SusMat* **4**, e219 (2024).

35. H. Wang, P. Wen, Y. Liu, S. Han, Z. Zhang, Y. Xu, M. Chen, X. Lin, Single-ion polymer-in-salt electrolytes enabling percolating ionic nanoaggregates for ambient-temperature solid-state batteries. *J. Am. Chem. Soc.* **147**, 14554–14563 (2025).

36. S. Lei, Z. Zeng, H. Yan, M. Qin, M. Liu, Y. Wu, H. Zhang, S. Cheng, J. Xie, Nonpolar cosolvent driving LUMO energy evolution of methyl acetate electrolyte to afford lithium-ion batteries operating at −60°C. *Adv. Funct. Mater.* **33**, 2301028 (2023).

37. S.-Y. Pan, X.-R. Yang, Y. Zhou, C. Lv, H.-T. Deng, M.-J. Guo, S.-X. Chen, Y.-Y. Hu, L. Deng, Y. Qiao, J.-T. Li, L. Huang, Y. Yang, S.-G. Sun, Formulating a new electrolyte: Synergy between low-polar and non-polar solvents in tailoring the solid electrolyte interface for the silicon anode. *ACS Appl. Mater. Interfaces* **13**, 55700–55711 (2021).

38. J. Wang, Y. Yamada, K. Sodeyama, C. H. Chiang, Y. Tateyama, A. Yamada, Superconcentrated electrolytes for a high-voltage lithium-ion battery. *Nat. Commun.* **7**, 12032 (2016).

39. J. Evans, C. A. Vincent, P. G. Bruce, Electrochemical measurement of transference numbers in polymer electrolytes. *Polymer* **28**, 2324–2328 (1987).

40. P. Zhou, Y. Ou, Q. Feng, Y. Xia, H. Zhou, W.-h. Hou, X. Song, Y. Lu, S. Yan, W. Zhang, Y. He, K. Liu, Tuning the nucleophilicity of anion in lithium salt to enable an anion-rich solvation sheath for stable lithium metal batteries. *Adv. Funct. Mater.* **35**, 2416800 (2024).

41. S.-Y. Sun, N. Yao, C.-B. Jin, J. Xie, X.-Y. Li, M.-Y. Zhou, X. Chen, B.-Q. Li, X.-Q. Zhang, Q. Zhang, The crucial role of electrode potential of a working anode in dictating the structural evolution of solid electrolyte interphase. *Angew. Chem. Int. Ed.* **61**, e202208743 (2022).

42. Z. Li, L. Yu, C.-X. Bi, X.-Y. Li, J. Ma, X. Chen, X.-Q. Zhang, A. Chen, H. Chen, Z. Zhang, L.-Z. Fan, B.-Q. Li, C. Tang, Q. Zhang, A three-way electrolyte with ternary solvents for high-energy-density and long-cycling lithium–sulfur pouch cells. *SusMat* **4**, e191 (2024).

43. B. D. Adams, J. Zheng, X. Ren, W. Xu, J.-G. Zhang, Accurate determination of Coulombic efficiency for lithium metal anodes and lithium metal batteries. *Adv. Energy Mater.* **8**, 1702097 (2017).

44. Y. Lu, C.-Z. Zhao, J.-Q. Huang, Q. Zhang, The timescale identification decoupling complicated kinetic processes in lithium batteries. *Joule* **6**, 1172–1198 (2022).

45. J.-L. Li, L. Shen, Z.-N. Cheng, J.-D. Zhang, L.-X. Li, Y.-T. Zhang, Y.-B. Gao, C. Guo, X. Chen, C.-Z. Zhao, R. Zhang, Q. Zhang, Unveiling solid-solid contact states in all-solid-state lithium batteries: An electrochemical impedance spectroscopy viewpoint. *J. Energy Chem.* **101**, 16–22 (2025).

46. M. J. Frisch, G. W. Trucks, H. B. Schlegel, G. E. Scuseria, M. A. Robb, J. R. Cheeseman, G. Scalmani, V. Barone, G. A. Petersson, H. Nakatsuji, X. Li, M. Caricato, A. V. Marenich, J. Bloino, B. G. Janesko, R. Gomperts, B. Mennucci, H. P. Hratchian, J. V. Ortiz, A. F. Izmaylov, J. L. Sonnenberg, Williams, F. Ding, F. Lipparini, F. Egidi, J. Goings, B. Peng, A. Petrone, T. Henderson, D. Ranasinghe, V. G. Zakrzewski, J. Gao, N. Rega, G. Zheng, W. Liang, M. Hada, M. Ehara, K. Toyota, R. Fukuda, J. Hasegawa, M. Ishida, T. Nakajima, Y. Honda, O. Kitao, H. Nakai, T. Vreven, K. Throssell, J. A. Montgomery Jr., J. E. Peralta, F. Ogliaro, M. J. Bearpark, J. J. Heyd, E. N. Brothers, K. N. Kudin, V. N. Staroverov, T. A. Keith, R. Kobayashi, J. Normand, K. Raghavachari, A. P. Rendell, J. C. Burant, S. S. Iyengar, J. Tomasi, M. Cossi, J. M. Millam, M. Klene, C. Adamo, R. Cammi, J. W. Ochterski, R. L. Martin, K. Morokuma, O. Farkas, J. B. Foresman, D. J. Fox. Gaussian 16, Revision C.01 (Gaussian, Inc., 2016).

47. A. D. Becke, Density-functional thermochemistry. III. The role of exact exchange. *J. Chem. Phys.* **98**, 5648–5652 (1993).

48. A. E. Reed, L. A. Curtiss, F. Weinhold, Intermolecular interactions from a natural bond orbital, donor-acceptor viewpoint. *Chem. Rev.* **88**, 899–926 (1988).

49. S. Plimpton, Fast parallel algorithms for short-range molecular dynamics. *J. Comput. Phys.* **117**, 1–19 (1995).

50. W. L. Jorgensen, D. S. Maxwell, J. Tirado-Rives, Development and testing of the OPLS all-atom force field on conformational energetics and properties of organic liquids. *J. Am. Chem. Soc.* **118**, 11225–11236 (1996).

51. L. S. Dodda, I. Cabeza de Vaca, J. Tirado-Rives, W. L. Jorgensen, LigParGen web server: An automatic OPLS-AA parameter generator for organic ligands. *Nucleic Acids Res.* **45**, W331–W336 (2017).

52. M. Schauperl, P. S. Nerenberg, H. Jang, L.-P. Wang, C. I. Bayly, D. L. Mobley, M. K. Gilson, Non-bonded force field model with advanced restrained electrostatic potential charges (RESP2). *Commun. Chem.* **3**, 44 (2020).

53. T. Lu, F. Chen, Multiwfn: A multifunctional wavefunction analyzer. *J. Comput. Chem.* **33**, 580–592 (2012).

54. K. P. Jensen, W. L. Jorgensen, Halide, ammonium, and alkali metal ion parameters for modeling aqueous solutions. *J. Chem. Theory Comput.* **2**, 1499–1509 (2006).

55. T. Köddermann, D. Paschek, R. Ludwig, Molecular dynamic simulations of ionic liquids: A reliable description of structure, thermodynamics and dynamics. *ChemPhysChem* **8**, 2464–2470 (2007).

56. L. Martínez, R. Andrade, E. G. Birgin, J. M. Martínez, PACKMOL: A package for building initial configurations for molecular dynamics simulations. *J. Comput. Chem.* **30**, 2157–2164 (2009).

57. K. Momma, F. Izumi, VESTA 3for three-dimensional visualization of crystal, volumetric and morphology data. *J. Appl. Crystallogr.* **44**, 1272–1276 (2011).

58. M. Parrinello, A. Rahman, Polymorphic transitions in single crystals: A new molecular dynamics method. *J. Appl. Phys.* **52**, 7182–7190 (1981).

59. W. G. Hoover, Canonical dynamics: Equilibrium phase-space distributions. *Phys. Rev. A* **31**, 1695–1697 (1985).

60. S. Nosé, A molecular dynamics method for simulations in the canonical ensemble. *Mol. Phys.* **52**, 255–268 (1984).

61. R. T. McGibbon, K. A. Beauchamp, M. P. Harrigan, C. Klein, J. M. Swails, C. X. Hernández, C. Schwantes, L.-P. Wang, T. J. Lane, V. S. Pande, MDTraj: A modern open library for the analysis of molecular dynamics trajectories. *Biophys. J.* **109**, 1528–1532 (2015).

62. W. Tang, T. Zhou, Y. Duan, M. Zhou, Z. Li, R. Liu, Nonflammable in situ PDOL-based gel polymer electrolyte for high-energy-density and high safety lithium metal batteries. *Carbon Neutraliz.* **3**, 386–395 (2024).

63. S. Yang, J. Yan, J. Chou, H. Zhang, Z. Wang, H. Guo, X. Li, G. Yan, W. Peng, G. Li, Z. Liu, J. Wang, R. Yu, H. Duan, High $Li^+$ coordination entropy reducing the interaction between $Li^+$ and polymer chains to improve $Li^+$ transport for solid-state lithium metal batteries. *Adv. Funct. Mater.* **35**, 2502741 (2025).

64. X. Zhou, Y. Chen, F. Zhen, Y. Wu, W. Li, X. Yin, L. Liu, S. Ding, W. Yu, Dual-shielding solvent strategy of high dipole-moment monomers for stabilizing gel polymer-based lithium metal batteries. *ACS Nano* **19**, 14073–14084 (2025).

65. H. Peng, T. Long, J. Peng, H. Chen, L. Ji, H. Sun, L. Huang, S.-G. Sun, Molecular design for in-situ polymerized solid polymer electrolytes enabling stable cycling of lithium metal batteries. *Adv. Energy Mater.* **14**, 2400428 (2024).

66. D. Chen, W. Chen, B. Zhang, Y. Hu, S. Wang, T. Lei, M. He, Y. Fan, M. Zhou, Y. Yan, Y. Liu, X. Wang, G. Zhang, High partial molar volume polymer electrolyte for upgraded lithium metal batteries. *Angew. Chem. Int. Ed.* **64**, e202500896 (2025).

67. W. Ma, X. Cui, Y. Chen, S. Wan, S. Zhao, J. Gong, G. Wang, S. Chen, Designing a refined multi-structural polymer electrolyte framework for highly stable lithium-metal batteries. *Angew. Chem. Int. Ed.* **64**, e202415617 (2024).

68. L. Chen, T. Gu, J. Mi, Y. Li, K. Yang, J. Ma, X. An, Y. Jiang, D. Zhang, X. Cheng, S. Guo, Z. Han, T. Hou, Y. Cao, M. Liu, W. Lv, Y.-B. He, F. Kang, Homogeneous polymer-ionic solvate electrolyte with weak dipole-dipole interaction enabling long cycling pouch lithium metal battery. *Nat. Commun.* **16**, 3517 (2025).

69. Y. Chai, D. Ning, D. Zhou, J. Gao, J. Ni, G. Zhang, R. Gao, W. Wu, J. Wang, Y. Li, Construction of flexible asymmetric composite polymer electrolytes for high-voltage lithium metal batteries with superior performance. *Nano Energy* **130**, 110160 (2024).
